# Genetically encoded phosphatidylserine biosensor for in vitro, ex vivo and in vivo labelling

**DOI:** 10.1186/s11658-023-00472-7

**Published:** 2023-07-27

**Authors:** Eimina Dirvelyte, Daina Bujanauskiene, Evelina Jankaityte, Neringa Daugelaviciene, Ugne Kisieliute, Igor Nagula, Rima Budvytyte, Urte Neniskyte

**Affiliations:** 1 VU LSC-EMBL Partnership Institute for Genome Editing Technologies, Life Sciences Center, Vilnius University, Vilnius, Lithuania; 2 Institute of Bioscience, Life Sciences Center, Vilnius University, Vilnius, Lithuania; 3 Institute of Biochemistry, Life Sciences Center, Vilnius University, Vilnius, Lithuania

**Keywords:** Phosphatidylserine, Genetically encoded probe, Lactadherin, MFG-E8, C2 domain, SNAP-tag

## Abstract

**Background:**

The dynamics of phosphatidylserine in the plasma membrane is a tightly regulated feature of eukaryotic cells. Phosphatidylserine (PS) is found preferentially in the inner leaflet of the plasma membrane. Disruption of this asymmetry leads to the exposure of phosphatidylserine on the cell surface and is associated with cell death, synaptic pruning, blood clotting and other cellular processes. Due to the role of phosphatidylserine in widespread cellular functions, an efficient phosphatidylserine probe is needed to study them. Currently, a few different phosphatidylserine labelling tools are available; however, these labels have unfavourable signal-to-noise ratios and are difficult to use in tissues due to limited permeability. Their application in living tissue requires injection procedures that damage the tissue and release damage-associated molecular patterns, which in turn stimulates phosphatidylserine exposure.

**Methods:**

For this reason, we developed a novel genetically encoded phosphatidylserine probe based on the C2 domain of the lactadherin (MFG-E8) protein, suitable for labelling exposed phosphatidylserine in various research models. We tested the C2 probe specificity to phosphatidylserine on hybrid bilayer lipid membranes by observing surface plasmon resonance angle shift. Then, we analysed purified fused C2 proteins on different cell culture lines or engineered AAVs encoding C2 probes on tissue cultures after apoptosis induction. For in vivo experiments, neurotropic AAVs were intravenously injected into perinatal mice, and after 2 weeks, brain slices were collected to observe C2-SNAP expression.

**Results:**

The biophysical analysis revealed the high specificity of the C2 probe for phosphatidylserine. The fused recombinant C2 proteins were suitable for labelling phosphatidylserine on the surface of apoptotic cells in various cell lines. We engineered AAVs and validated them in organotypic brain tissue cultures for non-invasive delivery of the genetically encoded C2 probe and showed that these probes were expressed in the brain in vivo after intravenous AAV delivery to mice.

**Conclusions:**

We have demonstrated that the developed genetically encoded PS biosensor can be utilised in a variety of assays as a two-component system of C2 and C2m2 fusion proteins. This system allows for precise quantification and PS visualisation at directly specified threshold levels, enabling the evaluation of PS exposure in both physiological and cell death processes.

**Supplementary Information:**

The online version contains supplementary material available at 10.1186/s11658-023-00472-7.

## Background

Phosphatidylserine (PS) is a negatively charged lipid, which constitutes a relatively minor component of most biological membranes, accounting for only about 3–10 mol% of total phospholipids in mammalian cells [1]. However, the physiological importance of PS outweighs its low abundance. Rather than being evenly distributed, PS is preferentially located in the inner leaflet of the plasma membrane under normal conditions [2]. It is widely recognized that the disruption of this asymmetry, which leads to the exposure of phosphatidylserine on the cell surface, plays a fundamental role in apoptosis and the clearance of apoptotic cells [3]. Nevertheless, other cellular processes also necessitate the externalisation of phosphatidylserine. Specific translocation of PS is crucial for blood coagulation, bone mineralization, myoblast fusion, fertilisation, rod cell shedding, and, as recently demonstrated, in synaptic pruning [4].

Due to the contribution of PS to widespread cellular functions, an efficient PS probe is needed to study the dynamics of these processes. Currently, a few commercial PS labelling tools are available. The most commonly used label for PS is fluorescently tagged annexin A5 that can successfully detect and quantify cell death [5] or platelet activation [6] and has been used to study the activity of procoagulants [7]. However, annexin A5 requires a relatively high concentration of calcium ions for its binding (1–3 mM) [8], thus preventing the detection of internal PS in live cells or its application for in vivo assays [9]. Furthermore, it has been reported that annexin A5 has limited specificity for PS, as it binds to the phosphatidylethanolamine, the second most abundant phospholipid in eukaryotic cell membranes [10]. Therefore, alternative annexins have been explored as potential tools for more specific PS detection. Modified annexin B12 has been fused to polarity-sensitive fluorophores to develop the pSIVA probe that exhibits a significant increase in fluorescence upon binding to the membrane and displays low fluorescence when not bound [11]. However, like annexin A5, this probe also requires calcium (~ 1–2 mM) and has a limited signal-to-noise ratio for in vivo applications [12]. Alternatively, PS can be visualised by PS-specific antibodies [13]; however, antibodies exhibit cross-reactivity with other phospholipids, diminishing the specificity of such labelling techniques [14]. Small organic compounds have also been developed for PS labelling and include TNBS (2,4,6-trinitrobenzenesulfonate) [15] or Zn-DPA (bis(zinc-dipicolylamine)) and Zn-DPA-based fluorescent imaging reagent PSVue® [16]. Such compounds can detect PS on apoptotic cells in vitro when used either directly or delivered by liposomes [17]. However, these tools have significant limitations. TNBS is not suitable for live cell imaging [18] and shows limited selectivity to PS due to the reaction with phosphatidylethanolamine and other amines [19]. Meanwhile, PSVue demonstrates a significant background signal [20]. Finally, the distribution and dynamics of PS can be visualised by fluorescent PS analogues, such as aromatic fluorescent compound 7-nitro-2-1,3-benzoxadiazol-4-yl (NBD) attached to the acyl chain [21]. Unfortunately, PS analogues photobleach extremely rapidly [22] and can cause membrane distortion, thus modifying the behaviour of the lipid bilayer [21].

The need to visualise the exposure of PS on the membrane of living cells in culture or in vivo, has promoted the development of genetically encoded biosensors. These biosensors are typically based on the PS-binding domains of various PS-recognizing proteins. For instance, milk fat globule-epidermal growth factor-factor 8 (MFG-E8), also known as lactadherin, is an opsonin expressed by a variety of cells, including both professional and non-professional phagocytes [23]. MFG-E8 consists of four functional domains: two epidermal growth factor domains (EGF1 and EGF2), as well as C1 and C2 domains like those found in coagulation factors V and VIII [24]. MFG-E8 tightly binds to the exposed PS through its C2 domain in a calcium-independent manner [25, 26]. Therefore, the C2 domain of MFG-E8 can be used to develop a sensitive probe for PS exposed on cell surfaces, overcoming several challenges presented by the aforementioned tools. It has been demonstrated that the C2 domain binds to PS more sensitively and efficiently than annexin A5, enabling the detection of lower levels of PS and cell apoptosis at earlier stages compared to other available tools [9]. The microinjection of a plasmid encoding MFG-E8 fused to red fluorescent protein mCherry into *C. elegans* has allowed visualisation of PS in vivo on necrotic neurons after dysregulated Ca^2+^ influx, which induced excitotoxicity [27]. PS labelling on living cells has also been achieved using the C2 domain alone [28, 29]. The fusion of the C2 domain with green fluorescent protein enables the monitoring of the endogenous distribution of PS in intact *S. cerevisiae* and various mammalian cells [28, 29]. However, this approach is not suitable for labelling PS on the external surface of the cells and thus cannot be used to investigate the mechanisms and the role of PS exposure. Therefore, our aim was to develop a genetically encoded probe for sensitive and selective PS labelling in both in vitro cell cultures and ex vivo and in vivo tissues. We utilised the C2 domain of MFG-E8 for sensitive and selective PS binding and fused it to either red fluorescent protein mKate [30] or the self-labelling enzyme SNAP-tag® [31]. We characterised our PS probe on artificial hybrid bilayer lipid membranes and in different mammalian cell cultures, demonstrating its applicability as a biomarker for sensitive and selective PS labelling in tissue after non-invasive AAV delivery.

## Materials and methods

### The modelling of the structure of fused recombinant proteins

The structures of fused recombinant C2-mKate and C2-SNAP proteins were predicted using de novo modelling tools Robetta [32], Raptor-X [33] and artificial intelligence tool AlphaFold 2 [34, 35]. The quality of the obtained models was assessed using VoroMQA [36], ProSA [37], QMEANDisCo [38], and ProQ2 [39] values and the optimal model for each protein was chosen (Additional file 1: Tables S1, S2). The selected protein models for C2-mKate and C2-SNAP were aligned to PDB structures of individual domains (C2—PDB ID:2L9L [26], mKate—PDB ID:3BXC [40], SNAP-tag—PDB ID:3KZZ) using the PyMOL program.

### Plasmids for fused protein expression

All plasmids in this study were obtained using traditional molecular cloning techniques. For recombinant protein expression, fused proteins were cloned into pET-21a(+) plasmid. The open reading frame of the C2 domain was amplified using mouse tissue DNA as a template and Thermo Fisher Scientific Phusion Flash High-Fidelity PCR Master Mix, following manufacturer’s recommendations. The pET-C2 plasmid was obtained by ligating NdeI and HindIII restriction fragments of pET21a(+) vector and C2 PCR product. For protein fusion, flexible peptide linker was used [41]. The SNAP-tag gene was amplified from a plasmid kindly gifted by Heppenstall [42], and the mKate gene was amplified from pAAV-GFAP-mKate plasmid (Addgene #99129, RRID:Addgene_99129) with primers that inserted XhoI and HindIII restriction sites. The SNAP-tag or mKate amplicons were ligated into the pET-21a(+) plasmid after restriction with HindIII and XhoI. The sequences of all constructs are provided in Additional file 2.

### The mutagenesis of C2 domain

To obtain the inactive C2 domain, substitutions were made at Lys24 and Lys45, replacing them with asparagines. Site-directed mutagenesis was performed for the Lys24 substitution using specific primers (F: 5′-CAGCTACAATACATGGAACC-3′ and R: 5′-CTGGAGGCTGACATCTGGCT-3′) for PCR amplification with Thermo Fisher Scientific Phusion Flash High-Fidelity PCR Master Mix. Similarly, site-directed mutagenesis for the Lys45 substitution was carried out using specific primers F: 5′-TCAGGGCAATATCAATGCCT-3′ and R: 5′-TTATCCAGCCTTCCCAAGTG-3′. The resulting pET21a-C2m2 plasmid was used to construct pET21a-C2m2-SNAP and pET21a-C2m2-mKate plasmids, following the procedure described above.

### pAAV plasmid for virus production

The plasmids for virus production were designed to carry astrocyte specific promoter GFAP sequence from pAAV-GFAP-mKate plasmid (Addgene plasmid #99129, RRID:Addgene_99129). Signal peptide sequence (UniProtKB/Swiss-Prot Accession Number: P21956) [43] was introduced to ensure the secretion of fused proteins.

### Protein expression and purification

The recombinant pET21a-C2-mKate, pET21a-C2m2-mKate, pET21a-C2-SNAP or pET21a-C2m2-SNAP expression plasmids were transformed into *E. coli* BL21(DE3), HMS174(DE3), C43(DE3)pLysS, NovaBlue(DE3), Rosetta-gami 2(DE3), or ArcticExpress (DE3) competent cells. Transformants were selected by plating cells on agar plates containing ampicillin (100 μg/ml). The expression of recombinant proteins was induced by either 0.5 mM or 1.0 mM (2*R*,3*R*,4*S*,5*R*,6*S*)-2-(hydroxymethyl)-6-(propan-2-ylsulfanyl)oxane-3,4,5-triol (IPTG) at different temperatures (13 °C, 20 °C, 25 °C, 16 °C) with continuous shaking. After 16 h, cells were harvested by centrifugation at 3000×*g* for 20 min. at 4 °C. The pellet was sonicated using BANDELIN SONOPULS mini20 with VS70T probe for 5 min. at an amplitude setting of 50%. Sonication was performed for 15 s with 15 s intervals to cool down the specimen. Cell debris was centrifuged at 3000×*g* for 25 min. at 4 °C. The solubility of recombinant proteins was assessed by 14% SDS-PAGE. The recombinant proteins from the supernatant of ArcticExpress (DE3) biomass induced by 1 mM IPTG at 16 °C were purified by immobilized chelate affinity chromatography on HisPur™ Ni–NTA (Thermo Fisher Scientific) column using ÄKTA™ avant 25 chromatography system (Cytiva). After loading the sample, the system was washed with NPI-10 buffer (50 mM Na_2_HPO_4_, 300 mM NaCl, 10 mM imidazole). The proteins with His-tag were eluted with NPI-400 buffer containing 400 mM imidazole. Elution fractions were loaded on 14% SDS-PAGE to select those containing purified fused recombinant proteins. Chosen fractions were concentrated using Amicon® Ultra-4 Centrifugal Filter Units 10 kDa (Millipore) in a buffer containing 20 mM Na_2_HPO_4_, 20 mM NaCl, pH 6.5. The concentration of purified proteins was determined using NanoDrop™ 2000 (Thermo Fisher Scientific).

### Preparation of multilamellar vesicles (MLV)

Vesicle suspensions were prepared using defined mixtures of 1,2-dioleoyl-sn-glycero-3-phosphocholine (DOPC), cholesterol (CHOL), 1,2-dioleoyl-sn-glycero-3-phosphoethanolamine (DOPE) and 1,2-dioleoyl-sn-glycero-3-phospho-l-serine (DOPS) from Avanti Polar Lipids, Inc., USA. The phospholipids were dissolved in chloroform at a concentration of 10 mM to obtain stock solutions that were used for final lipid mixtures. Three different vesicle compositions were used, each with different molar ratios of lipids: neutral membrane (60% DOPC and 40% CHOL), a membrane containing PS (45% DOPC and 30% CHOL with 25% DOPS), and a membrane containing PE (45% DOPC and 30% CHOL with 25% DOPE). The lipid mixtures were dried for 30 min. under a continuous flow of nitrogen to evaporate chloroform, resulting in the formation of a thin, uniform lipid film. The resulting lipid film was resuspended in PBS at a total lipid concentration of 1 mM using mixing and vortexing until the lipid film on the vial walls dispersed and the solution became turbid.

### Substrate preparation for surface plasmon resonance

BK7 glass slides (25 mm diameter, 1 mm thickness, Methorm AutoLab, The Netherlands) were prepared for surface plasmon resonance (SPR) as previously described [44]. In brief, glass slides were coated with the Cr (2 nm) and Au (50–90 nm) films using the PVD 75 magnetron sputtering system (Kurt J. Lesker Company, USA). Immediately after the sputtering, the slides were transferred into the self-assembled monolayer (SAM) preparation solution containing 0.1 mM octadecanethiol (ODT) dissolved in ethanol. Incubations were carried out for 3–4 h at RT. Then, the slides were washed with ethanol and dried in a stream of nitrogen. The hybrid bilayer lipid membranes (hBLM) were formed through a fusion mechanism by exposing the samples with SAM to a 100 μl solution of multilamellar vesicle (1 mM). After incubating with the multilamellar vesicles (MLV) for 20–30 min., the vesicles were rinsed with PBS buffer, and the hBLM was formed.

### Surface plasmon resonance assay

Surface plasmon resonance (SPR) measurements were conducted using an Autolab Twingle system (Eco Chemie B.V., The Netherlands) equipped with a flow-through cell, as previously described [44]. The system automatically tracked millidegree positional changes in the reflection angle resulting from the excitation of surface plasmon resonance. The temperature was stabilised at 22 ± 0.1 °C using F34 refrigerating/heating circulator (Julabo, Germany). Prior to each MLV fusion experiment, the baseline in PBS solution was recorded.

Once an hBLM was formed and the minimum angle baseline was established, the proteins were injected into the cell. After reaching a plateau in protein binding (at approximately 20 min.), any nonspecific protein interaction was disrupted by washing with PBS. Protein binding was quantified as the change in SPR angle after wash-out. To regenerate the cell, it was washed with 1 M NaOH and then rinsed with PBS.

### Cultivation of mammalian cell lines

HEK293T (RRID:CVCL_0063) and Neuro2a (RRID:CVCL_0470) cell lines were purchased from American Type Culture Collection (ATCC). Human embryo kidney HEK293T cells were maintained in T25 flasks with Dulbecco’s Modified Eagle’s Medium (DMEM) supplemented with GlutaMAX™, 5% foetal bovine serum (FBS), 100 U/ml penicillin, 100 µg/ml streptomycin, and 1% non-essential amino acids (Thermo Fisher Scientific). Mouse neuroblastoma Neuro2a cells were cultured in Eagle’s Minimum Essential Medium (EMEM) supplemented with 10% FBS, and 100 U/ml penicillin, 100 µg/ml streptomycin. The cell cultures were maintained at 37 °C and 5% CO_2_. The cells were harvested using TrypLE™ solution. To evaluate phosphatidylserine (PS) exposure, the cells were plated at a density of 400 cells/mm^2^ onto 8-well plates (Nunc™ Lab-Tek™, Thermo Fisher Scientific) that were pre-coated with poly-l-lysine (0.001% in PBS) for at least 20 min. at RT.

### Apoptosis assay

To induce apoptosis, 24 h after plating the cells were treated with 3 µM of staurosporine (Sigma Aldrich) for 16 h. PS exposure was then detected by adding 100 µg/ml recombinant fusion proteins for 30 min. Excess protein was removed by rinsing with pre-warmed media, and then the cells were fixed with 4% paraformaldehyde (PFA) in PBS for 20 min. at RT. After rinsing with PBS, residual PFA was quenched with 30 mM glycine in PBS, and the cells were washed twice with PBS. To visualise C2-SNAP and C2m2-SNAP proteins, the cells were labelled with 3 µM of benzylguanine substrate SNAP-Surface® Alexa Fluor® 647 (NEB) for 30 min. at RT. Excess substrate was removed by washing with PBS three times and then incubating in PBS for additional 30 min. All cells were stained with DAPI (1 μg/ml) for 10 min. at RT. After the final three washes with PBS, Lab-Tek chambers were removed, and the microscopic slide was coverslipped with Mowiol (Calbiochem). The imaging was performed on the confocal Leica TCS SP8 microscope with 63×/1.4 oil immersion objective (pixel size 0.481 × 0.481 µm). A diode laser with an excitation wavelength of 405 nm was used for DAPI excitation. The white light laser was used for the excitation of PSVue (*λ* = 553 nm), AlexaFluor647 (*λ* = 647 nm), and mKate (*λ* = 589 nm). DAPI fluorescence was collected using a photomultiplier tube (PMT) detector (415–550 nm). The fluorescence of AlexaFluor647 (657–680 nm), mKate (599–750 nm), and PSVue (563–660 nm) was collected using a hybrid detector with a gating of 0.3–6 ns.

### Cell image analysis

Confocal images were analysed using the open-source CellProfiler software (RRID:SCR_007358) [45]. The integrated intensity of C2 probe-specific fluorescence was measured in each image (*n* = 10–15 images for each condition, all conditions were repeated in duplicates across three independent experiments). The intensity was estimated per cell by dividing the image fluorescence intensity by the count of nuclei in that image (Additional file 3). Fluorescence values were normalised to the highest obtained values of C2-SNAP and C2-mKate, as required. Detailed image analysis pipeline with an example is presented in Additional files 1: Fig. S1 and 3.

### Adeno-associated virus production

The adeno-associated viruses (AAVs) expressing C2 probes were produced as described previously [46]. For viral packaging, HEK293T cells were plated at a density of 6 × 10^4^ cells/cm^2^. After 24 h, the cells were transfected by three plasmids: (1) pAAV encoding the C2 probe of interest, engineered in this study as described above, (2) capsid-encoding plasmid pUCmini-iCAP-PHP.eB (a gift from Viviana Gradinaru, Addgene plasmid #103005; RRID: Addgene_103005) [47], and (3) helper plasmid XX6-80 (received from The National Gene Vector Biorepository, NGVB) at the ratio of 1:4:2, with a total of 40 μg of DNA per dish. The transfection was mediated by polyethylenimine (linear, MW 25 000, Sigma Aldrich). After 72 h and 120 h post-transfection, cell media were collected and filtered through a 0.45 μm filter to remove cell debris and sterile-filtered through 0.22 μm syringe filter. The AAV solution was concentrated by centrifugation in Amicon Ultra Centrifugal Filter Units 100 kDa (Merck Millipore) at 3000×*g* for 8 min. at RT or until the volume of the solution remaining in the top chamber of the Amicon filter device was 500–1500 μl. Then DPBS with 0.001% Pluronic F-68 (Thermo Fisher Scientific) was added to the AAV solution in Amicon filter device and centrifuged 3000×*g* for 8 min. at RT, repeating the washing three times. During the last spin 300–500 µl of AAV solution was collected. AAV titre was determined by quantitative PCR using SYBR Green, as described previously [46]. In brief, to degrade any unpacked DNA, DNAse I was added, which was then inactivated with EDTA. Viral DNA was released using proteinase K and detected by qPCR. The obtained Ct values were used to determine AAV titres.

### Animals

*Thy1*::EGFP mice (IMSR Cat# JAX:007788, RRID:IMSR_JAX:007788) were used in the heterozygous state. Animal studies were conducted in accordance with the requirements of the Directive 2010/63/EU and were approved by the Lithuanian State Food and Veterinary Service (Permit No G2-92, B6-(1.9)-2653). All mice were bred and kept at the animal facility of the Life Sciences Center of Vilnius University.

### Organotypic hippocampus slice culture preparation

Organotypic hippocampal slice cultures (OHSC) were prepared using the interface method, as previously described [48, 49]. Briefly, 3-day-old pups of *Thy1*::EGFP mice were decapitated after cervical dislocation. The isolated brain was placed in a Petri dish filled with an ice-cold dissection medium (100 U/ml penicillin, 100 µg/ml streptomycin, 15 mM HEPES, 0.5% glucose in HBSS). The removed hippocampi were sliced at a thickness of 300 μm using a McIlwain tissue chopper. The intact slices were carefully planted on prepared 0.4 μm 30 mm diameter cell culture inserts with PTFE membrane (Merck Millipore) with maintaining medium (25% 1× BME, 25% horse serum, 5% 10× MEM, 100 U/ml penicillin, 100 µg/ml streptomycin, 2 mM GlutaMAX, 0.65% glucose, 9 mM sodium bicarbonate in ddH_2_0) and maintained in 5% CO_2_ incubator at 37 °C. The medium was changed 24 h after the plating and every 2–3 days during culture maintenance.

### PS labelling in AAV-transduced OHSC

OHSC were transduced with 6.7 × 10^10^ vg of AAV-C2-mKate, AAV-C2m2-mKate, AAV-C2-SNAP, or AAV-C2m2-SNAP at 5 days in vitro (DIV5). The medium was changed 2 days after treatment and then every 2–3 days. The selected treatment was performed 9 days after the transduction, at DIV14. To inhibit fused protein secretion, OHSC were treated with 10 µg/ml of brefeldin A (BFA) (Thermo Fisher Scientific) for 5 h. To induce apoptosis, OHSC were treated with 100 μM of staurosporine (Sigma Aldrich) for 16 h. After either treatment, slice cultures were washed with prewarmed media and fixed in 4% PFA in PBS for 30 min. at RT. Next, slices were rinsed once with PBS and quenched with 30 mM glycine/PBS. OHSC expressing C2-SNAP and C2m2-SNAP were labelled with 3 μM benzylguanine substrate SNAP-Surface® Alexa Fluor ® 647 for 2 h at RT. Nuclei were counterstained with DAPI (1 μg/ml) for 15 min. at RT. Both apoptosis and protein localization experiments were performed in three independent cultures containing duplicates for each experimental condition.

### OHSC immunofluorescence labelling

To label different cell types in BFA-treated OHSC, fixed slices were permeabilized and blocked in 0.4% Triton-X/PBS with 20% normal goat serum (NGS) (Abcam). They were then incubated for 16 h on a shaker at 4 °C. Afterwards, the slices were incubated with primary antibodies (polyclonal rabbit anti-Iba1or polyclonal chicken anti-GFAP (Table 1), in 0.1% Triton-X with 5% NGS for 24 h on a shaker at 4 °C. Following three washes with PBS, the slices were incubated with secondary antibodies (polyclonal goat anti-rabbit Alexa Fluor Plus 594, or polyclonal goat anti-chicken Alexa Fluor Plus 594 (Table 1), respectively) in PBS with 5% NGS for 4 h on a shaker at RT. The nuclei were counterstained with DAPI (1 μg/ml) for 15 min. at RT. After three more washes with PBS, the slices were mounted onto the microscope slides using Mowiol (Calbiochem). Images were acquired using Leica TCS SP8 confocal microscope with either 63×/1.4 oil immersion objective (pixel size 0.18 × 0.18 µm) for protein secretion experiments or dry 20×/0.75 objective (pixel size 1.137 × 1.137 µm) for apoptosis experiments. For DAPI excitation, a diode laser with a wavelength of 405 nm was used. A white light laser was used for the excitation of AlexaFluor647 (*λ* = 647 nm), mKate (*λ* = 589 nm), EGFP (*λ* = 488 nm), and AlexaFluor594 plus (*λ* = 594 nm). DAPI fluorescence was collected using a photomultiplier tube (PMT) detector (415–550 nm). The fluorescence of AlexaFluor647 (657–680 nm), mKate (599–750 nm), and AlexaFluor594 plus (604–650 nm) was collected using a hybrid detector under gating of 0.3–6 ns.

**Table 1:** Primary and secondary antibodies used in this study

Antibody	Source	Final concentration (µg/ml)	Host	Research resource identifiers	Lot number
Anti-Iba1	FUJIFILM Wako Chemicals	1	Rabbit	RRID:AB_839504	LEK0542
Anti-GFAP	Abcam	36.8	Chicken	RRID:AB_1975558	GR3393187-1
Anti-rabbit Alexa Fluor Plus 594	Invitrogen	4	Goat	RRID:AB_2762824	2307236
Anti-chicken Alexa Fluor Plus 594	Invitrogen	4	Goat	RRID:AB_2762829	XE349349

### OHSC image analysis

Image analysis was performed using ImageJ software (RRID:SCR_003070) [50]. The accumulation of C2-SNAP, C2m2-SNAP, C2-mKate or C2m2-mKate fusion proteins in astrocytes was quantified by assessing SNAP-specific fluorescence within GFAP-specific signal. For both SNAP and GFAP channels, the background was removed using the *Subtract background* function (rolling ball radius 50 pixels). Images were smoothed with *Gaussian Blur* filter (sigma = 2) and thresholded using the Otsu algorithm with the *Auto-Threshold* function to obtain masks for further measurements*.* The image of SNAP signal within the astrocytes was obtained using the *Process-Image Calculator* function with the selected operation as “AND,” and the area of both GFAP and SNAP within GFAP was determined using *Measure* function. Image processing example is shown in Additional file 1: Fig. S2.

### AAV transduction in vivo

Study design: mice injected with AAV-C2-SNAP were compared to non-injected littermate control using a single mouse as an experimental unit. Sample size: five mice were injected with AAV-C2-SNAP, one mouse was used as non-injected control. In total, six mice were used. The number of animals was chosen to be sufficient for qualitative analysis. Inclusion and exclusion criteria: no animals were excluded during the experiment. Randomization: mice were allocated to the injection and control groups randomly. The cofounders were controlled by comparing littermate injected and control mice. Blinding: blinding was not applied. Outcome measures: C2 labelling was qualitatively assessed by imaging fluorescent signal in the brain samples from AAV-C2-SNAP and control animals. Statistical methods: only qualitative analysis was performed. Experimental animals: 3-day-old (P3) homozygous *Thy1*::EGFP mice. Experimental procedures: P3 mice were injected into the facial vein with 1 × 10^11^ vg of AAV-C2-SNAP in 0.9% saline, in a total volume of 20 µl, using an insulin syringe with a 29G needle. After 2 weeks (at P17), mice were transcardially perfused with 0.9% saline for 2 min., followed by 4% PFA for 5 min. The brains were post-fixed for 24 h and then sectioned at 50 µm using a 5100mz vibratome (Campden Instruments). Free-floating brain sections were labelled with 3 µM SNAP-Surface® Alexa Fluor® 647, counterstained with DAPI (1 μg/ml) for 15 min. and washed with PBS. Slices were mounted in Mowiol (Calbiochem), and images were acquired using a Leica TCS SP8 confocal microscope with a 63×/1.4 oil immersion objective (pixel size 0.18 × 0.18 µm).

### Statistical analysis

Neuro2a and HEK293T cell culture, as well as OHSC experiments, were replicated in at least three independent cultures. For SPR experiments, at least six independent measurements were completed. Data presented were expressed as means ± standard error of the mean. Fold change graphs were expressed as geometric means ± 95% confidence intervals. Statistical analysis was performed using GraphPad Prism (version 9.5.1 for Windows, GraphPad Software, San Diego, California USA). The normality of the data was verified by the Shapiro–Wilk test. The means were compared by one-way ANOVA and *post-hoc* Tukey test. The geometrical means of fold change were compared by Kruskal–Wallis one-way analysis of variance and *post-hoc* Dunn’s test. *p* values < 0.05 were considered as significant.

## Results

### Protein modelling suggests structural integrity of fused proteins

Due to the lack of specific PS probes for in vivo applications, our aim was to develop a genetically encoded tool. To overcome the drawbacks of currently available PS labelling tools discussed above, we chose the C2 domain of the MFG-E8 protein as the basis for PS recognizing tool. We fused it with proteinaceous tags for visualisation: either a fluorescent mKate protein [30] or a self-labelling enzymatic SNAP-tag [51]. Fluorescent proteins allow direct imaging of a bound probe without any additional staining procedures [52]. However, fluorescent proteins, particularly those excited by longer wavelengths, have significantly lower brightness than organic dyes. Therefore, we also employed SNAP-tag, which is visualised by highly specific and irreversible attachment of its synthetic benzylguanine (BG) derivative ligands to covalently bind chosen fluorophore [51]. To ensure the functionality of both fused proteins, we chose to space them with a linker composed of threonine, serine, glycine, which are the preferable amino acids for natural linkers [53, 54].

**Fig. 1:**
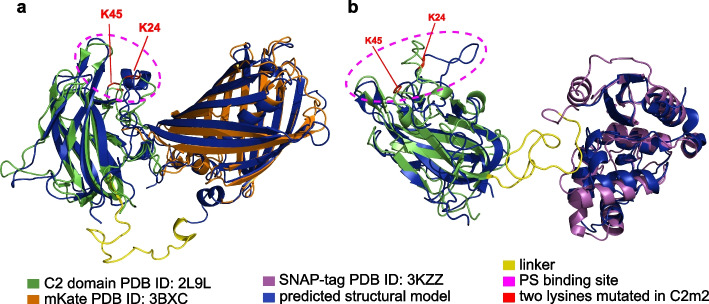
Structural models of recombinant C2 fusion proteins. **a** C2-mKate aligned to experimentally defined structures of C2 domain (PDB ID: 2L9L) and mKate (PDB ID: 3BXC). **b** C2-SNAP aligned to experimentally defined structures of C2 domain and SNAP-tag (PDB ID: 3KZZ). Purple dashed circle marks putative site of C2 and PS interaction, including critical amino acids K24 and K45 in red

To assess whether the C2 domain and the fluorescence tag can be compatible after the fusion, we created structural models of C2-mKate and C2-SNAP using de novo modelling tools. To identify the most energetically and spatially favourable model we used VoroMQA, ProSA, QMEANDisCo and ProQ2 quality assessment tools (Additional file 2: Tables S1, S2). We then aligned the preferable models for C2-mKate and C2-SNAP fusion proteins to the structures of C2, mKate and SNAP-tag from the Protein Data Bank (PDB) [55] to compare the models of the fusion proteins to the experimentally defined structures of each individual domain (Fig. 1). We demonstrated that the C2 domain in both fused proteins retained the same fundamental structural motifs as in PDB experimentally defined structures, namely a beta barrel core with seven loops. Importantly, the loops 1–3 that constitute the binding site for the PS [26] were arranged as in native C2 (Fig. 1), suggesting that fused proteins should retain the affinity to the PS. Furthermore, the key lysine residues (K24 and K45) that are essential for PS binding [26] were in similar positions in the model of fused proteins as in the PDB structures (Fig. 1a, b), further suggesting that the fusion did not affect the structure of the active site of C2 domain.

The structural motifs of fluorescence tags were also maintained in fused proteins. The structure of mKate in a fused model overlapped with the motifs of the beta barrel with an alpha spiral inside that are required for the fluorescence (Fig. 1a). The SNAP-tag in the fused protein was mostly composed of alpha spirals, closely resembling the PDB structure (Fig. 1b). The introduced linker had sufficient length to alleviate any tensions between the fused domains (Fig. 1). The models predicted by *AlphaFold 2* (Additional file 1: Fig. S3) were similar to the structures created by de novo modelling tools. The PS binding site in the C2 domain remained intact. In particular, loops 1–3 and two lysines were arranged close to one another suggesting retained functionality of the C2 domain. The spatial arrangement of the linker in *AlphaFold 2* predictions was different, but this did not affect predicted structures of functional domains. These results suggested that the fused proteins should retain their functional structures in a recombinant protein to be expressed in cells.

### C2-mKate and C2-SNAP purification

To experimentally define the interaction of designed protein probes with PS, we first expressed recombinant proteins in bacteria using pET system plasmids (Additional file 2: Fig. S4a) and purified them chromatographically. To evaluate the specificity of the C2 probe, we also developed mutated versions (C2m2) of the designed fusion probes. In C2m2, the codons that specify previously identified key amino acids K24 and K45 [26] were substituted with asparagine codons (Additional file 1: Fig. S4b). All of the proteins were fused with a six histidine residues tag for immobilized metal affinity chromatography.

To purify the fusion proteins, we tested different *E. coli* strains BL21(DE3), Rosetta-gami 2(DE3), HMS174(DE3), NovaBlue(DE3), C43(DE3)pLysS, ArcticExpress(DE3) and different induction conditions (temperature, IPTG concentration) as well as cell lysis conditions (NaCl concentration and pH of cell sonication buffer) to obtain the highest content of soluble recombinant protein (Additional file 1: Table S3). In all the strains, except for ArcticExpress(DE3), recombinant protein precipitated in the pellet rather than remaining in the soluble fraction, independently of other tested parameters (Additional file 1: Table S3). This precipitation rendered these strains unsuitable for the purification of recombinant fusion protein. Although we did not explore the reasons for the precipitation further, it could potentially be caused by the lack of post-translational modifications of the C2 domain, which may be essential for its structural integrity [23]. Recombinant fusion proteins were successfully expressed in the ArcticExpress(DE3) strain after 1 mM IPTG induction at 16 °C using 300 mM NaCl and pH 8 cell lysis buffer (Additional file 2: Table S3). It is known that the ArcticExpress(DE3) strain produces exogenous chaperones Cpn10 and Cpn60, which facilitate protein folding [56]. These chaperones are active at lower temperatures, which also slow down the expression of a recombinant protein, further contributing to correct protein folding [56]. Using ArcticExpress(DE3) bacteria, we were able to purify C2, C2-mKate, C2m2-mKate, C2-SNAP and C2m2-SNAP recombinant proteins, which were further used to characterize C2 fusion proteins as PS probes.

### PS affinity and specificity of C2-mKate and C2-SNAP probes

**Fig. 2:**
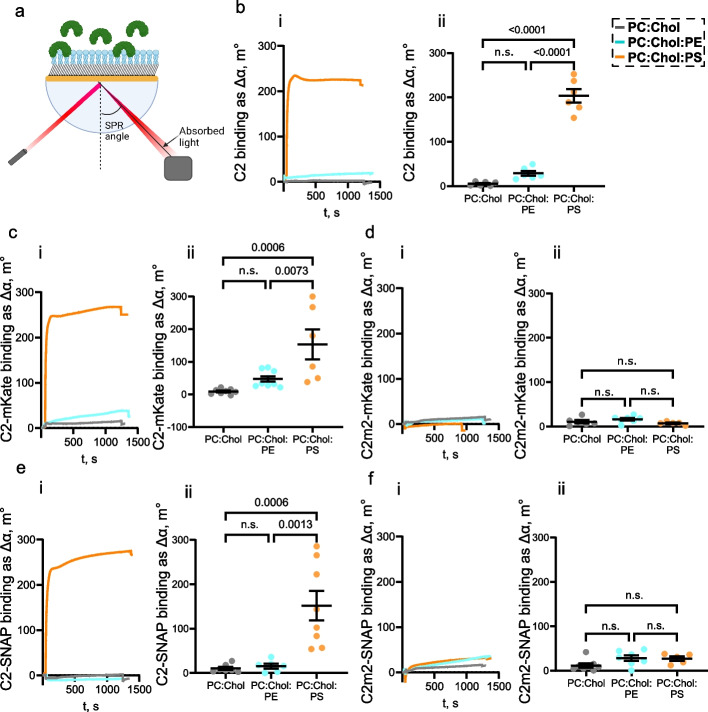
Surface plasmon resonance (SPR) revealed the specificity of C2 fusion proteins to PS in artificial hybrid bilayer lipid membranes (hBLM). **a** Schematic representation of SPR: the monochromatic light is reflected on a gold surface. At a certain angle, where the surface plasmons are excited, the reflected light has a minimum, which is continuously measured. The angle changes upon protein binding on the lipid surface. **b**–**f**, **i** Representative SPR sensorgrams of C2, C2-mKate, C2m2-mKate, C2-SNAP, C2m2-SNAP binding to the membranes of different composition: neutral hBLM composed of cholesterol and phosphatidylcholine (PC:Chol), phosphatidylethanolamine-enriched hBLM (PC:Chol:PE), phosphatidylserine-enriched hBLM (PC:Chol:PS). **b**–**f**, **ii** Quantification of C2 fusion protein binding after wash-out. Data are presented as means ± standard error of the mean (*n* = 6–8 independent measurements). Means were compared by one-way ANOVA and *post-hoc* Tukey test. *p* values < 0.05 were considered as significant

We measured C2 probe binding to different composition hybrid bilayer lipid membranes (hBLM) using SPR. An increase in mass at the SPR sensor surface, caused by the binding of proteins to artificial hybrid phospholipid membranes, results in a change in the local refractive index. This change gives rise to an SPR response, which is observed as a shift in the SPR angle. Figure 2 displays representative SPR sensorgrams obtained on hBLM exposed to purified recombinant C2 domain, C2-mKate, and C2-SNAP proteins. To assess the affinity and specificity of the purified recombinant C2-mKate and C2-SNAP proteins to the PS, we measured the SPR angle shift after the binding of C2 and C2 fusion proteins on hBLM: (1) neutral (PC:Chol) hBLM, (2) PE-enriched (PC:Chol:PE) hBLM or (3) PS-enriched (PC:Chol:PS) hBLM (Fig. 2). The binding of the protein on the lipid surface was indicated by an increase in the SPR angle (Fig. 2a). As expected, the C2 domain bound only to PS and not to Chol, PC or PE (Fig. 2b, c). During the C2 binding assay on PS-enriched membranes, the increase of SPR angle remained significant even after PBS wash-out, indicating a high affinity of the C2 domain for PS (Fig. 2b). The binding of the C2 domain to PS was significantly stronger than to neutral and PE-enriched membranes, and PE did not significantly increase C2 binding to the membranes, indicating a high specificity of the C2 domain to PS (Fig. 2c).

Next, we investigated whether the C2 domain fused to fluorescence tags retained its affinity and specificity for PS. The binding traces of C2-mKate and C2-SNAP proteins reflected those observed for the individual C2 domain (Fig. 2d, h). Strong and specific binding of C2-mKate and C2-SNAP proteins to PS-enriched membranes was observed through SPR measurements, in comparison to the neutral or PE-enriched membranes (Fig. 2e, i).

Previous studies have shown that mutations of key amino acids K24 and K45 inhibit C2 binding to the PS exposed on the cell surface [29]. To assess the specificity of the interaction between C2 fusion proteins and PS, we evaluated the binding of mutant C2 domain probes (C2m2) to different hBLMs (Fig. 2f, j). We found no significant differences in the binding of C2m2 fusion proteins to membranes with different phospholipid composition (Fig. 2g, k), indicating that mutated C2 probe counterparts can be used to define background binding in any experimental system. Therefore, we were able to conclude that fusing the C2 domain to mKate or SNAP-tag did not affect the binding of the C2 domain to PS, while mutated C2m2 fusion proteins can be used to directly evaluate any background signal and nonspecific binding of the designed PS probes in further experiments.

### C2-mKate and C2-SNAP label apoptotic cells in vitro

**Fig. 3:**
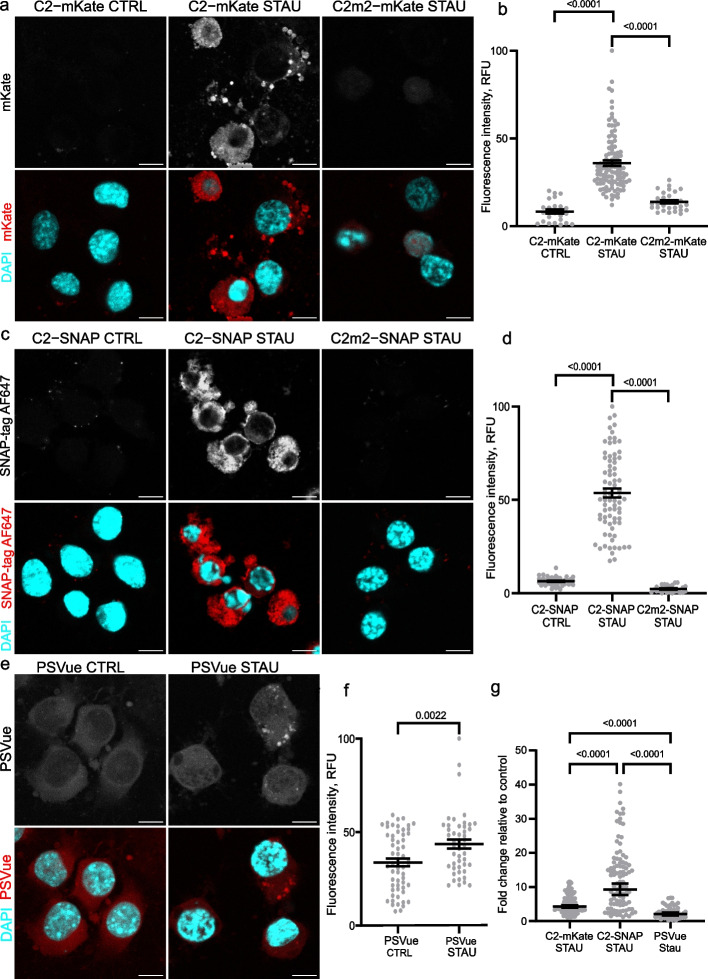
C2 fusion proteins label apoptotic Neuro2a cells in vitro. **a**, **c** Confocal images of control (CTRL) and apoptotic (STAU, treated with 3 µM staurosporine) Neuro2a cells labelled with either C2-mKate or C2-SNAP or their mutated counterparts C2m2-mKate and C2m2-SNAP; scale bar 10 µm. **b**, **d** Quantification of mKate or SNAP-tag fluorescence intensity on Neuro2a cells with or without staurosporine treatment. **e** Confocal images of control (CTRL) or apoptotic (STAU, treated with 3 µM staurosporine) Neuro2a cells labelled with PSVue. **f** Quantification of PSVue fluorescence intensity on Neuro2a cells with or without staurosporine treatment. **g** Comparative signal strength of mKate, SNAP and PSVue on apoptotic Neuro2 cells. Data presented as means ± standard error of the mean (**b**, **d**, **f**) or geometrical means ± 95% confidence intervals (**g**) (*n* = 20–30 images per biological replicate, 3 independent biological replicates). Means were compared by one-way ANOVA and *post-hoc* Tukey test (**b**, **d**, **f**) or by Kruskal–Wallis one-way analysis of variance and *post-hoc* Dunn’s test (**g**). *p* values < 0.05 were considered as significant. *RFU* relative fluorescence units

To test whether PS-specific C2 fusion proteins are suitable for detecting the exposure of PS on the plasma membrane of cells in vitro, we induced apoptosis in the culture of two different cell lines: mouse neuroblastoma Neuro2a and human embryonic kidney HEK293T cells. After staurosporine treatment, apoptotic cells were labelled with either C2-SNAP or C2-mKate (Fig. 3, Additional file 1: Fig. S5). In both cell lines, C2 probes did not bind to non-apoptotic cells, while on apoptotic cells both C2-mKate and C2-SNAP decorated the cell membrane, revealing the cell contour. In contrast, mutated C2m2-mKate and C2m2-SNAP probes did not label apoptotic cells (Fig. 3, Additional file 1:Fig. S5), indicating that the binding of C2 probes on apoptotic cells was PS-dependent.

Next, we compared the sensitivity of the designed C2 probes to one of PS labelling tools that can be used for both in vitro and in vivo labelling—the organic label zinc dipicolamine known as PSVue [20, 57]. In contrast to the C2 probes, PSVue labelling exhibited a high background signal on non-apoptotic cells (Fig. 3e, f, Additional file 1: Fig. S5e, f). PSVue labelled apoptotic cells with an intensity above the background, but the contour of cell surface was not observed (Fig. 3e, Additional file 1: Fig. S5e). Both C2-mKate and C2-SNAP probes had significantly higher signal-to-noise ratio compared to PSVue, whose signal on apoptotic cells was only 15% higher than that on non-apoptotic cells (Fig. 3f). Meanwhile, C2-SNAP demonstrated more than a 22-fold increase in fluorescence on apoptotic Neuro2a cells (Fig. 3d). C2-mKate signal on apoptotic Neuro2a cells increased about four-fold (Fig. 3b). C2-SNAP exhibited significantly stronger signal than C2-mKate, possibly due to favourable properties of the organic fluorophore used in SNAP-tag labelling: lower photobleaching and higher stability compared to autofluorescent proteins [52]. The in vitro experiments demonstrated that C2 fusion probes are specific and sensitive labelling tools for PS exposure on the external surface of cell plasma membrane.

### Expression of genetically encoded C2 probes in tissue culture

The investigation of PS exposure in 3D cellular models, tissue, or explant cultures poses further difficulties due to the limited permeability of externally applied PS probes. To overcome this limitation, we developed adeno-associated viral (AAV) vectors that genetically encode C2 probes and deliver them to the tissue without direct damage to the tissue of interest (Additional file 2, Additional file 1: Fig. S4c, d). We used glial fibrillary acidic protein (GFAP) promoter to target C2 expression to astrocytes [58]. To ensure the release of C2 fusion proteins into the extracellular space for the labelling of exposed PS on the external membrane surface of the cells, we introduced a secretory signal peptide [43]. Finally, for packaging, we used the AAV-PHP.eB capsid, which has been demonstrated to efficiently transduce the cells in the central nervous system after non-invasive delivery [47].

**Fig. 4:**
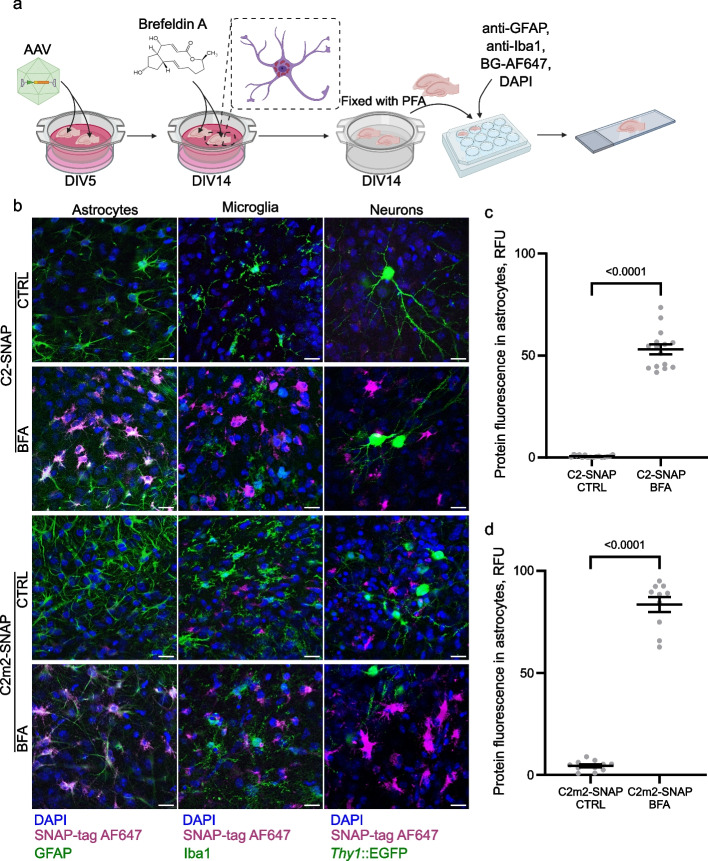
The expression of C2 fusion proteins in ex vivo organotypic hippocampal slices after AAV delivery. **a** OHSC were transduced with AAV carrying either C2-SNAP or C2m2-SNAP at 5 days in vitro (5DIV) and maintained for 9 days for protein expression. The secretion of recombinant proteins was inhibited by 10 µg/ml brefeldin A (BFA) for 5 h. Fixed control (CTRL) and inhibited (BFA) slices were labelled by immunohistochemistry and SNAP-tag substrate for fluorescent imaging. Created by Biorender.com. **b** Confocal images of OHSC transduced with either AAV-C2-SNAP or AAV-C2m2-SNAP. Cell nuclei were stained with DAPI, fused SNAP-tag was labelled with AlexaFluor647 (AF647). Astrocytes, microglia and neurons were labelled with GFAP, Iba1 antibodies, or expressed EGFP, respectively. Scale bar 20 µm. **c**, **d** Quantification of C2-SNAP and C2m2-SNAP fluorescence within astrocytes with or without BFA treatment. Data presented as means ± standard error of the mean (*n* = 10–15 images per replicate, two independent replicates). Means were compared by one-way ANOVA and *post-hoc* Tukey test. *p* values < 0.05 were considered as significant. *RFU* relative fluorescence units

To test genetically encoded C2 probes, we used these engineered AAVs in organotypic hippocampal slice cultures—a well-established 3D tissue model that closely resemble the development, cell physiology and intercellular interactions in the brain tissue [59]. By design, AAV-delivered C2 fusion proteins were secreted. Therefore, to assess their expression in the ex vivo tissue, we used brefeldin A (BFA) to inhibit protein secretion and accumulate the recombinant protein in the Golgi–endoplasmic reticulum compartment of expressing cells [60]. By using immunolabelling we identified the major nervous tissue cell types to determine the cells expressing C2 probes in a tissue culture. OHSC slices were transduced with engineered AAVs at the 5th day in vitro (DIV5) (Fig. 4a). At DIV14, tissue cultures were treated with BFA for 5 h to inhibit protein secretion. We found that after BFA treatment C2-SNAP, C2m2-SNAP, C2-mKate, and C2m2-mKate proteins accumulated in cells in organotypic slices (Fig. 4b, Additional file 1: Figs. S6a–S9). In contrast, in slices without treatment to inhibit protein trafficking, no accumulation of C2 fusion probes was observed, indicative of effective protein secretion (Fig. 4b, Additional file 1: Fig. S6). To determine what cells expressed C2 probes, we colocalized C2 fusion proteins with astrocytic (GFAP), microglial (Iba1) and neuronal (*Thy1*::GFP) markers (Additional file 1: Figs. S7–S9). C2 was only found in astrocytes, but not microglia or neurons (Fig. 4b–d, Additional file 1: Fig. S6). These results revealed that AAV-delivered C2 probes are selectively expressed in astrocytes and are efficiently secreted into the extracellular space under normal conditions in 3D brain tissue cultures.

### AAV-encoded C2 probes label apoptotic cells in tissue culture

**Fig. 5:**
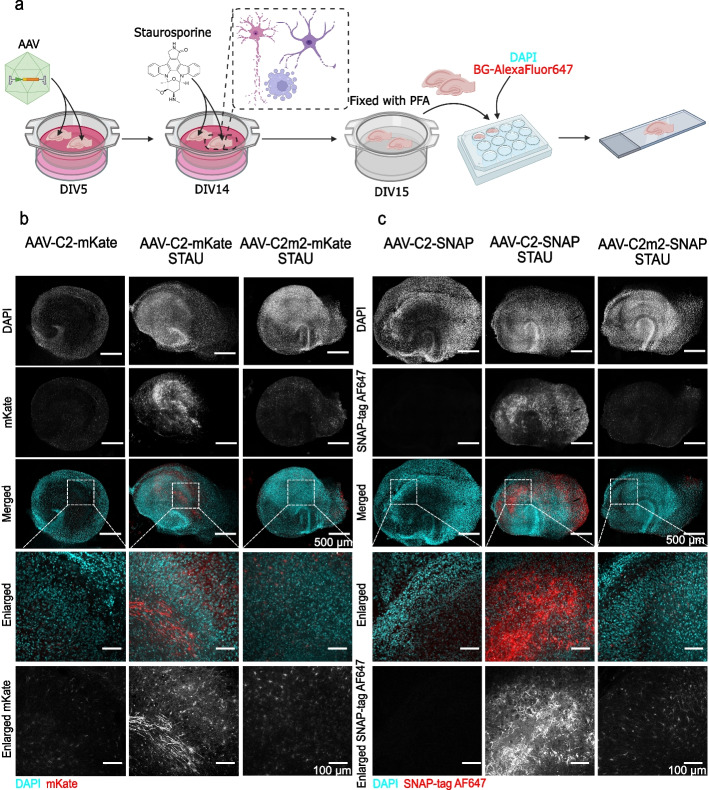
AAV-encoded C2 fusion probes label apoptotic cells in ex vivo organotypic hippocampal slices. **a** OHSC were transduced with AAVs carrying C2-SNAP, C2m2-SNAP, C2-mKate or C2m2-mKate at 5 days in vitro (5DIV) and maintained for 9 days for protein expression (*n* = 3 biological replicates). Apoptosis was induced by the treatment with 10 µM staurosporine for 16 h. Created by Biorender.com. **b**, **c** Confocal images of OHSC transduced with AAV-C2-mKate *vs*. AAV-C2m2-mKate or AAV-C2-SNAP vs. AAV-C2m2-SNAP. Cell nuclei were counterstained with DAPI. Scale bars 500 µm (whole slice) and 100 µm (enlarged area)

We then evaluated the capacity of AAV-encoded C2 fusion probes to label exposed PS in organotypic slices. OHSC were transduced with engineered AAVs on the DIV5, and apoptosis was induced by staurosporine 9 days later (Fig. 5a). We found that AAV-delivered C2-SNAP and C2-mKate fusion proteins efficiently labelled apoptotic cells in a tissue culture (Fig. 5b, c). The C2 probes visualised apoptotic neuron processes throughout the slice (Fig. 5b, c). Importantly, there was no labelling in viable organotypic slices without staurosporine treatment, indicating that AAV-delivered C2-SNAP and C2-mKate specifically bound to apoptotic cells (Fig. 5b, c). Furthermore, when AAVs delivered mutated C2m2 probes, neither cell bodies nor neuronal processes were labelled, and only a weak background signal was observed (Fig. 5b, c). Therefore, we propose that C2m2 probes could be used in parallel with C2 probes to serve as a negative control to evaluate any unspecific binding and to define thresholding for quantitative analysis. Our results reveal that C2 fusion probes can be efficiently expressed after AAV delivery and can be used to label exposed PS in a more complex experimental platform, such as tissue culture.

### AAV-C2-SNAP efficiently transduce the central nervous system in vivo

**Fig. 6:**
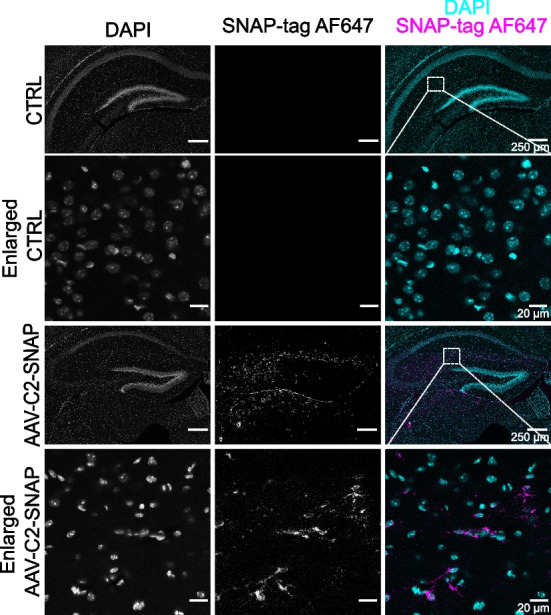
Application of adeno-associated viruses (AAV) for in vivo C2 probe delivery. At postnatal day 3 (P3) mice were injected with 1 × 10^11^ vg of AAV-C2-SNAP virus. Representative microscopy images of hippocampus after 2 weeks of AAV-C2-SNAP expression (P17) (*n* = 5 animals) and enlarged hippocampal areas. Cell nuclei were stained with DAPI, fused SNAP-tag was labelled with AlexaFluor647 (AF647). Control images represent BG-AF647 labelling of non-transduced brain tissue. Scale bar 250 µm (whole hippocampus) and 20 µm (enlarged area)

In order to investigate whether the genetically encoded C2-SNAP protein could be used for in vivo assays, we intravenously injected perinatal (P3) mice with neurotropic recombinant AAVs. After 2 weeks of in vivo expression, at P17, we observed C2-SNAP expression in the hippocampus, as expected during brain development (Fig. 6). These results confirmed that intravenous AAV-C2-SNAP injection was sufficient for brain transduction.

No specific labelling was observed in brain sections of control mice treated with only SNAP-tag substrate, without C2-SNAP expression (Fig. 6). These results demonstrated that genetically encoded C2-SNAP probe could be used for functional studies on PS exposure in vivo without invasive reagent delivery to the brain tissue.

## Discussion

In this study, we have developed a two-component PS labelling system based on the C2 domain of the MFG-E8 opsonin. The C2 probe can be used either as a purified protein or as a genetically encoded tool delivered by AAV. It is intended to be used in parallel with a C2m2 negative control, which was developed by mutating two amino acids in C2 domain, disabling the probe binding to PS. SPR experiments revealed high C2 domain specificity to PS compared to neutral membranes (Fig. 2b, c) in agreement with previous studies where PC membranes were used as a control [61]. Importantly, we demonstrated that the C2 domain and fused C2 probes did not bind to another abundant phospholipid, PE, indicating that the C2 domain specifically recognizes PS rather than negatively charged phospholipids (Fig. 2). It is an advantage in comparison to other reagents for PS labelling that lack PS over PE selectivity [10, 19]. SPR experiments also confirmed that the mutated C2m2 domain does not bind to PS (Fig. 2f, g, j, k) and could therefore be used to assess the background signal both qualitatively and quantitatively. Fused mKate and SNAP-tags did not interfere with C2 domain’s interaction with PS and did not introduce any nonspecific interactions with phospholipid membranes, ensuring the applicability of fused C2 probes (Fig. 2d, e, h, i).

As artificial membranes cannot fully replicate the complete composition of cellular plasma membranes, we employed the probes on in vitro cell cultures and demonstrated that C2-SNAP and C2-mKate fusion proteins effectively labelled apoptosis in different cell lines (Fig. 3a–d, Additional file 2: Fig. S5a–d). C2 probes provided more precise and efficient visualisation of PS-exposing cells compared to another currently available tool, PSVue (Fig. 3g, Additional file 2: Fig. S5g).

Cell monolayer cultures are insufficient when assessing complex physiological processes. In contrast, 3D cell cultures, tissue cultures, or in vivo experiments can capture intracellular interactions more accurately. However, it is difficult to apply recombinant proteins or small molecule probes in 3D tissue due to the limited access to cell membranes. The local injections of genetically encoded plasmids [27, 62], chemical reagents [20] or proteins [63] have limited permeability and can also cause tissue damage, potentially leading to apoptosis or necrosis, which in turn are marked by PS exposure [64]. This problem can be solved by using gene delivery tools. AAV delivery is a potential technique to label exposed PS ex vivo and in vivo. Here we demonstrated that C2 probes can be designed to be selectively targeted to the chosen cell type and can be efficiently secreted for PS externalisation assays (Figs. 4, 5 Additional file 1: Fig. S6–S9). We observed that C2-SNAP proteins were expressed in the mouse brain transduced by AAVs 2 weeks after gene delivery (Fig. 6), consistent with other studies that used the same PHP.eB capsid in mice [47, 65]. Importantly, genetically encoded probes are readily modifiable and can be applied to any cell type of interest by changing the expression promoter [66] and the tropism of the used AAV capsid [67]. Therefore, AAVs encoding C2 probes could be applied in other areas than brain development research, such as blood clotting studies, by changing the promoter to target blood or epithelium cells to secrete C2 probe for the labelling of exposed PS on platelets. This system should then be supplemented with a specific AAV serotype for efficient transduction of the chosen cell type. Genetically encoded C2 fusion probes can be adapted for different visualisation applications as well. If mKate was not suitable for a particular experiment, a different fluorescent protein (e.g. green and yellow) or other red variants could be chosen. In addition, to obtain different visualisation spectra C2-SNAP probes could be easily modified by selecting a SNAP-tag substrate with required organic dye. This ensures the versatility of the C2 probe system presented in this paper.

Finally, we demonstrated that AAV-delivered C2 probes can be used for uninvasive labelling of brain tissue. It is known that the mouse brain is developing approximately until the 30th day after birth (P30) [68, 69]. During brain development, synaptic pruning is a critical process in which PS-exposing synapses are eliminated [70]. Therefore, it is important to have PS labelling tools that can be delivered to the postnatal animals without disturbing nervous tissue. In our in vivo study, we localized C2-SNAP protein labelling in the developing hippocampus (Fig. 6). This demonstrated that C2-SNAP can be applied to label not only apoptotic cells, but also physiologically exposed PS.

In conclusion, the selectivity and specificity of the C2 domain allowed us to create a genetically encoded and modifiable PS labelling probe for in vitro, ex vivo and in vivo applications. This tool can be applied in various assays as a two-component system consisting of C2 and C2m2 fusion proteins for PS visualisation and precise quantification. By directly defining threshold levels, it enables to assess PS exposure both in cell death and in physiological processes.

## Supplementary Information

**Additional file 1: Table S1.** C2-mKate structural models obtained by Robetta and Raptor-X servers and assessed with VoroMQA, ProSA, QMEANDisCo and ProQ2 tools. **Table S2.** C2-SNAP structural models obtained by Robetta and Raptor-X servers and assessed with VoroMQA, ProSA, QMEANDisCo and ProQ2 tools. **Table S3.** *E. coli* strains and conditions used to express recombinant C2-mKate and C2-SNAP proteins. **Figure S1.** CellProfiler pipeline used in this study. **Figure S2.** Colocalization analysis of astrocytes and C2-probes in OHSC. **Figure S3.** Structural models of recombinant C2 fusion proteins obtained on *AlphaFold 2*. **Figure S4.** The plasmids of C2 probes for protein expression or viral delivery. **Figure S5.** Evaluation of the binding of C2 probes to apoptotic HEK293T cells. **Figure S6.** The expression of C2-mKate fusion proteins in ex vivo organotypic hippocampal slices after AAV delivery. **Figure S7.** The expression of C2 fusion proteins in astrocytes after AAV delivery. **Figure S8.** The expression of C2 fusion proteins in microglia after AAV delivery. **Figure S9.** The expression of C2 fusion proteins in pyramidal neurons after AAV delivery.

**Additional file 2.** Plasmid sequences.

**Additional file 3.** CellProfiler pipeline.

## Data Availability

The data supporting the findings of this study are available within the article and its Additional files. If specific data is believed to be missing, that data is available from the corresponding author upon request. The plasmids for expression of C2-SNAP, C2-mKate proteins and its mutant version will be made available through Addgene.

## Abbreviations

AAV: Adeno-associated virus
BG: Benzylguanine
CHOL: Cholesterol
DOPC: 1,2-Dioleoyl-sn-glycero-3-phosphocholine
DOPE: 1,2-Dioleoyl-sn-glycero-3-phosphoethanolamine
DOPS: 1,2-Dioleoyl-sn-glycero-3-phospho-l-serine
hBLM: Hybrid bilayer lipid membranes
MLV: Multilamellar vesicles
OHSC: Organotypic hippocampal slice culture
PS: Phosphatidylserine
SAM: Self-assembled monolayer
STAU: Staurosporine

## References

[1] Vance JE (2003). Molecular and cell biology of phosphatidylserine and phosphatidylethanolamine metabolism. Prog Nucleic Acid Re.

[2] van Meer G, Voelker DR, Feigenson GW (2008). Membrane lipids: where they are and how they behave. Nat Rev Mol Cell Biol.

[3] Fadok VA, Bratton DL, Rose DM, Pearson A, Ezekewitz RA, Henson PM (2000). A receptor for phosphatidylserine-specific clearance of apoptotic cells. Nature.

[4] Bevers EM, Williamson PL (2016). Getting to the outer leaflet: physiology of phosphatidylserine exposure at the plasma membrane. Physiol Rev.

[5] Crowley LC, Marfell BJ, Scott AP, Waterhouse NJ (2016). Quantitation of apoptosis and necrosis by annexin v binding, propidium iodide uptake, and flow cytometry. Cold Spring Harb Protoc.

[6] Prinzen L, Miserus RJJHM, Dirksen A, Hackeng TM, Deckers N, Bitsch NJ (2007). Optical and magnetic resonance imaging of cell death and platelet activation using annexin A5-functionalized quantum dots. Nano Lett.

[7] Dachary-Prigent J, Freyssinet J, Pasquet J, Carron J, Nurden A (1993). Annexin V as a probe of aminophospholipid exposure and platelet membrane vesiculation: a flow cytometry study showing a role for free sulfhydryl groups. Blood.

[8] Logue SE, Elgendy M, Martin SJ (2009). Expression, purification and use of recombinant annexin V for the detection of apoptotic cells. Nat Protoc.

[9] Hu T, Shi J, Jiao X, Zhou J, Yin X (2008). Measurement of annexin V uptake and lactadherin labeling for the quantification of apoptosis in adherent Tca8113 and ACC-2 cells. Braz J Med Biol Res.

[10] Meers P, Mealy T (1994). Phospholipid determinants for annexin V binding sites and the role of tryptophan 187. Biochemistry.

[11] Kim YE, Chen J, Chan JR, Langen R (2010). Engineering a polarity-sensitive biosensor for time-lapse imaging of apoptotic processes and degeneration. Nat Methods.

[12] Kim YE, Chen J, Langen R, Chan JR (2010). Monitoring apoptosis and neuronal degeneration by real-time detection of phosphatidylserine externalization using a polarity-sensitive indicator of viability and apoptosis. Nat Protoc.

[13] Maneta-Peyret L, Freyburger G, Bessoule JJ, Cassagne C (1989). Specific immunocytochemical visualization of phosphatidylserine. J Immunol Methods.

[14] Yeung T, Heit B, Dubuisson JF, Fairn GD, Chiu B, Inman R (2009). Contribution of phosphatidylserine to membrane surface charge and protein targeting during phagosome maturation. J Cell Biol.

[15] Schick PK, Kurica KB, Chacko GK (1976). Location of phosphatidylethanolamine and phosphatidylserine in the human platelet plasma membrane. J Clin Investig.

[16] Kwong JMK, Hoang C, Dukes RT, Yee RW, Gray BD, Pak KY (2014). Bis(Zinc-dipicolylamine), Zn-DPA, a new marker for apoptosis. Investig Ophthalmol Vis Sci.

[17] Cho YS, Kim KM, Lee D, Kim WJ, Ahn KH (2013). Turn-on fluorescence detection of apoptotic cells using a Zinc(II)-dipicolylamine-functionalized poly(diacetylene) liposome. Chem Asian J.

[18] Boon JM, Smith BD (2002). Chemical control of phospholipid distribution across bilayer membranes. Med Res Rev.

[19] Scott HL, Heberle FA, Katsaras J, Barrera FN (2019). Phosphatidylserine asymmetry promotes the membrane insertion of a transmembrane helix. Biophys J.

[20] Scott-Hewitt N, Perrucci F, Morini R, Erreni M, Mahoney M, Witkowska A (2020). Local externalization of phosphatidylserine mediates developmental synaptic pruning by microglia. EMBO J.

[21] Martin OC, Pagano RE (1987). Transbilayer movement of fluorescent analogs of phosphatidylserine and phosphatidylethanolamine at the plasma membrane of cultured cells. Evidence for a protein-mediated and ATP-dependent process(es). J Biol Chem.

[22] Nichols JW (2002). Internalization and trafficking of fluorescent-labeled phospholipids in yeast. Semin Cell Dev Biol.

[23] Oshima K, Yasueda T, Nishio S, Matsuda T (2014). Chapter 1, MFG-E8: origin, structure, expression, functions and regulation. MFG-E8 and inflammation.

[24] Wang P (2014). MFG-E8 and inflammation.

[25] Reddy Nanga RP, Vivekanandan S, Yoon HS (2007). Expression, purification and characterization of C2 domain of milk fat globule-EGF-factor 8-L. Protein Expr Purif.

[26] Ye H, Li B, Subramanian V, Choi BH, Liang Y, Harikishore A (2013). NMR solution structure of C2 domain of MFG-E8 and insights into its molecular recognition with phosphatidylserine. Biochim Biophys Acta Biomembr.

[27] Furuta Y, Pena-Ramos O, Li Z, Chiao L, Zhou Z (2021). Calcium ions trigger the exposure of phosphatidylserine on the surface of necrotic cells. PLoS Genet.

[28] Kay JG, Koivusalo M, Ma X, Wohland T, Grinstein S (2012). Phosphatidylserine dynamics in cellular membranes. Mol Biol Cell.

[29] Yeung T, Gilbert GE, Shi J, Silvius J, Kapus A, Grinstein S (2008). Membrane phosphatidylserine regulates surface charge and protein localization. Science.

[30] Shcherbo D, Merzlyak EM, Chepurnykh TV, Fradkov AF, Ermakova GV, Solovieva EA (2007). Bright far-red fluorescent protein for whole-body imaging. Nat Methods.

[31] Keppler A, Gendreizig S, Gronemeyer T, Pick H, Vogel H, Johnsson K (2003). A general method for the covalent labeling of fusion proteins with small molecules in vivo. Nat Biotechnol.

[32] Baek M, DiMaio F, Anishchenko I, Dauparas J, Ovchinnikov S, Lee GR (2021). Accurate prediction of protein structures and interactions using a three-track neural network. Science.

[33] Källberg M, Wang H, Wang S, Peng J, Wang Z, Lu H (2012). Template-based protein structure modeling using the RaptorX web server. Nat Protoc.

[34] Mirdita M, Schütze K, Moriwaki Y, Heo L, Ovchinnikov S, Steinegger M (2022). ColabFold: making protein folding accessible to all. Nat Methods.

[35] Jumper J, Evans R, Pritzel A, Green T, Figurnov M, Ronneberger O (2021). Highly accurate protein structure prediction with AlphaFold. Nature.

[36] Olechnovič K, Venclovas Č (2017). VoroMQA: assessment of protein structure quality using interatomic contact areas. Proteins Struct Funct.

[37] Wiederstein M, Sippl MJ (2007). ProSA-web: interactive web service for the recognition of errors in three-dimensional structures of proteins. Nucleic Acids Res.

[38] Studer G, Rempfer C, Waterhouse AM, Gumienny R, Haas J, Schwede T (2020). QMEANDisCo—distance constraints applied on model quality estimation. Bioinformatics.

[39] Ray A, Lindahl E, Wallner B (2012). Improved model quality assessment using ProQ2. BMC Bioinform.

[40] Pletnev S, Shcherbo D, Chudakov DM, Pletneva N, Merzlyak EM, Wlodawer A (2008). A crystallographic study of bright far-red fluorescent protein mKate reveals pH-induced cis-trans isomerization of the chromophore. J Biol Chem.

[41] Chen X, Zaro JL, Shen WC (2013). Fusion protein linkers: property, design and functionality. Adv Drug Deliv Rev.

[42] Dhandapani R, Arokiaraj CM, Taberner FJ, Pacifico P, Raja S, Nocchi L (2018). Control of mechanical pain hypersensitivity in mice through ligand-targeted photoablation of TrkB-positive sensory neurons. Nat Commun.

[43] Stubbs JD, Lekutis C, Singer KL, Bui A, Yuzuki D, Srinivasan U (1990). cDNA cloning of a mouse mammary epithelial cell surface protein reveals the existence of epidermal growth factor-like domains linked to factor VIII-like sequences. Proc Natl Acad Sci USA.

[44] Ragaliauskas T, Mickevicius M, Rakovska B, Penkauskas T, Vanderah DJ, Heinrich F (2017). Fast formation of low-defect-density tethered bilayers by fusion of multilamellar vesicles. Biochim Biophys Acta Biomembr.

[45] Stirling DR, Carpenter AE, Cimini BA (2021). Cell Profiler Analyst 3.0: accessible data exploration and machine learning for image analysis. Bioinformatics.

[46] Challis RC, Kumar SR, Chan KY, Challis C, Jang MJ, Rajendran PS (2018). Widespread and targeted gene expression by systemic AAV vectors: production, purification, and administration. Nat Protoc.

[47] Chan KY, Jang MJ, Yoo BB, Greenbaum A, Ravi N, Wu WL (2017). Engineered AAVs for efficient noninvasive gene delivery to the central and peripheral nervous systems. Nat Neurosci.

[48] de Simoni A, MY Yu L (2006). Preparation of organotypic hippocampal slice cultures: interface method. Nat Protoc.

[49] Gogolla N, Galimberti I, DePaola V, Caroni P (2006). Preparation of organotypic hippocampal slice cultures for long-term live imaging. Nat Protoc.

[50] Schindelin J, Arganda-Carreras I, Frise E, Kaynig V, Longair M, Pietzsch T (2012). Fiji: an open-source platform for biological-image analysis. Nat Methods.

[51] Merlo R, Caprioglio D, Cillo M, Valenti A, Mattossovich R, Morrone C (2021). The SNAP- tag technology revised: an effective chemo-enzymatic approach by using a universal azide-based substrate. J Enzyme Inhib Med Chem.

[52] Deo C, Lavis LD (2018). Synthetic and genetically encoded fluorescent neural activity indicators. Curr Opin Neurobiol.

[53] Argos P (1990). An investigation of oligopeptides linking domains in protein tertiary structures and possible candidates for general gene fusion. J Mol Biol.

[54] George RA, Heringa J (2002). An analysis of protein domain linkers: their classification and role in protein folding. Protein Eng Des Sel.

[55] Berman HM (2000). The protein data bank. Nucleic Acids Res.

[56] Ferrer M, Chernikova TN, Yakimov MM, Golyshin PN, Timmis KN (2003). Chaperonins govern growth of *Escherichia coli* at low temperatures. Nat Biotechnol.

[57] Bhatta M, Shenoy GN, Loyall JL, Gray BD, Bapardekar M, Conway A (2021). Novel phosphatidylserine-binding molecule enhances antitumor T-cell responses by targeting immunosuppressive exosomes in human tumor microenvironments. J Immunother Cancer.

[58] Brenner M, Kisseberth W, Su Y, Besnard F, Messing A (1994). GFAP promoter directs astrocyte-specific expression in transgenic mice. J Neurosci.

[59] Linsley JW, Tripathi A, Epstein I, Schmunk G, Mount E, Campioni M (2019). Automated four-dimensional long term imaging enables single cell tracking within organotypic brain slices to study neurodevelopment and degeneration. Commun Biol.

[60] Nebenführ A, Ritzenthaler C, Robinson DG (2002). Brefeldin A: deciphering an enigmatic inhibitor of secretion. Plant Physiol.

[61] Del Vecchio K, Stahelin RV (2018). Investigation of the phosphatidylserine binding properties of the lipid biosensor, Lactadherin C2 (LactC2), in different membrane environments. J Bioenergy Biomembr.

[62] Ham TJ, Mapes J, Kokel D, Peterson RT (2010). Live imaging of apoptotic cells in zebrafish. FASEB J.

[63] Blankenberg FG, Katsikis PD, Tait JF, Davis RE, Naumovski L, Ohtsuki K (1998). In vivo detection and imaging of phosphatidylserine expression during programmed cell death. Proc Natl Acad Sci USA.

[64] Sapar ML, Ji H, Wang B, Poe AR, Dubey K, Ren X (2018). Phosphatidylserine externalization results from and causes neurite degeneration in *Drosophila*. Cell Rep.

[65] Goertsen D, Flytzanis NC, Goeden N, Chuapoco MR, Cummins A, Chen Y (2022). AAV capsid variants with brain-wide transgene expression and decreased liver targeting after intravenous delivery in mouse and marmoset. Nat Neurosci.

[66] Becker J, Fakhiri J, Grimm D (2022). Fantastic AAV gene therapy vectors and how to find them—random diversification, rational design and machine learning. Pathogens.

[67] Brown D, Altermatt M, Dobreva T, Chen S, Wang A, Thomson M (2021). Deep parallel characterization of AAV tropism and AAV-mediated transcriptional changes via single-cell RNA sequencing. Front Immunol.

[68] Mody M, Cao Y, Cui Z, Tay KY, Shyong A, Shimizu E (2001). Genome-wide gene expression profiles of the developing mouse hippocampus. Proc Natl Acad Sci USA.

[69] Navlakha S, Barth AL, Bar-Joseph Z (2015). Decreasing-rate pruning optimizes the construction of efficient and robust distributed networks. PLoS Comput Biol.

[70] Kurematsu C, Sawada M, Ohmuraya M, Tanaka M, Kuboyama K, Ogino T (2022). Synaptic pruning of murine adult-born neurons by microglia depends on phosphatidylserine. J Exp Med.

