# Supplementary material for: Genetically encoded phosphatidylserine biosensor for in vitro, ex vivo and in vivo labelling
Source: Cell Mol Biol Lett. 2023 Jul 27;28:59. doi: 10.1186/s11658-023-00472-7 (PMC10373266; doi:10.1186/s11658-023-00472-7)
Supplement: Supplementary file 1 — Additional file 1: Table S1. C2-mKate structural models obtained by Robetta and Raptor-X servers and assessed with VoroMQA, ProSA, QMEANDisCo and ProQ2 tools. Table S2. C2-SNAP structural models obtained by Robetta and Raptor-X servers and assessed with VoroMQA, ProSA, QMEANDisCo and ProQ2 tools. Table S3. E. coli strains and conditions used to express recombinant C2-mKate and C2-SNAP proteins. Figure S1. CellProfiler pipeline used in this study. Figure S2. Colocalization analysis of astrocytes and C2-probes in OHSC. Figure S3. Structural models of recombinant C2 fusion proteins obtained on AlphaFold 2. Figure S4. The plasmids of C2 probes for protein expression or viral delivery. Figure S5. Evaluation of the binding of C2 probes to apoptotic HEK293T cells. Figure S6. The expression of C2-mKate fusion proteins in ex vivo organotypic hippocampal slices after AAV delivery. Figure S7. The expression of C2 fusion proteins in astrocytes after AAV delivery. Figure S8. The expression of C2 fusion proteins in microglia after AAV delivery. Figure S9. The expression of C2 fusion proteins in pyramidal neurons after AAV delivery. [file 11658_2023_472_MOESM1_ESM.pdf]

Additional File 1 for

Genetically encoded phosphatidylserine biosensor for in vitro, ex vivo and in vivo labelling

Eimina Dirvelyte[1], Daina Bujanauskiene[1,2], Evelina Jankaityte[1,3], Neringa Daugelaviciene[1], Ugne Kisieliute[2], Igor Nagula[1], Rima Budvytyte[1,3], Urte Neniskyte[1,2]*

[1]VU LSC-EMBL Partnership for Genome Editing Technologies, Life Sciences Center, Vilnius University, Vilnius, Lithuania

[2]Institute of Bioscience, Life Sciences Center, Vilnius University, Vilnius, Lithuania

[3]Institute of Biochemistry, Life Sciences Center, Vilnius University, Vilnius, Lithuania

*Corresponding author: urte.neniskyte@gmc.vu.lt

This file includes:

Tables S1-3
Figures S1-9

Additional Tables

Table S1. C2-mKate structural models obtained by Robetta and Raptor-X servers and assessed with VoroMQA, ProSA, QMEANDisCo and ProQ2 tools. Colors in the models depict local amino acid probability. The highest value for each quality assessment tool is in bold. Robetta server predicted structures as protein dimers. The best model is in bold.

**Protein model** 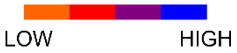	**ProQ2 Global quality**	**VoroMQA**	**QMEAN DisCo**	**ProSA**
Robetta 1 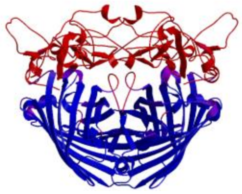	375,634	0,43	0,55 ± 0,05	-6,13
Robetta 2 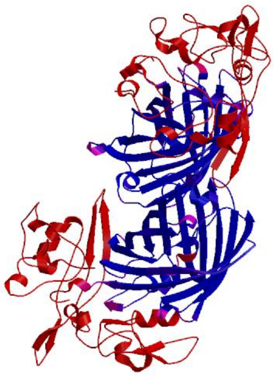	362,703	0,4	0,53 ± 0,05	-6,23
Robetta 3 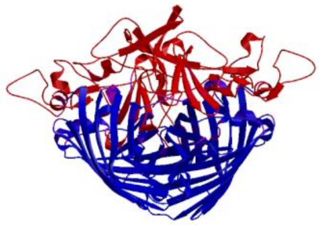	**389,388**	0,42	0,56 ± 0,05	-6,41
Robetta 4 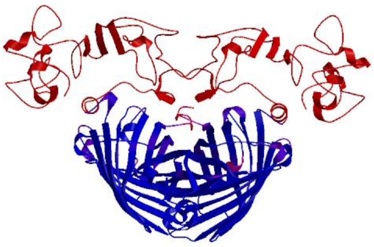	376,111	0,422	0,56 ± 0,05	-6,33

Robetta 5 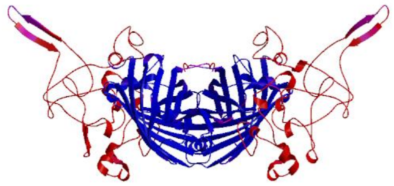	376,111	0,418	0,57 ± 0,05	-6,55
RaptorX 1 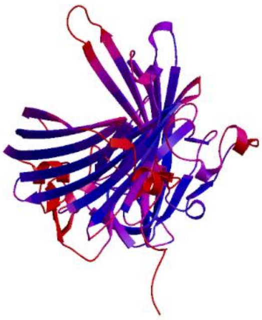	247,604	0,361	0,59 ± 0,05	-6,36
RaptorX 2 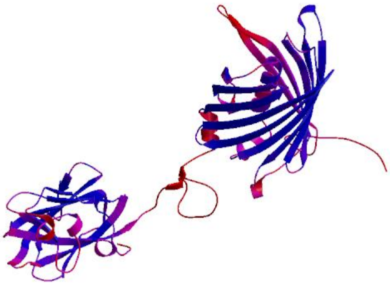	286,092	0,437	0,64 ±0,052	-6,79
RaptorX 3 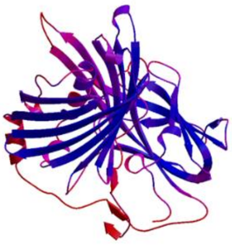	285,423	0,438	0,63 ± 0,05	**-7,32**
RaptorX 4 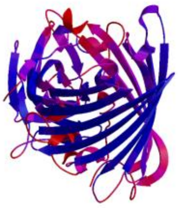	290,405	**0,452**	0,62 ± 0,05	-6,31
**RaptorX 5** 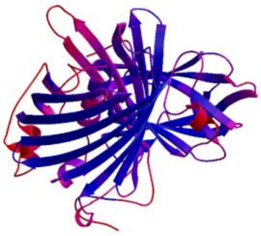	285,739	0,443	**0,65 ± 0,05**	-6,99

Table S2. C2-SNAP structural models obtained by Robetta and Raptor-X servers and assessed with VoroMQA, ProSA, QMEANDisCo and ProQ2 tools. Colors in the models depict local amino acid probability. The highest value for each quality assessment tool is in bold. The best model is in bold.

**Protein model** 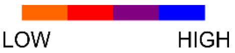 LOW HIGH	**ProQ2 Global quality**	**VoroMQA**	**QMEAN DisCo**	**ProSA**
Robetta 1 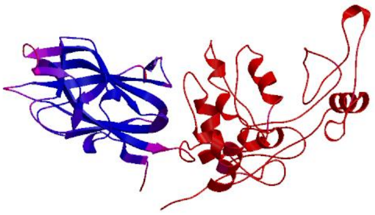	163,236	0,358	0,43 ± 0,05	-6,23
Robetta 2 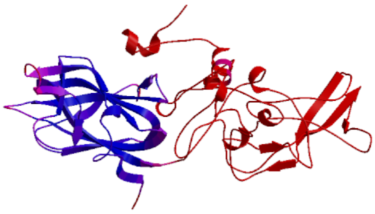	**376,111**	0,357	0,48 ± 0,05	-6,51
Robetta 3 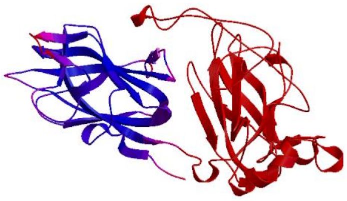	164,970	0,349	0,45 ± 0,05	-6,52
Robetta4 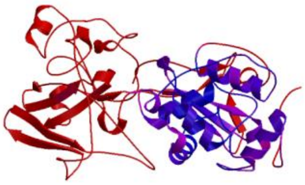	**376,111**	0,331	0,45 ± 0,05	-6,30
Robetta5 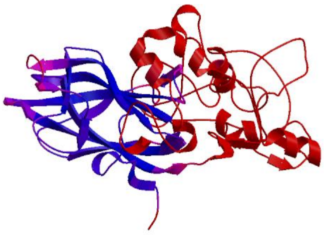	147,338	0,314	0,45 ± 0,05	-6,22

RaptorX 1 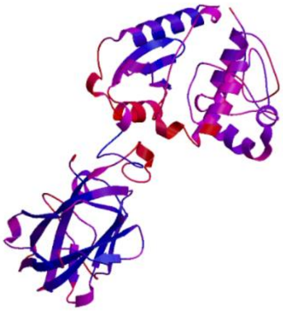	234,384	0,392	**0,63 ± 0,05**	-6,56
RaptorX 2 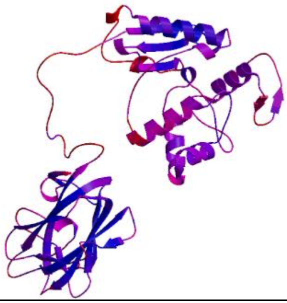	234,421	0,388	0,62 ± 0,05	-6,54
**RaptorX 3** 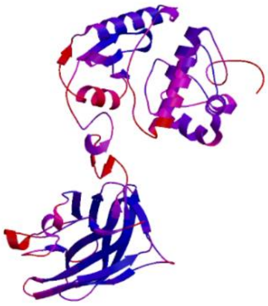	244,861	**0,4**	**0,63 ± 0,05**	**-6,75**
RaptorX 4 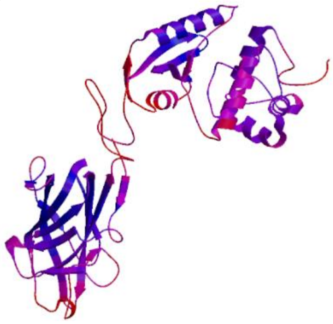	236,773	0,388	0,61 ± 0,05	-6,52
RaptorX 5 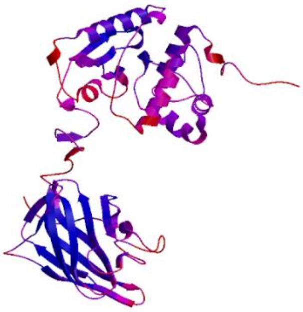	236,773	0,385	0,62 ± 0,05	-6,51

Table S3. *E. coli* strains and conditions used to express recombinant C2-mKate and C2-SNAP proteins.

	IPTG (mM)		Induction temperature (°C)				NaCl (mM)		pH	
BL21(DE3)	0.5	1.0		16	20	25	300	400	7.3	8.0
Rosetta-gami 2(DE3)	0.5	1.0		16	20	25	300	400	7.3	8.0
HMS174(DE3)	0.5	1.0		16	20	25	300	400	7.3	8.0
C43(DE3)pLysS	0.5	1.0		16	20	25	300	400	7.3	8.0
NovaBlue(DE3)	0.5	1.0		16	20	25	300	400	7.3	8.0
**ArcticExpress(DE3)**[*]	0.5	**1.0**[*]	13	**16**[*]			**300**[*]	400	7.3	**8.0**[*]

[*] - the conditions that resulted in the highest content of soluble recombinant proteins

Additional Figures

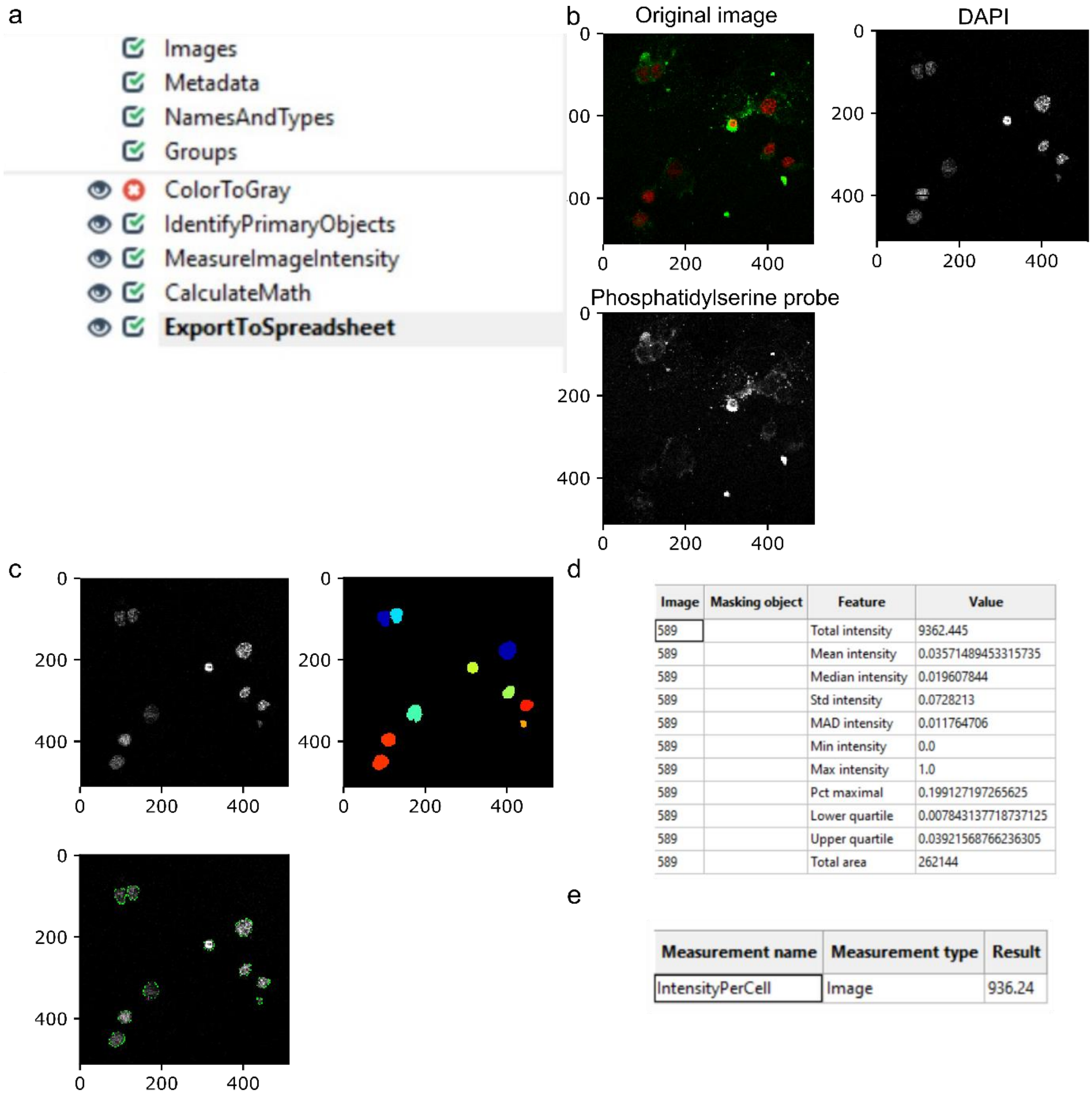

Figure S1 CellProfiler pipeline used in this study. a Image analysis on CellProfiler consisted of 5 steps. **b** *ColorToGray* was used to split fluorescence channels of DAPI and C2 probe. **c** The *IdentifyPrimaryObjects* function was used to detect nuclei. **d** The *MeasureImageIntensity* function was used to quantify the total C2 probe signal in the image. **e** *CalculateMath* was used to divide C2 probe signal by cell nuclei count in an image to obtain average signal per single cell.

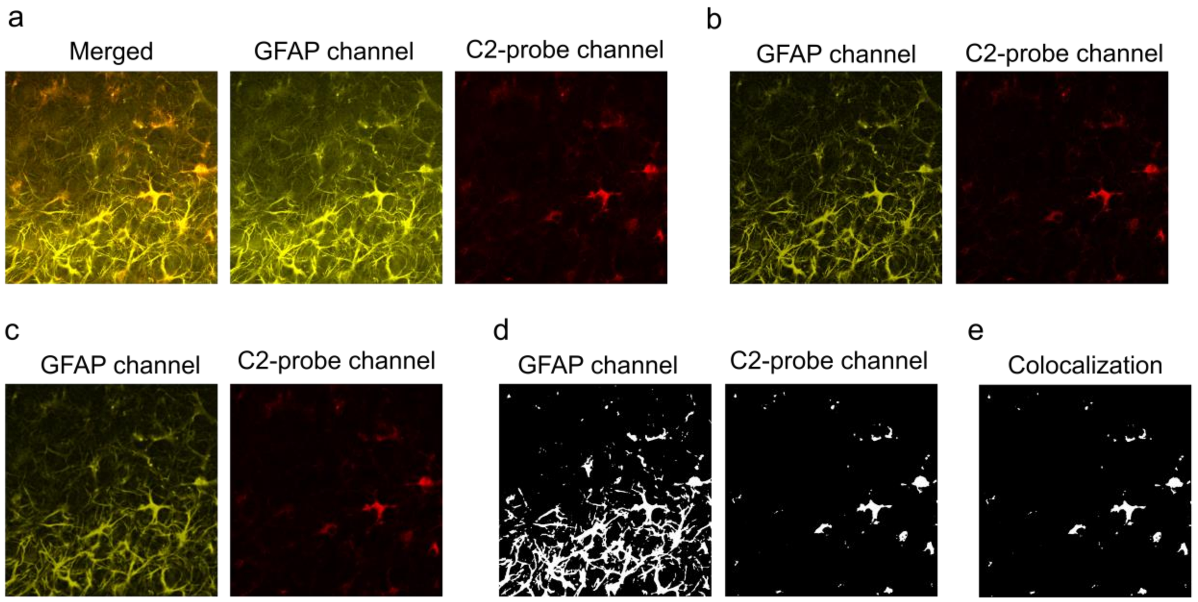

Figure S2 Colocalization analysis of astrocytes and C2-probes in OHSC. a GFAP and C2-probe channels were used for quantitative colocalization analysis on ImageJ software. **b** First, background was subtracted from both channels using the *Subtract background* (rolling ball radius 50 pixels) function. **c** Images were smoothed with the *Gaussian Blur* filter (sigma = 2). **d** Images were thresholded by *Auto-Threshold* using Otsu algorithm to obtain masks for further measurements. **e** The image of C2-probe signal within the astrocytes was obtained by the *Process-Image Calculator* function with *Operation = AND*. The area of GFAP (**d**) and SNAP within GFAP (**e**) was determined using the *Measure* function.

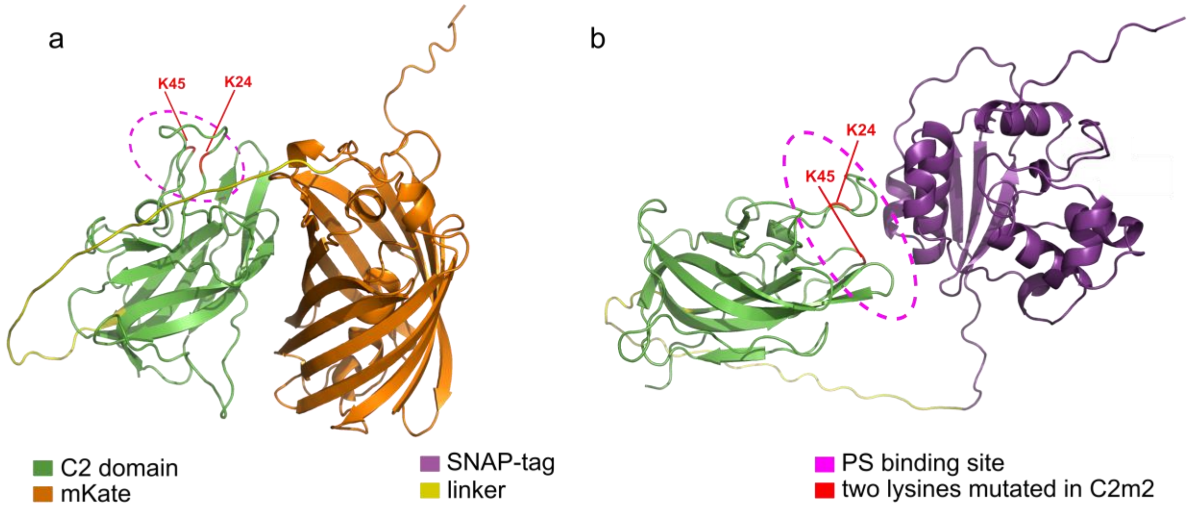

Figure S3 Structural models of recombinant C2 fusion proteins obtained on *AlphaFold 2*. a a C2-mKate structure predicted by *AlphaFold 2*. **b** C2-SNAP structure predicted by *AlphaFold 2*. Purple dashed circle marks putative site of C2 and PS interaction, including critical amino acids K24 and K45 in red.

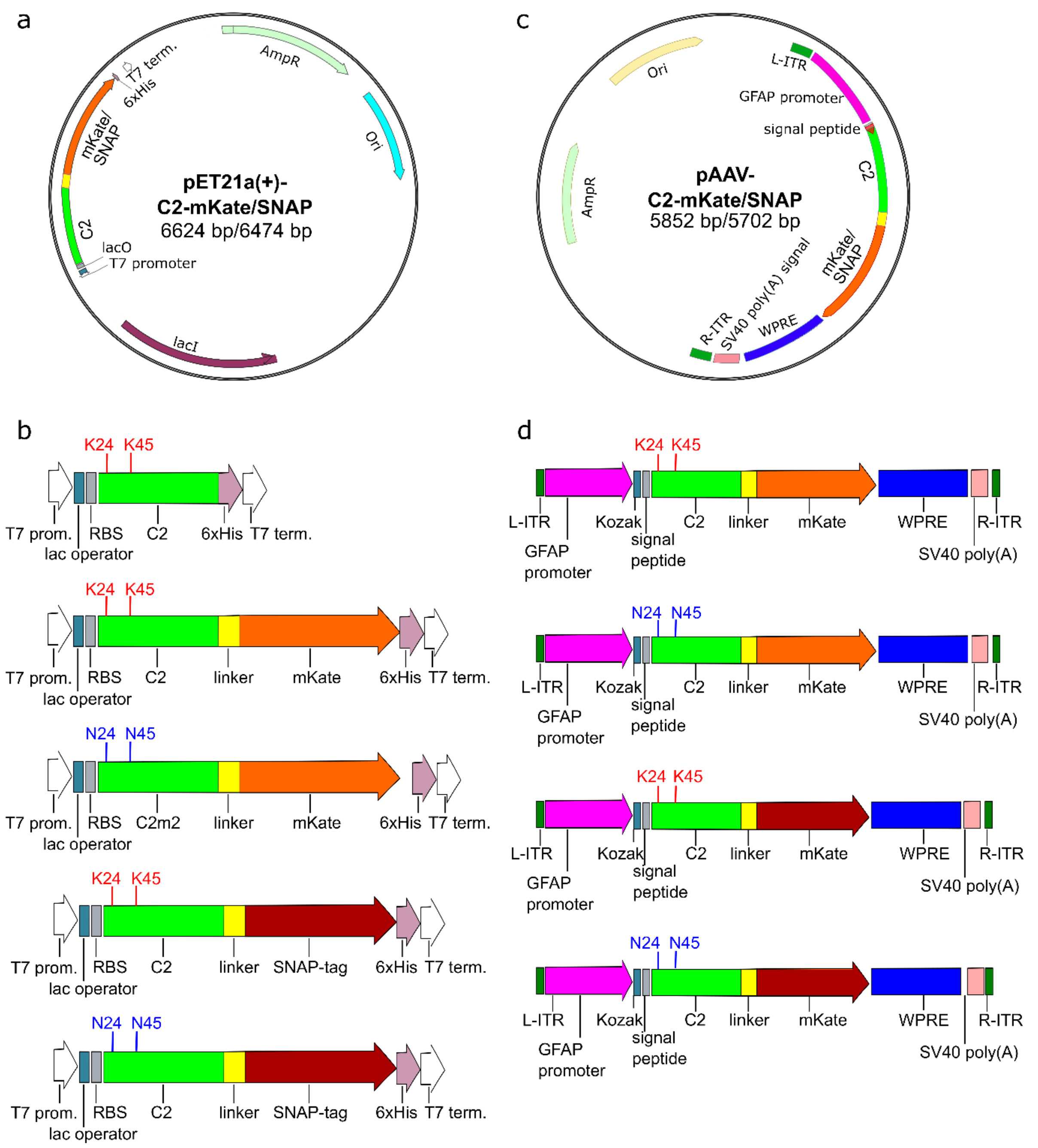

Figure S4 The plasmids of C2 probes for protein expression or viral delivery. a pET21a(+) plasmid for recombinant C2-mKate or C2-SNAP expression in *E.coli*. **b** The constructs encoding C2 fusion proteins for bacterial expression. From top to bottom: C2, C2-mKate, C2m2-mKate, C2-SNAP, C2m2-SNAP. In mutated C2 domain (C2m2), lysines K24, K45 were substituted for asparagines N24, N45. The tag of six histidines at the end of each recombinant protein was introduced for protein purification. **C** pAAV plasmid for C2-mKate or C2-SNAP viral delivery to cells and tissues. **d** The constructs in pAAV plasmid for viral C2 probe delivery and expression in astrocytes. From top to bottom: C2-mKate, C2m2-mKate, C2-SNAP, C2m2-SNAP. In mutated C2 domain (C2m2), lysines (K24, K45) were substituted for asparagines N24, N45. Transgene was located between two inverted terminal repeat sequences (ITR) for packaging in AAV. Glial fibrillary acidic protein (GFAP) promoter is required for targeted expression in astrocytes and is followed by Kozak sequence for translation initiation. After C2 domain, Woodchuck Hepatitis Virus Posttranscriptional Regulatory Element (WPRE) to enhance expression and Simian virus 40 (SV40) polyA signal to initiate transcript polyadenylation were located.

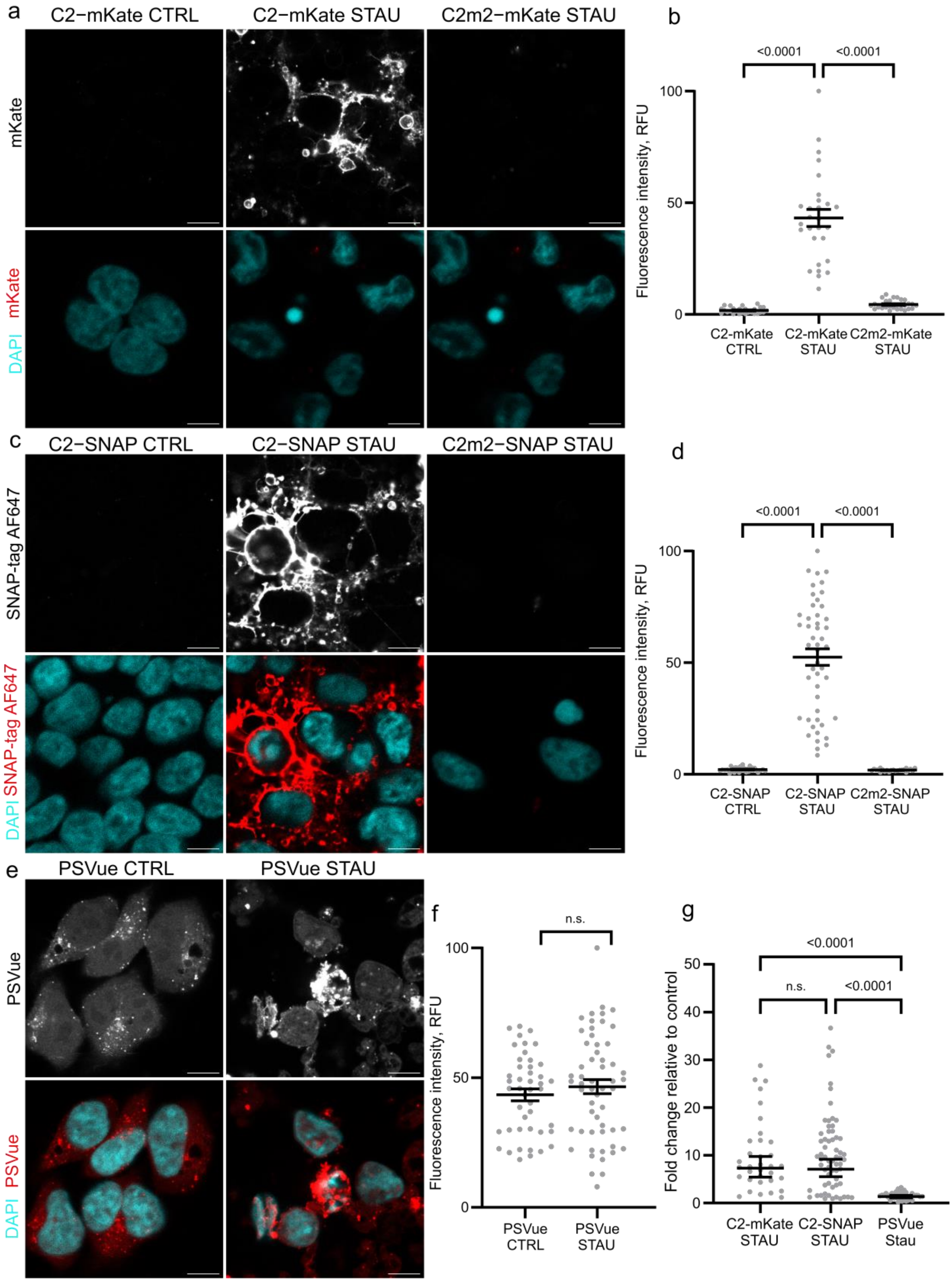

Figure S5 Evaluation of the binding of C2 probes to apoptotic HEK293T cells. a Confocal images of C2-mKate or C2m2-mKate binding to apoptotic or non-apoptotic HEK293T cells. CTRL – non-apoptotic cells, STAU – apoptotic HEK293T cells treated with 3 µM staurosporine. **b** The quantification of mKate fluorescence intensity on HEK293T cells under different conditions: C2-mKate on staurosporine treated or non-treated cells and C2m2-mKate on staurosporine treated cells. **c** Confocal images of C2-SNAP or C2m2-SNAP binding to apoptotic or non-apoptotic HEK293T cells. CTRL – non-apoptotic cells, STAU – apoptotic HEK293T cells treated with 3 µM staurosporine. **d** The quantification of SNAP-tag AlexaFluor647 (AF647) fluorescence intensity on HEK293T cells under different conditions: C2-SNAP on staurosporine treated or non-treated cells and C2m2-SNAP on staurosporine treated cells. **e** Confocal images of PSVue binding to non-apoptotic or apoptotic HEK293T cells.

CTRL – non-apoptotic cells, STAU – apoptotic HEK293T cells treated with 3 µM staurosporine. **f** The quantification of PSVue fluorescence intensity on HEK293T cells under two different conditions: PSVue on staurosporine treated or non-treated cells. **g** The comparison of C2 probes and PSVue. Data presented as means ± standard error of the mean (**b**, **d**, **f**) or geometrical means ± 95% confidence intervals (**g**) (n=20-30 images per biological replicate, 3 independent biological replicates). Means were compared by one-way ANOVA and *post-hoc* Tukey test (**b**, **d**, **f**) or by Kruskal-Wallis one-way analysis of variance and *post-hoc* Dunn's test (**g**). p values < 0.05 were considered as significant.

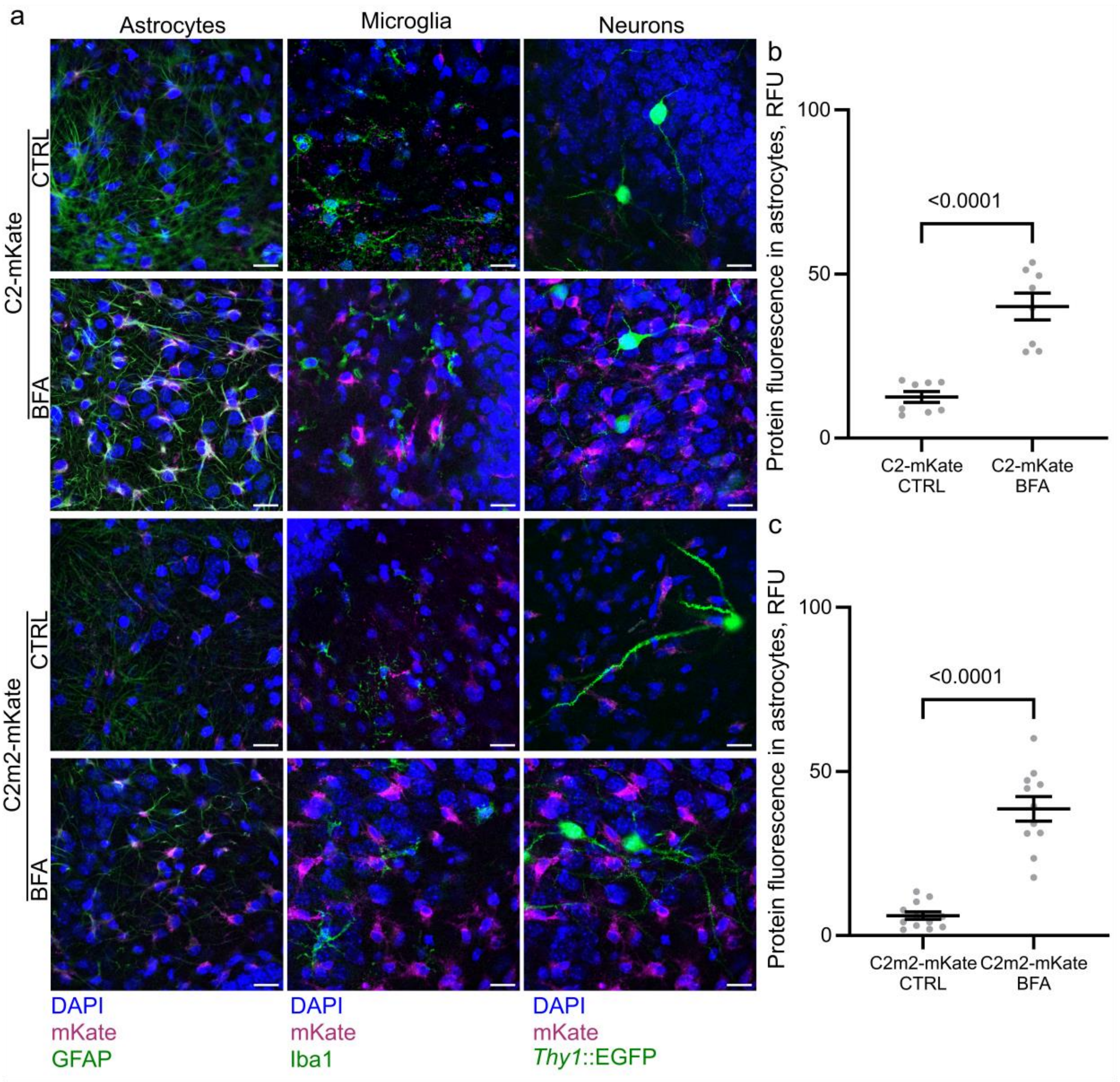

Figure S6 The expression of C2-mKate fusion proteins in *ex vivo* organotypic hippocampal slices after AAV delivery. a Confocal images of OHSC transduced with either AAV-C2-mKate or AAV-C2m2-mKate. Cell nuclei were stained with DAPI. Astrocytes, microglia and neurons were labelled with GFAP or Iba1 antibodies, or expressed EGFP, respectively. Scale bar 20 µm. **b, c** Quantification of C2-mKate and C2m2-mKate fluorescence within astrocytes with or without 10 µg/ml brefeldin A (BFA) treatment for 5 hours. Data presented as means ± standard error of the mean (n = 10-15). Means were compared by one-way ANOVA and *post-hoc* Tukey test. *p* values < 0.05 were considered as significant. RFU – relative fluorescence units.

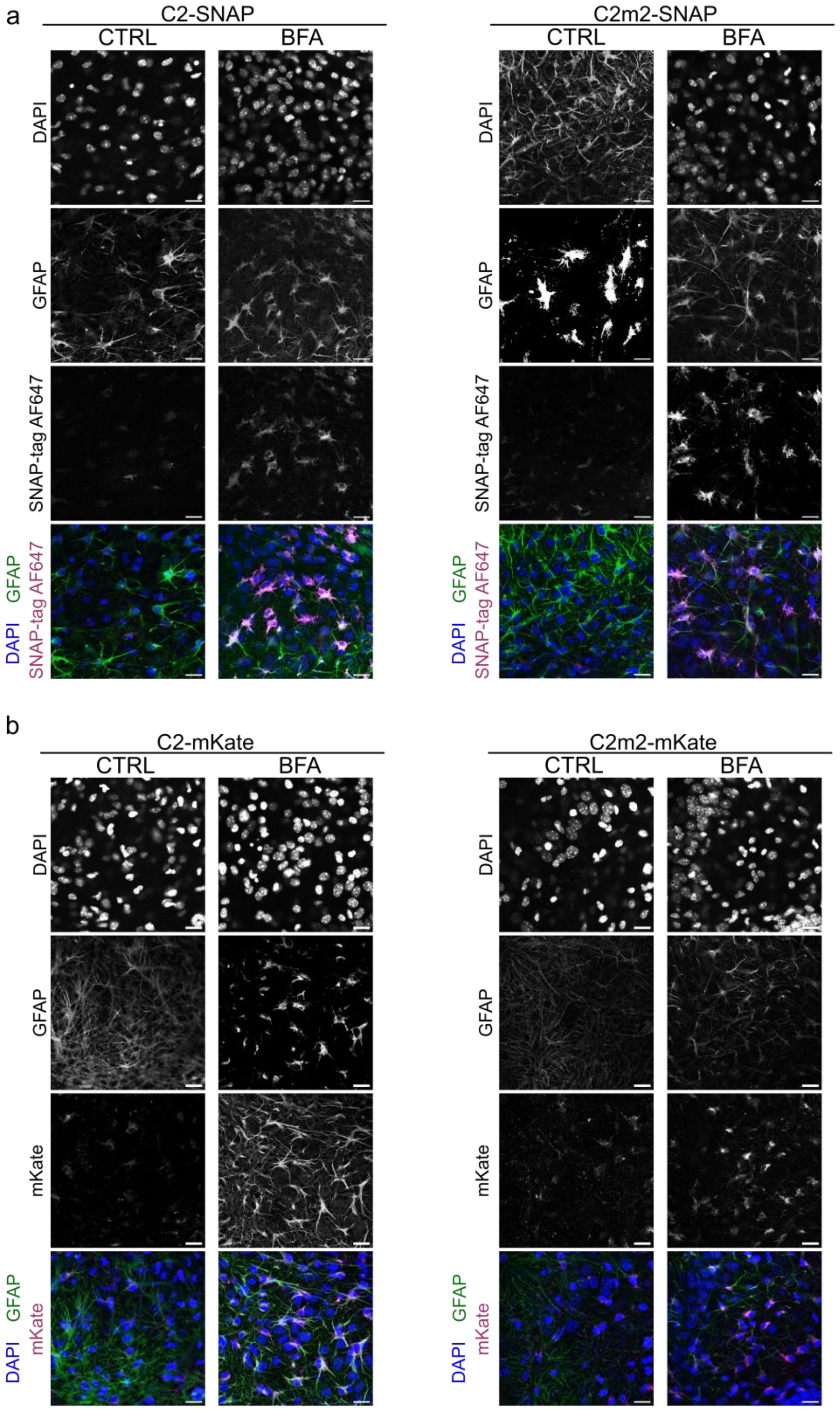

Figure S7 The expression of C2 fusion proteins in astrocytes after AAV delivery. **a** Confocal images of OHSC transduced with either AAV-C2-SNAP or AAV-C2m2-SNAP with or without 10 µg/ml brefeldin A (BFA) treatment for 5 hours. **b** Confocal images of OHSC transduced with either AAV-C2-mKate or AAV-C2m2-mKate with or without 10 µg/ml brefeldin A (BFA) treatment for 5 hours. Cell nuclei were stained with DAPI. Astrocytes were labelled with GFAP. Scale bar 20 µm.

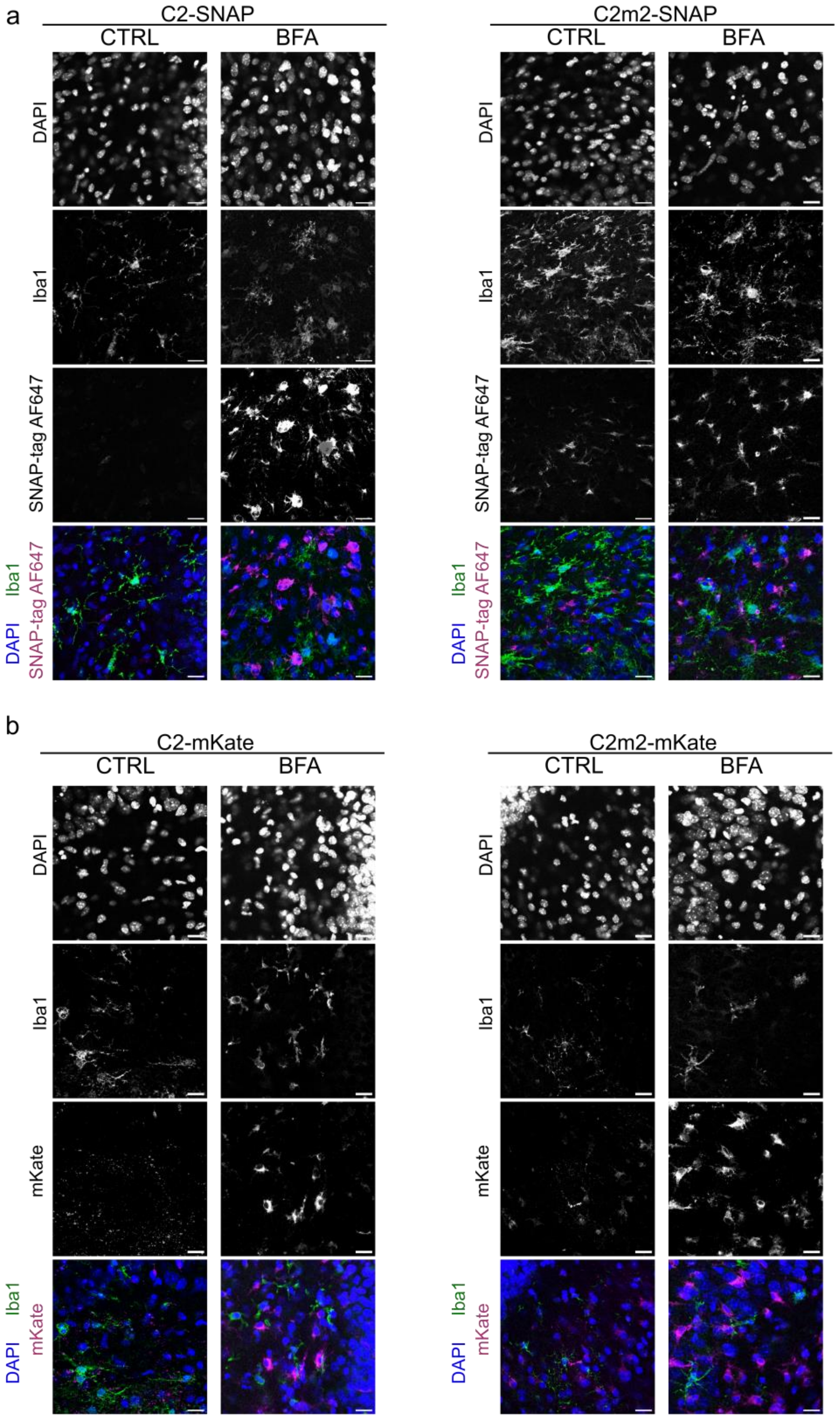

Figure S8 The expression of C2 fusion proteins in microglia after AAV delivery. a Confocal images of OHSC transduced with either AAV-C2-SNAP or AAV-C2m2-SNAP with or without 10 µg/ml brefeldin A (BFA) treatment for 5 hours. **b** Confocal images of OHSC transduced with either AAV-C2-mKate or AAV-C2m2-mKate with or without 10 µg/ml brefeldin A (BFA) treatment for 5 hours. Cell nuclei were stained with DAPI. Microglia were labelled with Iba1 antibodies. Scale bar 20 µm.

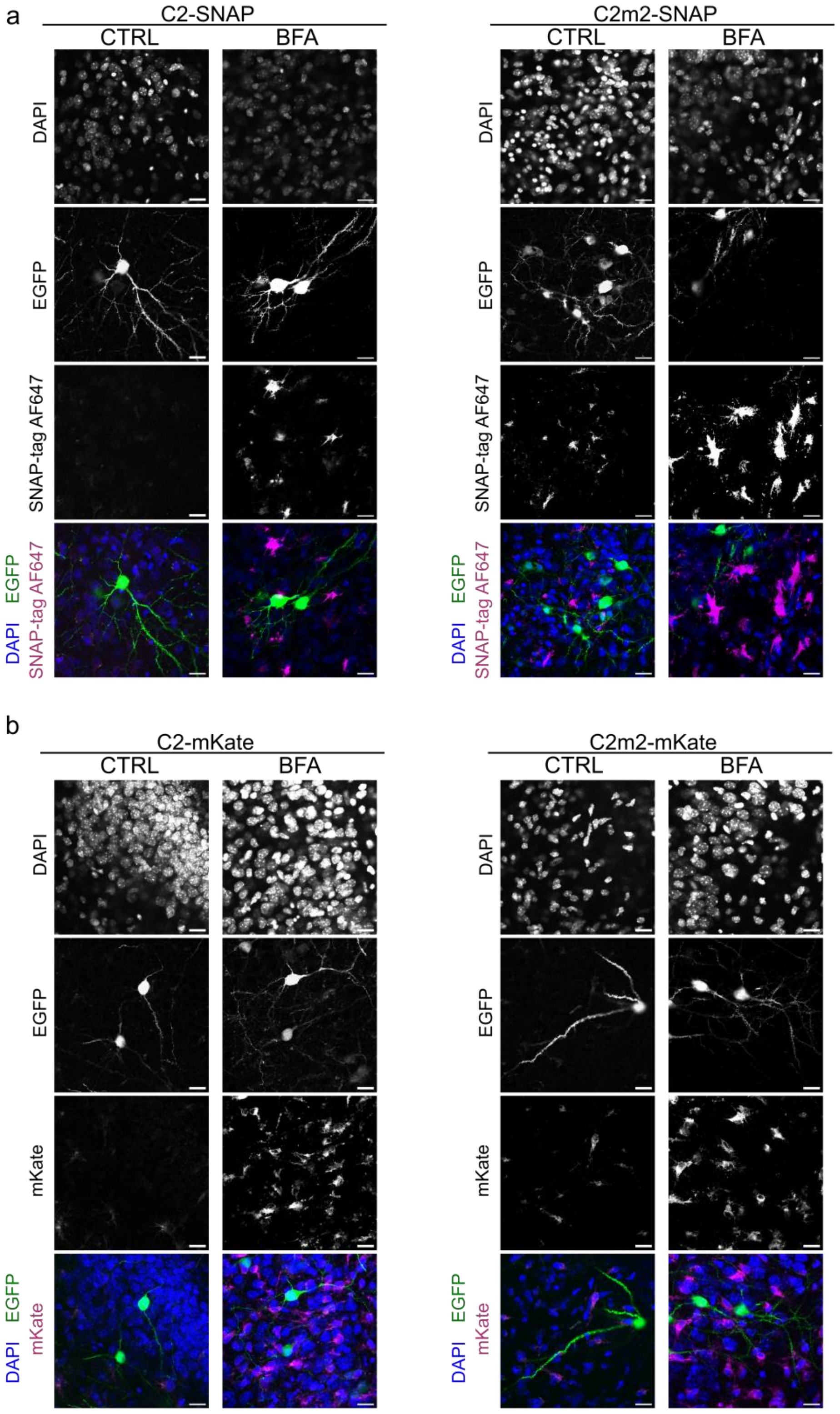

Figure S9 The expression of C2 fusion proteins in pyramidal neurons after AAV delivery. a Confocal images of OHSC transduced with either AAV-C2-SNAP or AAV-C2m2-SNAP with or without 10 µg/ml brefeldin A (BFA) treatment for 5 hours. **b** Confocal images of OHSC transduced with either AAV-C2-mKate or AAV-C2m2-mKate with or without 10 µg/ml brefeldin A (BFA) treatment for 5 hours. Cell nuclei were stained with DAPI. Neurons were expressing EGFP. Scale bar 20 µm.
