# Supplementary material for: Genetically encoded phosphatidylserine biosensor for in vitro, ex vivo and in vivo labelling
Source: Cell Mol Biol Lett. 2023 Jul 27;28:59. doi: 10.1186/s11658-023-00472-7 (PMC10373266; doi:10.1186/s11658-023-00472-7)
Supplement: Supplementary file 2 — Additional file 2. Plasmid sequences. [file 11658_2023_472_MOESM2_ESM.pdf]

Additional File 2 for

Genetically encoded phosphatidylserine biosensor for in vitro, ex vivo and in vivo labelling

Eimina Dirvelyte[1], Daina Bujanauskiene[1,2], Evelina Jankaityte[1,3], Neringa Daugelaviciene[1], Ugne Kisieliute[2], Igor Nagula[1], Rima Budvytyte[1,3], Urte Neniskyte[1,2]*

[1]VU LSC-EMBL Partnership for Genome Editing Technologies, Life Sciences Center, Vilnius University, Vilnius, Lithuania

[2]Institute of Bioscience, Life Sciences Center, Vilnius University, Vilnius, Lithuania

[3]Institute of Biochemistry, Life Sciences Center, Vilnius University, Vilnius, Lithuania

*Corresponding author: urte.neniskyte@gmc.vu.lt

This file includes:

9 plasmid sequences

Nucleotide sequences:

Sequence Name: pET21a(+)-C2

Length: 5864

CTGTCCTTCT AGTGTAGCCG TAGTTAGGCC ACCACTTCAA GAACTCTGTA GCACCGCCTA 60

CATACCTCGC TCTGCTAATC CTGTTACCAG TGGCTGCTGC CAGTGGCGAT AAGTCGTGTC 120

TTACCGGGTT GGACTCAAGA CGATAGTTAC CGGATAAGGC GCAGCGGTCG GGCTGAACGG 180

GGGGTTCGTG CACACAGCCC AGCTTGGAGC GAACGACCTA CACCGAACTG AGATACCTAC 240

AGCGTGAGCT ATGAGAAAGC GCCACGCTTC CCGAAGGGAG AAAGGCGGAC AGGTATCCGG 300

TAAGCGGCAG GGTCGGAACA GGAGAGCGCA CGAGGGAGCT TCCAGGGGGA AACGCCTGGT 360

ATCTTTATAG TCCTGTCGGG TTTCGCCACC TCTGACTTGA GCGTCGATTT TTGTGATGCT 420

CGTCAGGGGG GCGGAGCCTA TGGAAAAACG CCAGCAACGC GGCCTTTTTA CGGTTCCTGG 480

CCTTTTGCTG GCCTTTTGCT CACATGTTCT TTCCTGCGTT ATCCCCTGAT TCTGTGGATA 540

ACCGTATTAC CGCCTTTGAG TGAGCTGATA CCGCTCGCCG CAGCCGAACG ACCGAGCGCA 600

GCGAGTCAGT GAGCGAGGAA GCGGAAGAGC GCCTGATGCG GTATTTTCTC CTTACGCATC 660

TGTGCGGTAT TTCACACCGC ATATATGGTG CACTCTCAGT ACAATCTGCT CTGATGCCGC 720

ATAGTTAAGC CAGTATACAC TCCGCTATCG CTACGTGACT GGGTCATGGC TGCGCCCCGA 780

CACCCGCCAA CACCCGCTGA CGCGCCCTGA CGGGCTTGTC TGCTCCCGGC ATCCGCTTAC 840

AGACAAGCTG TGACCGTCTC CGGGAGCTGC ATGTGTCAGA GGTTTTCACC GTCATCACCG 900

AAACGCGCGA GGCAGCTGCG GTAAAGCTCA TCAGCGTGGT CGTGAAGCGA TTCACAGATG 960

TCTGCCTGTT CATCCGCGTC CAGCTCGTTG AGTTTCTCCA GAAGCGTTAA TGTCTGGCTT 1020

CTGATAAAGC GGGCCATGTT AAGGGCGGTT TTTTCCTGTT TGGTCACTGA TGCCTCCGTG 1080

TAAGGGGGAT TTCTGTTCAT GGGGGTAATG ATACCGATGA AACGAGAGAG GATGCTCACG 1140

ATACGGGTTA CTGATGATGA ACATGCCCGG TTACTGGAAC GTTGTGAGGG TAAACAACTG 1200

GCGGTATGGA TGCGGCGGGA CCAGAGAAAA ATCACTCAGG GTCAATGCCA GCGCTTCGTT 1260

AATACAGATG TAGGTGTTCC ACAGGGTAGC CAGCAGCATC CTGCGATGCA GATCCGGAAC 1320

ATAATGGTGC AGGGCGCTGA CTTCCGCGTT TCCAGACTTT ACGAAACACG GAAACCGAAG 1380

ACCATTCATG TTGTTGCTCA GGTCGCAGAC GTTTTGCAGC AGCAGTCGCT TCACGTTCGC 1440

TCGCGTATCG GTGATTCATT CTGCTAACCA GTAAGGCAAC CCCGCCAGCC TAGCCGGGTC 1500

CTCAACGACA GGAGCACGAT CATGCGCACC CGTGGGGCCG CCATGCCGGC GATAATGGCC 1560

TGCTTCTCGC CGAAACGTTT GGTGGCGGGA CCAGTGACGA AGGCTTGAGC GAGGGCGTGC 1620

AAGATTCCGA ATACCGCAAG CGACAGGCCG ATCATCGTCG CGCTCCAGCG AAAGCGGTCC 1680

TCGCCGAAAA TGACCCAGAG CGCTGCCGGC ACCTGTCCTA CGAGTTGCAT GATAAAGAAG 1740

ACAGTCATAA GTGCGGCGAC GATAGTCATG CCCCGCGCCC ACCGGAAGGA GCTGACTGGG 1800

TTGAAGGCTC TCAAGGGCAT CGGTCGAGAT CCCGGTGCCT AATGAGTGAG CTAACTTACA 1860

TTAATTGCGT TGCGCTCACT GCCCGCTTTC CAGTCGGGAA ACCTGTCGTG CCAGCTGCAT 1920

TAATGAATCG GCCAACGCGC GGGGAGAGGC GGTTTGCGTA TTGGGCGCCA GGGTGGTTTT 1980

TCTTTTCACC AGTGAGACGG GCAACAGCTG ATTGCCCTTC ACCGCCTGGC CCTGAGAGAG 2040

TTGCAGCAAG CGGTCCACGC TGGTTTGCCC CAGCAGGCGA AAATCCTGTT TGATGGTGGT 2100

TAACGGCGGG ATATAACATG AGCTGTCTTC GGTATCGTCG TATCCCACTA CCGAGATATC 2160

CGCACCAACG CGCAGCCCGG ACTCGGTAAT GGCGCGCATT GCGCCCAGCG CCATCTGATC 2220

GTTGGCAACC AGCATCGCAG TGGGAACGAT GCCCTCATTC AGCATTTGCA TGGTTTGTTG 2280

AAAACCGGAC ATGGCACTCC AGTCGCCTTC CCGTTCCGCT ATCGGCTGAA TTTGATTGCG 2340

AGTGAGATAT TTATGCCAGC CAGCCAGACG CAGACGCGCC GAGACAGAAC TTAATGGGCC 2400

CGCTAACAGC GCGATTTGCT GGTGACCCAA TGCGACCAGA TGCTCCACGC CCAGTCGCGT 2460

ACCGTCTTCA TGGGAGAAAA TAATACTGTT GATGGGTGTC TGGTCAGAGA CATCAAGAAA 2520

TAACGCCGGA ACATTAGTGC AGGCAGCTTC CACAGCAATG GCATCCTGGT CATCCAGCGG 2580

ATAGTTAATG ATCAGCCCAC TGACGCGTTG CGCGAGAAGA TTGTGCACCG CCGCTTTACA 2640

GGCTTCGACG CCGCTTCGTT CTACCATCGA CACCACCACG CTGGCACCCA GTTGATCGGC 2700

GCGAGATTTA ATCGCCGCGA CAATTTGCGA CGGCGCGTGC AGGGCCAGAC TGGAGGTGGC 2760

AACGCCAATC AGCAACGACT GTTTGCCCGC CAGTTGTTGT GCCACGCGGT TGGGAATGTA 2820

ATTCAGCTCC GCCATCGCCG CTTCCACTTT TTCCCGCGTT TTCGCAGAAA CGTGGCTGGC 2880

CTGGTTCACC ACGCGGGAAA CGGTCTGATA AGAGACACCG GCATACTCTG CGACATCGTA 2940

TAACGTTACT GGTTTCACAT TCACCACCCT GAATTGACTC TCTTCCGGGC GCTATCATGC 3000

CATACCGCGA AAGGTTTTGC GCCATTCGAT GGTGTCCGGG ATCTCGACGC TCTCCCTTAT 3060

GCGACTCCTG CATTAGGAAG CAGCCCAGTA GTAGGTTGAG GCCGTTGAGC ACCGCCGCCG 3120

CAAGGAATGG TGCATGCAAG GAGATGGCGC CCAACAGTCC CCCGGCCACG GGGCCTGCCA 3180

CCATACCCAC GCCGAAACAA GCGCTCATGA GCCCGAAGTG GCGAGCCCGA TCTTCCCCAT 3240

CGGTGATGTC GGCGATATAG GCGCCAGCAA CCGCACCTGT GGCGCCGGTG ATGCCGGCCA 3300

T7 promoter

CGATGCGTCC GGCGTAGAGG ATCGAGATCT CGATCCCGCG AAATTAATAC GACTCACTAT 3360

Lac operator **RBS**

AGGGGAATTG TGAGCGGATA ACAATTCCCC TCTAGAAATA ATTTTGTTTA ACTTTAAGAA 3420

C2 domain

GGAGATATAC ATAAGCATAT GAAAAGCGGA CACGGATGTT CTGAGCCCCT GGGCCTGAAG 3480

AATAACACAA TTCCTGACAG CCAGATGTCA GCCTCCAGCA GCTACAAGAC ATGGAACCTG 3540

CGTGCTTTTG GCTGGTACCC CCACTTGGGA AGGCTGGATA ATCAGGGCAA GATCAATGCC 3600

TGGACGGCTC AGAGCAACAG TGCCAAGGAA TGGCTGCAGG TTGACCTGGG CACTCAGAGG 3660

CAAGTGACAG GAATCATCAC CCAGGGGGCC CGTGACTTTG GCCACATCCA GTATGTGGCG 3720

TCCTACAAGG TAGCCCACAG TGATGATGGT GTGCAGTGGA CTGTATATGA GGAGCAAGGA 3780

AGCAGCAAGG TCTTCCAGGG CAACTTGGAC AACAACTCCC ACAAGAAGAA CATCTTCGAG 3840

AAACCCTTCA TGGCTCGCTA CGTGCGTGTC CTTCCAGTGT CCTGGCATAA CCGCATCACC 3900

His-tag

CTGCGCCTGG AGCTGCTGGG CTGTAAGCTT GCGGCCGCAC TCGAGCACCA CCACCACCAC 3960

CACTGAGATC CGGCTGCTAA CAAAGCCCGA AAGGAAGCTG AGTTGGCTGC TGCCACCGCT 4020

T7 term

GAGCAATAAC TAGCATAACC CCTTGGGGCC TCTAAACGGG TCTTGAGGGG TTTTTTGCTG 4080

AAAGGAGGAA CTATATCCGG ATTGGCGAAT GGGACGCGCC CTGTAGCGGC GCATTAAGCG 4140

CGGCGGGTGT GGTGGTTACG CGCAGCGTGA CCGCTACACT TGCCAGCGCC CTAGCGCCCG 4200

CTCCTTTCGC TTTCTTCCCT TCCTTTCTCG CCACGTTCGC CGGCTTTCCC CGTCAAGCTC 4260

TAAATCGGGG GCTCCCTTTA GGGTTCCGAT TTAGTGCTTT ACGGCACCTC GACCCCAAAA 4320

AACTTGATTA GGGTGATGGT TCACGTAGTG GGCCATCGCC CTGATAGACG GTTTTTCGCC 4380

CTTTGACGTT GGAGTCCACG TTCTTTAATA GTGGACTCTT GTTCCAAACT GGAACAACAC 4440

TCAACCCTAT CTCGGTCTAT TCTTTTGATT TATAAGGGAT TTTGCCGATT TCGGCCTATT 4500

GGTTAAAAAA TGAGCTGATT TAACAAAAAT TTAACGCGAA TTTTAACAAA ATATTAACGT 4560

TTACAATTTC AGGTGGCACT TTTCGGGGAA ATGTGCGCGG AACCCCTATT TGTTTATTTT 4620

TCTAAATACA TTCAAATATG TATCCGCTCA TGAGACAATA ACCCTGATAA ATGCTTCAAT 4680

AATATTGAAA AAGGAAGAGT ATGAGTATTC AACATTTCCG TGTCGCCCTT ATTCCCTTTT 4740

TTGCGGCATT TTGCCTTCCT GTTTTTGCTC ACCCAGAAAC GCTGGTGAAA GTAAAAGATG 4800

CTGAAGATCA GTTGGGTGCA CGAGTGGGTT ACATCGAACT GGATCTCAAC AGCGGTAAGA 4860

TCCTTGAGAG TTTTCGCCCC GAAGAACGTT TTCCAATGAT GAGCACTTTT AAAGTTCTGC 4920

TATGTGGCGC GGTATTATCC CGTATTGACG CCGGGCAAGA GCAACTCGGT CGCCGCATAC 4980

ACTATTCTCA GAATGACTTG GTTGAGTACT CACCAGTCAC AGAAAAGCAT CTTACGGATG 5040

GCATGACAGT AAGAGAATTA TGCAGTGCTG CCATAACCAT GAGTGATAAC ACTGCGGCCA 5100

ACTTACTTCT GACAACGATC GGAGGACCGA AGGAGCTAAC CGCTTTTTTG CACAACATGG 5160

GGGATCATGT AACTCGCCTT GATCGTTGGG AACCGGAGCT GAATGAAGCC ATACCAAACG 5220

ACGAGCGTGA CACCACGATG CCTGCAGCAA TGGCAACAAC GTTGCGCAAA CTATTAACTG 5280

GCGAACTACT TACTCTAGCT TCCCGGCAAC AATTAATAGA CTGGATGGAG GCGGATAAAG 5340

TTGCAGGACC ACTTCTGCGC TCGGCCCTTC CGGCTGGCTG GTTTATTGCT GATAAATCTG 5400

GAGCCGGTGA GCGTGGGTCT CGCGGTATCA TTGCAGCACT GGGGCCAGAT GGTAAGCCCT 5460

CCCGTATCGT AGTTATCTAC ACGACGGGGA GTCAGGCAAC TATGGATGAA CGAAATAGAC 5520

AGATCGCTGA GATAGGTGCC TCACTGATTA AGCATTGGTA ACTGTCAGAC CAAGTTTACT 5580

CATATATACT TTAGATTGAT TTAAAACTTC ATTTTTAATT TAAAAGGATC TAGGTGAAGA 5640

TCCTTTTTGA TAATCTCATG ACCAAAATCC CTTAACGTGA GTTTTCGTTC CACTGAGCGT 5700

CAGACCCCGT AGAAAAGATC AAAGGATCTT CTTGAGATCC TTTTTTTCTG CGCGTAATCT 5760

GCTGCTTGCA AACAAAAAAA CCACCGCTAC CAGCGGTGGT TTGTTTGCCG GATCAAGAGC 5820

TACCAACTCT TTTTCCGAAG GTAACTGGCT TCAGCAGAGC GCAGATACCA AATA 5864

Sequence Name: pET21a(+)-C2-mKate

Length: 6624

CTGTCCTTCT AGTGTAGCCG TAGTTAGGCC ACCACTTCAA GAACTCTGTA GCACCGCCTA 60
CATACCTCGC TCTGCTAATC CTGTTACCAG TGGCTGCTGC CAGTGGCGAT AAGTCGTGTC 120
TTACCGGGTT GGACTCAAGA CGATAGTTAC CGGATAAGGC GCAGCGGTCG GGCTGAACGG 180
GGGGTTCGTG CACACAGCCC AGCTTGGAGC GAACGACCTA CACCGAACTG AGATACCTAC 240
AGCGTGAGCT ATGAGAAAGC GCCACGCTTC CCGAAGGGAG AAAGGCGGAC AGGTATCCGG 300
TAAGCGGCAG GGTCGGAACA GGAGAGCGCA CGAGGGAGCT TCCAGGGGGA AACGCCTGGT 360
ATCTTTATAG TCCTGTCGGG TTTCGCCACC TCTGACTTGA GCGTCGATTT TTGTGATGCT 420
CGTCAGGGGG GCGGAGCCTA TGGAAAAACG CCAGCAACGC GGCCTTTTTA CGGTTCCTGG 480
CCTTTTGCTG GCCTTTTGCT CACATGTTCT TTCCTGCGTT ATCCCCTGAT TCTGTGGATA 540
ACCGTATTAC CGCCTTTGAG TGAGCTGATA CCGCTCGCCG CAGCCGAACG ACCGAGCGCA 600
GCGAGTCAGT GAGCGAGGAA GCGGAAGAGC GCCTGATGCG GTATTTTCTC CTTACGCATC 660
TGTGCGGTAT TTCACACCGC ATATATGGTG CACTCTCAGT ACAATCTGCT CTGATGCCGC 720
ATAGTTAAGC CAGTATACAC TCCGCTATCG CTACGTGACT GGGTCATGGC TGCGCCCCGA 780
CACCCGCCAA CACCCGCTGA CGCGCCCTGA CGGGCTTGTC TGCTCCCGGC ATCCGCTTAC 840
AGACAAGCTG TGACCGTCTC CGGGAGCTGC ATGTGTCAGA GGTTTTCACC GTCATCACCG 900
AAACGCGCGA GGCAGCTGCG GTAAAGCTCA TCAGCGTGGT CGTGAAGCGA TTCACAGATG 960
TCTGCCTGTT CATCCGCGTC CAGCTCGTTG AGTTTCTCCA GAAGCGTTAA TGTCTGGCTT 1020
CTGATAAAGC GGGCCATGTT AAGGGCGGTT TTTTCCTGTT TGGTCACTGA TGCCTCCGTG 1080
TAAGGGGGAT TTCTGTTCAT GGGGGTAATG ATACCGATGA AACGAGAGAG GATGCTCACG 1140
ATACGGGTTA CTGATGATGA ACATGCCCGG TTACTGGAAC GTTGTGAGGG TAAACAACTG 1200
GCGGTATGGA TGCGGCGGGA CCAGAGAAAA ATCACTCAGG GTCAATGCCA GCGCTTCGTT 1260
AATACAGATG TAGGTGTTCC ACAGGGTAGC CAGCAGCATC CTGCGATGCA GATCCGGAAC 1320
ATAATGGTGC AGGGCGCTGA CTTCCGCGTT TCCAGACTTT ACGAAACACG GAAACCGAAG 1380
ACCATTCATG TTGTTGCTCA GGTCGCAGAC GTTTTGCAGC AGCAGTCGCT TCACGTTCGC 1440
TCGCGTATCG GTGATTCATT CTGCTAACCA GTAAGGCAAC CCCGCCAGCC TAGCCGGGTC 1500
CTCAACGACA GGAGCACGAT CATGCGCACC CGTGGGGCCG CCATGCCGGC GATAATGGCC 1560
TGCTTCTCGC CGAAACGTTT GGTGGCGGGA CCAGTGACGA AGGCTTGAGC GAGGGCGTGC 1620
AAGATTCCGA ATACCGCAAG CGACAGGCCG ATCATCGTCG CGCTCCAGCG AAAGCGGTCC 1680
TCGCCGAAAA TGACCCAGAG CGCTGCCGGC ACCTGTCCTA CGAGTTGCAT GATAAAGAAG 1740
ACAGTCATAA GTGCGGCGAC GATAGTCATG CCCCGCGCCC ACCGGAAGGA GCTGACTGGG 1800
TTGAAGGCTC TCAAGGGCAT CGGTCGAGAT CCCGGTGCCT AATGAGTGAG CTAACTTACA 1860
TTAATTGCGT TGCGCTCACT GCCCGCTTTC CAGTCGGGAA ACCTGTCGTG CCAGCTGCAT 1920
TAATGAATCG GCCAACGCGC GGGGAGAGGC GGTTTGCGTA TTGGGCGCCA GGGTGGTTTT 1980
TCTTTTCACC AGTGAGACGG GCAACAGCTG ATTGCCCTTC ACCGCCTGGC CCTGAGAGAG 2040

TTGCAGCAAG CGGTCCACGC TGGTTTGCCC CAGCAGGCGA AAATCCTGTT TGATGGTGGT 2100

TAACGGCGGG ATATAACATG AGCTGTCTTC GGTATCGTCG TATCCCACTA CCGAGATATC 2160

CGCACCAACG CGCAGCCCGG ACTCGGTAAT GGCGCGCATT GCGCCCAGCG CCATCTGATC 2220

GTTGGCAACC AGCATCGCAG TGGGAACGAT GCCCTCATTC AGCATTTGCA TGGTTTGTTG 2280

AAAACCGGAC ATGGCACTCC AGTCGCCTTC CCGTTCCGCT ATCGGCTGAA TTTGATTGCG 2340

AGTGAGATAT TTATGCCAGC CAGCCAGACG CAGACGCGCC GAGACAGAAC TTAATGGGCC 2400

CGCTAACAGC GCGATTTGCT GGTGACCCAA TGCGACCAGA TGCTCCACGC CCAGTCGCGT 2460

ACCGTCTTCA TGGGAGAAAA TAATACTGTT GATGGGTGTC TGGTCAGAGA CATCAAGAAA 2520

TAACGCCGGA ACATTAGTGC AGGCAGCTTC CACAGCAATG GCATCCTGGT CATCCAGCGG 2580

ATAGTTAATG ATCAGCCCAC TGACGCGTTG CGCGAGAAGA TTGTGCACCG CCGCTTTACA 2640

GGCTTCGACG CCGCTTCGTT CTACCATCGA CACCACCACG CTGGCACCCA GTTGATCGGC 2700

GCGAGATTTA ATCGCCGCGA CAATTTGCGA CGGCGCGTGC AGGGCCAGAC TGGAGGTGGC 2760

AACGCCAATC AGCAACGACT GTTTGCCCGC CAGTTGTTGT GCCACGCGGT TGGGAATGTA 2820

ATTCAGCTCC GCCATCGCCG CTTCCACTTT TTCCCGCGTT TTCGCAGAAA CGTGGCTGGC 2880

CTGGTTCACC ACGCGGGAAA CGGTCTGATA AGAGACACCG GCATACTCTG CGACATCGTA 2940

TAACGTTACT GGTTTCACAT TCACCACCCT GAATTGACTC TCTTCCGGGC GCTATCATGC 3000

CATACCGCGA AAGGTTTTGC GCCATTCGAT GGTGTCCGGG ATCTCGACGC TCTCCCTTAT 3060

GCGACTCCTG CATTAGGAAG CAGCCCAGTA GTAGGTTGAG GCCGTTGAGC ACCGCCGCCG 3120

CAAGGAATGG TGCATGCAAG GAGATGGCGC CCAACAGTCC CCCGGCCACG GGGCCTGCCA 3180

CCATACCCAC GCCGAAACAA GCGCTCATGA GCCCGAAGTG GCGAGCCCGA TCTTCCCCAT 3240

CGGTGATGTC GGCGATATAG GCGCCAGCAA CCGCACCTGT GGCGCCGGTG ATGCCGGCCA 3300

T7 promoter

CGATGCGTCC GGCGTAGAGG ATCGAGATCT CGATCCCGCG AAATTAATAC GACTCACTAT 3360

Lac operator **RBS**

AGGGGAATTG TGAGCGGATA ACAATTCCCC TCTAGAAATA ATTTTGTTTA ACTTTAAGAA 3420

C2 domain

GGAGATATAC ATATGCACGG ATGTTCTGAG CCCCTGGGCC TGAAGAATAA CACAATTCCT 3480

GACAGCCAGA TGTCAGCCTC CAGCAGCTAC AAGACATGGA ACCTGCGTGC TTTTGGCTGG 3540

TACCCCCACT TGGGAAGGCT GGATAATCAG GGCAAGATCA ATGCCTGGAC GGCTCAGAGC 3600

AACAGTGCCA AGGAATGGCT GCAGGTTGAC CTGGGCACTC AGAGGCAAGT GACAGGAATC 3660

ATCACCCAGG GGGCCCGTGA CTTTGGCCAC ATCCAGTATG TGGCGTCCTA CAAGGTAGCC 3720

CACAGTGATG ATGGTGTGCA GTGGACTGTA TATGAGGAGC AAGGAAGCAG CAAGGTCTTC 3780

CAGGGCAACT TGGACAACAA CTCCCACAAG AAGAACATCT TCGAGAAACC CTTCATGGCT 3840

CGCTACGTGC GTGTCCTTCC AGTGTCCTGG CATAACCGCA TCACCCTGCG CCTGGAGCTG 3900

linker

CTGGGCTGTA AGCTTGGCAC TGGAGGCTCT GGAGGCACTG GAGGCTCTGG AGGCACCGGT 3960

mKate

AGCACTAGCG GCGGAAGCGG CGGGACAGGT ACGCGTGTGA GCGAGCTGAT TAAGGAGAAC 4020

ATGCACATGA AGCTGTACAT GGAGGGCACC GTGAACAACC ACCACTTCAA GTGCACATCC 4080

GAGGGCGAAG GCAAGCCCTA CGAGGGCACC CAGACCATGA GAATCAAGGT CGTCGAGGGC 4140

GGCCCTCTCC CCTTCGCCTT CGACATCCTG GCTACCAGCT TCATGTACGG CAGCAAAACC 4200

TTCATCAACC ACCCTCAGGG CATCCCCGAC TTCTTTAAGC AGTCCTTCCC TGAGGGCTTC 4260

ACATGGGAGA GAGTCACCAC ATACGAAGAC GGGGGCGTGC TGACCGCTAC CCAGGACACC 4320

AGCCTCCAGG ACGGCTGCCT CATCTACAAC GTCAAGATTA GAGGGGTGAA CTTCCCAGCC 4380

AACGGCCCTG TGATGCAGAA GAAAACACTC GGCTGGGAGG CCTCCACCGA GACGCTGTAC 4440

CCCGCTGACG GCGGCCTGGA AGGCGCATGT GACATGGCCC TGAAGCTCGT GGGCGGGGGC 4500

CACCTGATCT GCAACTTGGA GACCACATAC AGATCCAAGA AACCCGCTAA GAACCTCAAG 4560

ATGCCCGGCG TCTACAACGT GGACAGGAGA CTGGAAAGAA TCAAGGAGGC CGACAATGAG 4620

ACCTACGTCG AGCAGCACGA GGTGGCTGTG GCCAGATACT CTACTGGTGG CGCTGGTGAT 4680

His-tag

GGAGGTAAAC TCGAGCACCA CCACCACCAC CACTGAGATC CGGCTGCTAA CAAAGCCCGA 4740

T7 term

AAGGAAGCTG AGTTGGCTGC TGCCACCGCT GAGCAATAAC TAGCATAACC CCTTGGGGCC 4800

TCTAAACGGG TCTTGAGGGG TTTTTTGCTG AAAGGAGGAA CTATATCCGG ATTGGCGAAT 4860

GGGACGCGCC CTGTAGCGGC GCATTAAGCG CGGCGGGTGT GGTGGTTACG CGCAGCGTGA 4920

CCGCTACACT TGCCAGCGCC CTAGCGCCCG CTCCTTTCGC TTTCTTCCCT TCCTTTCTCG 4980

CCACGTTCGC CGGCTTTCCC CGTCAAGCTC TAAATCGGGG GCTCCCTTTA GGGTTCCGAT 5040

TTAGTGCTTT ACGGCACCTC GACCCCAAAA AACTTGATTA GGGTGATGGT TCACGTAGTG 5100

GGCCATCGCC CTGATAGACG GTTTTTCGCC CTTTGACGTT GGAGTCCACG TTCTTTAATA 5160

GTGGACTCTT GTTCCAAACT GGAACAACAC TCAACCCTAT CTCGGTCTAT TCTTTTGATT 5220

TATAAGGGAT TTTGCCGATT TCGGCCTATT GGTTAAAAAA TGAGCTGATT TAACAAAAAT 5280

TTAACGCGAA TTTTAACAAA ATATTAACGT TTACAATTTC AGGTGGCACT TTTCGGGGAA 5340

ATGTGCGCGG AACCCCTATT TGTTTATTTT TCTAAATACA TTCAAATATG TATCCGCTCA 5400

TGAGACAATA ACCCTGATAA ATGCTTCAAT AATATTGAAA AAGGAAGAGT ATGAGTATTC 5460

AACATTTCCG TGTCGCCCTT ATTCCCTTTT TTGCGGCATT TTGCCTTCCT GTTTTTGCTC 5520

ACCCAGAAAC GCTGGTGAAA GTAAAAGATG CTGAAGATCA GTTGGGTGCA CGAGTGGGTT 5580

ACATCGAACT GGATCTCAAC AGCGGTAAGA TCCTTGAGAG TTTTCGCCCC GAAGAACGTT 5640

TTCCAATGAT GAGCACTTTT AAAGTTCTGC TATGTGGCGC GGTATTATCC CGTATTGACG 5700

CCGGGCAAGA GCAACTCGGT CGCCGCATAC ACTATTCTCA GAATGACTTG GTTGAGTACT 5760

CACCAGTCAC AGAAAAGCAT CTTACGGATG GCATGACAGT AAGAGAATTA TGCAGTGCTG 5820

CCATAACCAT GAGTGATAAC ACTGCGGCCA ACTTACTTCT GACAACGATC GGAGGACCGA 5880

AGGAGCTAAC CGCTTTTTTG CACAACATGG GGGATCATGT AACTCGCCTT GATCGTTGGG 5940

AACCGGAGCT GAATGAAGCC ATACCAAACG ACGAGCGTGA CACCACGATG CCTGCAGCAA 6000
TGGCAACAAC GTTGCGCAAA CTATTAACTG GCGAACTACT TACTCTAGCT TCCCGGCAAC 6060
AATTAATAGA CTGGATGGAG GCGGATAAAG TTGCAGGACC ACTTCTGCGC TCGGCCCTTC 6120
CGGCTGGCTG GTTTATTGCT GATAAATCTG GAGCCGGTGA GCGTGGGTCT CGCGGTATCA 6180
TTGCAGCACT GGGGCCAGAT GGTAAGCCCT CCCGTATCGT AGTTATCTAC ACGACGGGGA 6240
GTCAGGCAAC TATGGATGAA CGAAATAGAC AGATCGCTGA GATAGGTGCC TCACTGATTA 6300
AGCATTGGTA ACTGTCAGAC CAAGTTTACT CATATATACT TTAGATTGAT TTAAAACTTC 6360
ATTTTTAATT TAAAAGGATC TAGGTGAAGA TCCTTTTTGA TAATCTCATG ACCAAAATCC 6420
CTTAACGTGA GTTTTCGTTC CACTGAGCGT CAGACCCCGT AGAAAAGATC AAAGGATCTT 6480
CTTGAGATCC TTTTTTTCTG CGCGTAATCT GCTGCTTGCA AACAAAAAAA CCACCGCTAC 6540
CAGCGGTGGT TTGTTTGCCG GATCAAGAGC TACCAACTCT TTTTCCGAAG GTAACTGGCT 6600
TCAGCAGAGC GCAGATACCA AATA 6624

Sequence Name: pET21a(+)-C2m2-mKate

Length: 6624

CTGTCCTTCT AGTGTAGCCG TAGTTAGGCC ACCACTTCAA GAACTCTGTA GCACCGCCTA 60
CATACCTCGC TCTGCTAATC CTGTTACCAG TGGCTGCTGC CAGTGGCGAT AAGTCGTGTC 120
TTACCGGGTT GGACTCAAGA CGATAGTTAC CGGATAAGGC GCAGCGGTCG GGCTGAACGG 180
GGGGTTCGTG CACACAGCCC AGCTTGGAGC GAACGACCTA CACCGAACTG AGATACCTAC 240
AGCGTGAGCT ATGAGAAAGC GCCACGCTTC CCGAAGGGAG AAAGGCGGAC AGGTATCCGG 300
TAAGCGGCAG GGTCGGAACA GGAGAGCGCA CGAGGGAGCT TCCAGGGGGA AACGCCTGGT 360
ATCTTTATAG TCCTGTCGGG TTTCGCCACC TCTGACTTGA GCGTCGATTT TTGTGATGCT 420
CGTCAGGGGG GCGGAGCCTA TGGAAAAACG CCAGCAACGC GGCCTTTTTA CGGTTCCTGG 480
CCTTTTGCTG GCCTTTTGCT CACATGTTCT TTCCTGCGTT ATCCCCTGAT TCTGTGGATA 540
ACCGTATTAC CGCCTTTGAG TGAGCTGATA CCGCTCGCCG CAGCCGAACG ACCGAGCGCA 600
GCGAGTCAGT GAGCGAGGAA GCGGAAGAGC GCCTGATGCG GTATTTTCTC CTTACGCATC 660
TGTGCGGTAT TTCACACCGC ATATATGGTG CACTCTCAGT ACAATCTGCT CTGATGCCGC 720
ATAGTTAAGC CAGTATACAC TCCGCTATCG CTACGTGACT GGGTCATGGC TGCGCCCCGA 780
CACCCGCCAA CACCCGCTGA CGCGCCCTGA CGGGCTTGTC TGCTCCCGGC ATCCGCTTAC 840
AGACAAGCTG TGACCGTCTC CGGGAGCTGC ATGTGTCAGA GGTTTTCACC GTCATCACCG 900
AAACGCGCGA GGCAGCTGCG GTAAAGCTCA TCAGCGTGGT CGTGAAGCGA TTCACAGATG 960
TCTGCCTGTT CATCCGCGTC CAGCTCGTTG AGTTTCTCCA GAAGCGTTAA TGTCTGGCTT 1020
CTGATAAAGC GGGCCATGTT AAGGGCGGTT TTTTCCTGTT TGGTCACTGA TGCCTCCGTG 1080
TAAGGGGGAT TTCTGTTCAT GGGGGTAATG ATACCGATGA AACGAGAGAG GATGCTCACG 1140
ATACGGGTTA CTGATGATGA ACATGCCCGG TTACTGGAAC GTTGTGAGGG TAAACAACTG 1200
GCGGTATGGA TGCGGCGGGA CCAGAGAAAA ATCACTCAGG GTCAATGCCA GCGCTTCGTT 1260

AATACAGATG TAGGTGTTCC ACAGGGTAGC CAGCAGCATC CTGCGATGCA GATCCGGAAC 1320

ATAATGGTGC AGGGCGCTGA CTTCCGCGTT TCCAGACTTT ACGAAACACG GAAACCGAAG 1380

ACCATTCATG TTGTTGCTCA GGTCGCAGAC GTTTTGCAGC AGCAGTCGCT TCACGTTCGC 1440

TCGCGTATCG GTGATTCATT CTGCTAACCA GTAAGGCAAC CCCGCCAGCC TAGCCGGGTC 1500

CTCAACGACA GGAGCACGAT CATGCGCACC CGTGGGGCCG CCATGCCGGC GATAATGGCC 1560

TGCTTCTCGC CGAAACGTTT GGTGGCGGGA CCAGTGACGA AGGCTTGAGC GAGGGCGTGC 1620

AAGATTCCGA ATACCGCAAG CGACAGGCCG ATCATCGTCG CGCTCCAGCG AAAGCGGTCC 1680

TCGCCGAAAA TGACCCAGAG CGCTGCCGGC ACCTGTCCTA CGAGTTGCAT GATAAAGAAG 1740

ACAGTCATAA GTGCGGCGAC GATAGTCATG CCCCGCGCCC ACCGGAAGGA GCTGACTGGG 1800

TTGAAGGCTC TCAAGGGCAT CGGTCGAGAT CCCGGTGCCT AATGAGTGAG CTAACTTACA 1860

TTAATTGCGT TGCGCTCACT GCCCGCTTTC CAGTCGGGAA ACCTGTCGTG CCAGCTGCAT 1920

TAATGAATCG GCCAACGCGC GGGGAGAGGC GGTTTGCGTA TTGGGCGCCA GGGTGGTTTT 1980

TCTTTTCACC AGTGAGACGG GCAACAGCTG ATTGCCCTTC ACCGCCTGGC CCTGAGAGAG 2040

TTGCAGCAAG CGGTCCACGC TGGTTTGCCC CAGCAGGCGA AAATCCTGTT TGATGGTGGT 2100

TAACGGCGGG ATATAACATG AGCTGTCTTC GGTATCGTCG TATCCCACTA CCGAGATATC 2160

CGCACCAACG CGCAGCCCGG ACTCGGTAAT GGCGCGCATT GCGCCCAGCG CCATCTGATC 2220

GTTGGCAACC AGCATCGCAG TGGGAACGAT GCCCTCATTC AGCATTTGCA TGGTTTGTTG 2280

AAAACCGGAC ATGGCACTCC AGTCGCCTTC CCGTTCCGCT ATCGGCTGAA TTTGATTGCG 2340

AGTGAGATAT TTATGCCAGC CAGCCAGACG CAGACGCGCC GAGACAGAAC TTAATGGGCC 2400

CGCTAACAGC GCGATTTGCT GGTGACCCAA TGCGACCAGA TGCTCCACGC CCAGTCGCGT 2460

ACCGTCTTCA TGGGAGAAAA TAATACTGTT GATGGGTGTC TGGTCAGAGA CATCAAGAAA 2520

TAACGCCGGA ACATTAGTGC AGGCAGCTTC CACAGCAATG GCATCCTGGT CATCCAGCGG 2580

ATAGTTAATG ATCAGCCCAC TGACGCGTTG CGCGAGAAGA TTGTGCACCG CCGCTTTACA 2640

GGCTTCGACG CCGCTTCGTT CTACCATCGA CACCACCACG CTGGCACCCA GTTGATCGGC 2700

GCGAGATTTA ATCGCCGCGA CAATTTGCGA CGGCGCGTGC AGGGCCAGAC TGGAGGTGGC 2760

AACGCCAATC AGCAACGACT GTTTGCCCGC CAGTTGTTGT GCCACGCGGT TGGGAATGTA 2820

ATTCAGCTCC GCCATCGCCG CTTCCACTTT TTCCCGCGTT TTCGCAGAAA CGTGGCTGGC 2880

CTGGTTCACC ACGCGGGAAA CGGTCTGATA AGAGACACCG GCATACTCTG CGACATCGTA 2940

TAACGTTACT GGTTTCACAT TCACCACCCT GAATTGACTC TCTTCCGGGC GCTATCATGC 3000

CATACCGCGA AAGGTTTTGC GCCATTCGAT GGTGTCCGGG ATCTCGACGC TCTCCCTTAT 3060

GCGACTCCTG CATTAGGAAG CAGCCCAGTA GTAGGTTGAG GCCGTTGAGC ACCGCCGCCG 3120

CAAGGAATGG TGCATGCAAG GAGATGGCGC CCAACAGTCC CCCGGCCACG GGGCCTGCCA 3180

CCATACCCAC GCCGAAACAA GCGCTCATGA GCCCGAAGTG GCGAGCCCGA TCTTCCCCAT 3240

CGGTGATGTC GGCGATATAG GCGCCAGCAA CCGCACCTGT GGCGCCGGTG ATGCCGGCCA 3300

T7 promoter

CGATGCGTCC GGCGTAGAGG ATCGAGATCT CGATCCCGCG AAATTAATAC GACTCACTAT 3360

Lac operator

AGGGGAATTG TGAGCGGATA ACAATTCCCC TCTAGAAATA ATTTTGTTTA ACTTTAAGAA 3420

C2 domain

GGAGATATAC ATATGCACGG ATGTTCTGAG CCCCTGGGCC TGAAGAATAA CACAATTCCT 3480

GACAGCCAGA TGTCAGCCTC CAGCAGCTAC AATACATGGA ACCTGCGTGC TTTTGGCTGG 3540

TACCCCCACT TGGGAAGGCT GGATAATCAG GGCAATATCA ATGCCTGGAC GGCTCAGAGC 3600

AACAGTGCCA AGGAATGGCT GCAGGTTGAC CTGGGCACTC AGAGGCAAGT GACAGGAATC 3660

ATCACCCAGG GGGCCCGTGA CTTTGGCCAC ATCCAGTATG TGGCGTCCTA CAAGGTAGCC 3720

CACAGTGATG ATGGTGTGCA GTGGACTGTA TATGAGGAGC AAGGAAGCAG CAAGGTCTTC 3780

CAGGGCAACT TGGACAACAA CTCCCACAAG AAGAACATCT TCGAGAAACC CTTCATGGCT 3840

CGCTACGTGC GTGTCCTTCC AGTGTCCTGG CATAACCGCA TCACCCTGCG CCTGGAGCTG 3900

linker

CTGGGCTGTA AGCTTGGCAC TGGAGGCTCT GGAGGCACTG GAGGCTCTGG AGGCACCGGT 3960

mKate

AGCACTAGCG GCGGAAGCGG CGGGACAGGT ACGCGTGTGA GCGAGCTGAT TAAGGAGAAC 4020

ATGCACATGA AGCTGTACAT GGAGGGCACC GTGAACAACC ACCACTTCAA GTGCACATCC 4080

GAGGGCGAAG GCAAGCCCTA CGAGGGCACC CAGACCATGA GAATCAAGGT CGTCGAGGGC 4140

GGCCCTCTCC CCTTCGCCTT CGACATCCTG GCTACCAGCT TCATGTACGG CAGCAAAACC 4200

TTCATCAACC ACCCTCAGGG CATCCCCGAC TTCTTTAAGC AGTCCTTCCC TGAGGGCTTC 4260

ACATGGGAGA GAGTCACCAC ATACGAAGAC GGGGGCGTGC TGACCGCTAC CCAGGACACC 4320

AGCCTCCAGG ACGGCTGCCT CATCTACAAC GTCAAGATTA GAGGGGTGAA CTTCCCAGCC 4380

AACGGCCCTG TGATGCAGAA GAAAACACTC GGCTGGGAGG CCTCCACCGA GACGCTGTAC 4440

CCCGCTGACG GCGGCCTGGA AGGCGCATGT GACATGGCCC TGAAGCTCGT GGGCGGGGGC 4500

CACCTGATCT GCAACTTGGA GACCACATAC AGATCCAAGA AACCCGCTAA GAACCTCAAG 4560

ATGCCCGGCG TCTACAACGT GGACAGGAGA CTGGAAAGAA TCAAGGAGGC CGACAATGAG 4620

ACCTACGTCG AGCAGCACGA GGTGGCTGTG GCCAGATACT CTACTGGTGG CGCTGGTGAT 4680

His-tag

GGAGGTAAAC TCGAGCACCA CCACCACCAC CACTGAGATC CGGCTGCTAA CAAAGCCCGA 4740

T7 term

AAGGAAGCTG AGTTGGCTGC TGCCACCGCT GAGCAATAAC TAGCATAACC CCTTGGGGCC 4800

TCTAAACGGG TCTTGAGGGG TTTTTTGCTG AAAGGAGGAA CTATATCCGG ATTGGCGAAT 4860

GGGACGCGCC CTGTAGCGGC GCATTAAGCG CGGCGGGTGT GGTGGTTACG CGCAGCGTGA 4920

CCGCTACACT TGCCAGCGCC CTAGCGCCCG CTCCTTTCGC TTTCTTCCCT TCCTTTCTCG 4980

CCACGTTCGC CGGCTTTCCC CGTCAAGCTC TAAATCGGGG GCTCCCTTTA GGGTTCCGAT 5040

TTAGTGCTTT ACGGCACCTC GACCCCAAAA AACTTGATTA GGGTGATGGT TCACGTAGTG 5100

GGCCATCGCC CTGATAGACG GTTTTTCGCC CTTTGACGTT GGAGTCCACG TTCTTTAATA 5160

GTGGACTCTT GTTCCAAACT GGAACAACAC TCAACCCTAT CTCGGTCTAT TCTTTTGATT 5220

TATAAGGGAT TTTGCCGATT TCGGCCTATT GGTTAAAAAA TGAGCTGATT TAACAAAAAT 5280

TTAACGCGAA TTTTAACAAA ATATTAACGT TTACAATTTC AGGTGGCACT TTTCGGGGAA 5340

ATGTGCGCGG AACCCCTATT TGTTTATTTT TCTAAATACA TTCAAATATG TATCCGCTCA 5400

TGAGACAATA ACCCTGATAA ATGCTTCAAT AATATTGAAA AAGGAAGAGT ATGAGTATTC 5460

AACATTTCCG TGTCGCCCTT ATTCCCTTTT TTGCGGCATT TTGCCTTCCT GTTTTTGCTC 5520

ACCCAGAAAC GCTGGTGAAA GTAAAAGATG CTGAAGATCA GTTGGGTGCA CGAGTGGGTT 5580

ACATCGAACT GGATCTCAAC AGCGGTAAGA TCCTTGAGAG TTTTCGCCCC GAAGAACGTT 5640

TTCCAATGAT GAGCACTTTT AAAGTTCTGC TATGTGGCGC GGTATTATCC CGTATTGACG 5700

CCGGGCAAGA GCAACTCGGT CGCCGCATAC ACTATTCTCA GAATGACTTG GTTGAGTACT 5760

CACCAGTCAC AGAAAAGCAT CTTACGGATG GCATGACAGT AAGAGAATTA TGCAGTGCTG 5820

CCATAACCAT GAGTGATAAC ACTGCGGCCA ACTTACTTCT GACAACGATC GGAGGACCGA 5880

AGGAGCTAAC CGCTTTTTTG CACAACATGG GGGATCATGT AACTCGCCTT GATCGTTGGG 5940

AACCGGAGCT GAATGAAGCC ATACCAAACG ACGAGCGTGA CACCACGATG CCTGCAGCAA 6000

TGGCAACAAC GTTGCGCAAA CTATTAACTG GCGAACTACT TACTCTAGCT TCCCGGCAAC 6060

AATTAATAGA CTGGATGGAG GCGGATAAAG TTGCAGGACC ACTTCTGCGC TCGGCCCTTC 6120

CGGCTGGCTG GTTTATTGCT GATAAATCTG GAGCCGGTGA GCGTGGGTCT CGCGGTATCA 6180

TTGCAGCACT GGGGCCAGAT GGTAAGCCCT CCCGTATCGT AGTTATCTAC ACGACGGGGA 6240

GTCAGGCAAC TATGGATGAA CGAAATAGAC AGATCGCTGA GATAGGTGCC TCACTGATTA 6300

AGCATTGGTA ACTGTCAGAC CAAGTTTACT CATATATACT TTAGATTGAT TTAAAACTTC 6360

ATTTTTAATT TAAAAGGATC TAGGTGAAGA TCCTTTTTGA TAATCTCATG ACCAAAATCC 6420

CTTAACGTGA GTTTTCGTTC CACTGAGCGT CAGACCCCGT AGAAAAGATC AAAGGATCTT 6480

CTTGAGATCC TTTTTTTCTG CGCGTAATCT GCTGCTTGCA AACAAAAAAA CCACCGCTAC 6540

CAGCGGTGGT TTGTTTGCCG GATCAAGAGC TACCAACTCT TTTTCCGAAG GTAACTGGCT 6600

TCAGCAGAGC GCAGATACCA AATA 6624

Sequence Name: pET21a(+)-C2-SNAP

Length: 6474

TGGCGAATGG GACGCGCCCT GTAGCGGCGC ATTAAGCGCG GCGGGTGTGG TGGTTACGCG 60
CAGCGTGACC GCTACACTTG CCAGCGCCCT AGCGCCCGCT CCTTTCGCTT TCTTCCCTTC 120
CTTTCTCGCC ACGTTCGCCG GCTTTCCCCG TCAAGCTCTA AATCGGGGGC TCCCTTTAGG 180
GTTCCGATTT AGTGCTTTAC GGCACCTCGA CCCCAAAAAA CTTGATTAGG GTGATGGTTC 240
ACGTAGTGGG CCATCGCCCT GATAGACGGT TTTTCGCCCT TTGACGTTGG AGTCCACGTT 300
CTTTAATAGT GGACTCTTGT TCCAAACTGG AACAACACTC AACCCTATCT CGGTCTATTC 360
TTTTGATTTA TAAGGGATTT TGCCGATTTC GGCCTATTGG TTAAAAAATG AGCTGATTTA 420
ACAAAAATTT AACGCGAATT TTAACAAAAT ATTAACGTTT ACAATTTCAG GTGGCACTTT 480
TCGGGGAAAT GTGCGCGGAA CCCCTATTTG TTTATTTTTC TAAATACATT CAAATATGTA 540
TCCGCTCATG AGACAATAAC CCTGATAAAT GCTTCAATAA TATTGAAAAA GGAAGAGTAT 600
GAGTATTCAA CATTTCCGTG TCGCCCTTAT TCCCTTTTTT GCGGCATTTT GCCTTCCTGT 660
TTTTGCTCAC CCAGAAACGC TGGTGAAAGT AAAAGATGCT GAAGATCAGT TGGGTGCACG 720
AGTGGGTTAC ATCGAACTGG ATCTCAACAG CGGTAAGATC CTTGAGAGTT TTCGCCCCGA 780
AGAACGTTTT CCAATGATGA GCACTTTTAA AGTTCTGCTA TGTGGCGCGG TATTATCCCG 840
TATTGACGCC GGGCAAGAGC AACTCGGTCG CCGCATACAC TATTCTCAGA ATGACTTGGT 900
TGAGTACTCA CCAGTCACAG AAAAGCATCT TACGGATGGC ATGACAGTAA GAGAATTATG 960
CAGTGCTGCC ATAACCATGA GTGATAACAC TGCGGCCAAC TTACTTCTGA CAACGATCGG 1020
AGGACCGAAG GAGCTAACCG CTTTTTTGCA CAACATGGGG GATCATGTAA CTCGCCTTGA 1080
TCGTTGGGAA CCGGAGCTGA ATGAAGCCAT ACCAAACGAC GAGCGTGACA CCACGATGCC 1140
TGCAGCAATG GCAACAACGT TGCGCAAACT ATTAACTGGC GAACTACTTA CTCTAGCTTC 1200
CCGGCAACAA TTAATAGACT GGATGGAGGC GGATAAAGTT GCAGGACCAC TTCTGCGCTC 1260
GGCCCTTCCG GCTGGCTGGT TTATTGCTGA TAAATCTGGA GCCGGTGAGC GTGGGTCTCG 1320
CGGTATCATT GCAGCACTGG GGCCAGATGG TAAGCCCTCC CGTATCGTAG TTATCTACAC 1380
GACGGGGAGT CAGGCAACTA TGGATGAACG AAATAGACAG ATCGCTGAGA TAGGTGCCTC 1440
ACTGATTAAG CATTGGTAAC TGTCAGACCA AGTTTACTCA TATATACTTT AGATTGATTT 1500
AAAACTTCAT TTTTAATTTA AAAGGATCTA GGTGAAGATC CTTTTTGATA ATCTCATGAC 1560
CAAAATCCCT TAACGTGAGT TTTCGTTCCA CTGAGCGTCA GACCCCGTAG AAAAGATCAA 1620
AGGATCTTCT TGAGATCCTT TTTTTCTGCG CGTAATCTGC TGCTTGCAAA CAAAAAAACC 1680
ACCGCTACCA GCGGTGGTTT GTTTGCCGGA TCAAGAGCTA CCAACTCTTT TTCCGAAGGT 1740
AACTGGCTTC AGCAGAGCGC AGATACCAAA TACTGTCCTT CTAGTGTAGC CGTAGTTAGG 1800
CCACCACTTC AAGAACTCTG TAGCACCGCC TACATACCTC GCTCTGCTAA TCCTGTTACC 1860
AGTGGCTGCT GCCAGTGGCG ATAAGTCGTG TCTTACCGGG TTGGACTCAA GACGATAGTT 1920
ACCGGATAAG GCGCAGCGGT CGGGCTGAAC GGGGGGTTCG TGCACACAGC CCAGCTTGGA 1980
GCGAACGACC TACACCGAAC TGAGATACCT ACAGCGTGAG CTATGAGAAA GCGCCACGCT 2040

TCCCGAAGGG AGAAAGGCGG ACAGGTATCC GGTAAGCGGC AGGGTCGGAA CAGGAGAGCG 2100
CACGAGGGAG CTTCCAGGGG GAAACGCCTG GTATCTTTAT AGTCCTGTCG GGTTTCGCCA 2160
CCTCTGACTT GAGCGTCGAT TTTTGTGATG CTCGTCAGGG GGGCGGAGCC TATGGAAAAA 2220
CGCCAGCAAC GCGGCCTTTT TACGGTTCCT GGCCTTTTGC TGGCCTTTTG CTCACATGTT 2280
CTTTCCTGCG TTATCCCCTG ATTCTGTGGA TAACCGTATT ACCGCCTTTG AGTGAGCTGA 2340
TACCGCTCGC CGCAGCCGAA CGACCGAGCG CAGCGAGTCA GTGAGCGAGG AAGCGGAAGA 2400
GCGCCTGATG CGGTATTTTC TCCTTACGCA TCTGTGCGGT ATTTCACACC GCATATATGG 2460
TGCACTCTCA GTACAATCTG CTCTGATGCC GCATAGTTAA GCCAGTATAC ACTCCGCTAT 2520
CGCTACGTGA CTGGGTCATG GCTGCGCCCC GACACCCGCC AACACCCGCT GACGCGCCCT 2580
GACGGGCTTG TCTGCTCCCG GCATCCGCTT ACAGACAAGC TGTGACCGTC TCCGGGAGCT 2640
GCATGTGTCA GAGGTTTTCA CCGTCATCAC CGAAACGCGC GAGGCAGCTG CGGTAAAGCT 2700
CATCAGCGTG GTCGTGAAGC GATTCACAGA TGTCTGCCTG TTCATCCGCG TCCAGCTCGT 2760
TGAGTTTCTC CAGAAGCGTT AATGTCTGGC TTCTGATAAA GCGGGCCATG TTAAGGGCGG 2820
TTTTTTCCTG TTTGGTCACT GATGCCTCCG TGTAAGGGGG ATTTCTGTTC ATGGGGGTAA 2880
TGATACCGAT GAAACGAGAG AGGATGCTCA CGATACGGGT TACTGATGAT GAACATGCCC 2940
GGTTACTGGA ACGTTGTGAG GGTAAACAAC TGGCGGTATG GATGCGGCGG GACCAGAGAA 3000
AAATCACTCA GGGTCAATGC CAGCGCTTCG TTAATACAGA TGTAGGTGTT CCACAGGGTA 3060
GCCAGCAGCA TCCTGCGATG CAGATCCGGA ACATAATGGT GCAGGGCGCT GACTTCCGCG 3120
TTTCCAGACT TTACGAAACA CGGAAACCGA AGACCATTCA TGTTGTTGCT CAGGTCGCAG 3180
ACGTTTTGCA GCAGCAGTCG CTTCACGTTC GCTCGCGTAT CGGTGATTCA TTCTGCTAAC 3240
CAGTAAGGCA ACCCCGCCAG CCTAGCCGGG TCCTCAACGA CAGGAGCACG ATCATGCGCA 3300
CCCGTGGGGC CGCCATGCCG GCGATAATGG CCTGCTTCTC GCCGAAACGT TTGGTGGCGG 3360
GACCAGTGAC GAAGGCTTGA GCGAGGGCGT GCAAGATTCC GAATACCGCA AGCGACAGGC 3420
CGATCATCGT CGCGCTCCAG CGAAAGCGGT CCTCGCCGAA AATGACCCAG AGCGCTGCCG 3480
GCACCTGTCC TACGAGTTGC ATGATAAAGA AGACAGTCAT AAGTGCGGCG ACGATAGTCA 3540
TGCCCCGCGC CCACCGGAAG GAGCTGACTG GGTTGAAGGC TCTCAAGGGC ATCGGTCGAG 3600
ATCCCGGTGC CTAATGAGTG AGCTAACTTA CATTAATTGC GTTGCGCTCA CTGCCCGCTT 3660
TCCAGTCGGG AAACCTGTCG TGCCAGCTGC ATTAATGAAT CGGCCAACGC GCGGGGAGAG 3720
GCGGTTTGCG TATTGGGCGC CAGGGTGGTT TTTCTTTTCA CCAGTGAGAC GGGCAACAGC 3780
TGATTGCCCT TCACCGCCTG GCCCTGAGAG AGTTGCAGCA AGCGGTCCAC GCTGGTTTGC 3840
CCCAGCAGGC GAAAATCCTG TTTGATGGTG GTTAACGGCG GGATATAACA TGAGCTGTCT 3900
TCGGTATCGT CGTATCCCAC TACCGAGATA TCCGCACCAA CGCGCAGCCC GGACTCGGTA 3960
ATGGCGCGCA TTGCGCCCAG CGCCATCTGA TCGTTGGCAA CCAGCATCGC AGTGGGAACG 4020
ATGCCCTCAT TCAGCATTTG CATGGTTTGT TGAAAACCGG ACATGGCACT CCAGTCGCCT 4080
TCCCGTTCCG CTATCGGCTG AATTTGATTG CGAGTGAGAT ATTTATGCCA GCCAGCCAGA 4140
CGCAGACGCG CCGAGACAGA ACTTAATGGG CCCGCTAACA GCGCGATTTG CTGGTGACCC 4200

AATGCGACCA GATGCTCCAC GCCCAGTCGC GTACCGTCTT CATGGGAGAA AATAATACTG 4260

TTGATGGGTG TCTGGTCAGA GACATCAAGA AATAACGCCG GAACATTAGT GCAGGCAGCT 4320

TCCACAGCAA TGGCATCCTG GTCATCCAGC GGATAGTTAA TGATCAGCCC ACTGACGCGT 4380

TGCGCGAGAA GATTGTGCAC CGCCGCTTTA CAGGCTTCGA CGCCGCTTCG TTCTACCATC 4440

GACACCACCA CGCTGGCACC CAGTTGATCG GCGCGAGATT TAATCGCCGC GACAATTTGC 4500

GACGGCGCGT GCAGGGCCAG ACTGGAGGTG GCAACGCCAA TCAGCAACGA CTGTTTGCCC 4560

GCCAGTTGTT GTGCCACGCG GTTGGGAATG TAATTCAGCT CCGCCATCGC CGCTTCCACT 4620

TTTTCCCGCG TTTTCGCAGA AACGTGGCTG GCCTGGTTCA CCACGCGGGA AACGGTCTGA 4680

TAAGAGACAC CGGCATACTC TGCGACATCG TATAACGTTA CTGGTTTCAC ATTCACCACC 4740

CTGAATTGAC TCTCTTCCGG GCGCTATCAT GCCATACCGC GAAAGGTTTT GCGCCATTCG 4800

ATGGTGTCCG GGATCTCGAC GCTCTCCCTT ATGCGACTCC TGCATTAGGA AGCAGCCCAG 4860

TAGTAGGTTG AGGCCGTTGA GCACCGCCGC CGCAAGGAAT GGTGCATGCA AGGAGATGGC 4920

GCCCAACAGT CCCCCGGCCA CGGGGCCTGC CACCATACCC ACGCCGAAAC AAGCGCTCAT 4980

GAGCCCGAAG TGGCGAGCCC GATCTTCCCC ATCGGTGATG TCGGCGATAT AGGCGCCAGC 5040

AACCGCACCT GTGGCGCCGG TGATGCCGGC CACGATGCGT CCGGCGTAGA GGATCGAGAT 5100

T7 promoter **Lac operator**

CTCGATCCCG CGAAATTAAT ACGACTCACT ATAGGGGAAT TGTGAGCGGA TAACAATTCC 5160

C2 domain

CCTCTAGAAA TAATTTTGTT TAACTTTAAG AAGGAGATAT ACATATGCAC GGATGTTCTG 5220

AGCCCCTGGG CCTGAAGAAT AACACAATTC CTGACAGCCA GATGTCAGCC TCCAGCAGCT 5280

ACAAGACATG GAACCTGCGT GCTTTTGGCT GGTACCCCCA CTTGGGAAGG CTGGATAATC 5340

AGGGCAAGAT CAATGCCTGG ACGGCTCAGA GCAACAGTGC CAAGGAATGG CTGCAGGTTG 5400

ACCTGGGCAC TCAGAGGCAA GTGACAGGAA TCATCACCCA GGGGGCCCGT GACTTTGGCC 5460

ACATCCAGTA TGTGGCGTCC TACAAGGTAG CCCACAGTGA TGATGGTGTG CAGTGGACTG 5520

TATATGAGGA GCAAGGAAGC AGCAAGGTCT TCCAGGGCAA CTTGGACAAC AACTCCCACA 5580

AGAAGAACAT CTTCGAGAAA CCCTTCATGG CTCGCTACGT GCGTGTCCTT CCAGTGTCCT 5640

linker

GGCATAACCG CATCACCCTG CGCCTGGAGC TGCTGGGCTG TAAGCTTGGC ACTGGAGGCT 5700

CTGGAGGCAC TGGAGGCTCT GGAGGCACCG GTAGCACTAG CGGCGGAAGC GGCGGGACAG 5760

SNAP-tag

GTACGCGTGA CAAAGACTGC GAAATGAAGC GCACCACCCT GGATAGCCCT CTGGGCAAGC 5820

TGGAACTGTC TGGGTGCGAA CAGGGCCTGC ACCGTATCAT CTTCCTGGGC AAAGGAACAT 5880

CTGCCGCCGA CGCCGTGGAA GTGCCTGCCC CAGCCGCCGT GCTGGGCGGA CCAGAGCCAC 5940

TGATGCAGGC CACCGCCTGG CTCAACGCCT ACTTTCACCA GCCTGAGGCC ATCGAGGAGT 6000

TCCCTGTGCC AGCCCTGCAC CACCCAGTGT TCCAGCAGGA GAGCTTTACC CGCCAGGTGC 6060

TGTGGAAACT GCTGAAAGTG GTGAAGTTCG GAGAGGTCAT CAGCTACAGC CACCTGGCCG 6120

CCCTGGCCGG CAATCCCGCC GCCACCGCCG CCGTGAAAAC CGCCCTGAGC GGAAATCCCG 6180

TGCCCATTCT GATCCCCTGC CACCGGGTGG TGCAGGGCGA CCTGGACGTG GGGGGCTACG 6240

AGGGCGGGCT CGCCGTGAAA GAGTGGCTGC TGGCCCACGA GGGCCACAGA CTGGGCAAGC 6300

His-tag

CTGGGCTGGG TCTCGAGCAC CACCACCACC ACCACTGAGA TCCGGCTGCT AACAAAGCCC 6360

T7 term

GAAAGGAAGC TGAGTTGGCT GCTGCCACCG CTGAGCAATA ACTAGCATAA CCCCTTGGGG 6420

CCTCTAAACG GGTCTTGAGG GGTTTTTTGC TGAAAGGAGG AACTATATCC GGAT 6474

Sequence Name: pET21a(+)-C2m2-SNAP

Length: 6474

TGGCGAATGG GACGCGCCCT GTAGCGGCGC ATTAAGCGCG GCGGGTGTGG TGGTTACGCG 60

CAGCGTGACC GCTACACTTG CCAGCGCCCT AGCGCCCGCT CCTTTCGCTT TCTTCCCTTC 120

CTTTCTCGCC ACGTTCGCCG GCTTTCCCCG TCAAGCTCTA AATCGGGGGC TCCCTTTAGG 180

GTTCCGATTT AGTGCTTTAC GGCACCTCGA CCCCAAAAAA CTTGATTAGG GTGATGGTTC 240

ACGTAGTGGG CCATCGCCCT GATAGACGGT TTTTCGCCCT TTGACGTTGG AGTCCACGTT 300

CTTTAATAGT GGACTCTTGT TCCAAACTGG AACAACACTC AACCCTATCT CGGTCTATTC 360

TTTTGATTTA TAAGGGATTT TGCCGATTTC GGCCTATTGG TTAAAAAATG AGCTGATTTA 420

ACAAAAATTT AACGCGAATT TTAACAAAAT ATTAACGTTT ACAATTTCAG GTGGCACTTT 480

TCGGGGAAAT GTGCGCGGAA CCCCTATTTG TTTATTTTTC TAAATACATT CAAATATGTA 540

TCCGCTCATG AGACAATAAC CCTGATAAAT GCTTCAATAA TATTGAAAAA GGAAGAGTAT 600

GAGTATTCAA CATTTCCGTG TCGCCCTTAT TCCCTTTTTT GCGGCATTTT GCCTTCCTGT 660

TTTTGCTCAC CCAGAAACGC TGGTGAAAGT AAAAGATGCT GAAGATCAGT TGGGTGCACG 720

AGTGGGTTAC ATCGAACTGG ATCTCAACAG CGGTAAGATC CTTGAGAGTT TTCGCCCCGA 780

AGAACGTTTT CCAATGATGA GCACTTTTAA AGTTCTGCTA TGTGGCGCGG TATTATCCCG 840

TATTGACGCC GGGCAAGAGC AACTCGGTCG CCGCATACAC TATTCTCAGA ATGACTTGGT 900

TGAGTACTCA CCAGTCACAG AAAAGCATCT TACGGATGGC ATGACAGTAA GAGAATTATG 960

CAGTGCTGCC ATAACCATGA GTGATAACAC TGCGGCCAAC TTACTTCTGA CAACGATCGG 1020

AGGACCGAAG GAGCTAACCG CTTTTTTGCA CAACATGGGG GATCATGTAA CTCGCCTTGA 1080

TCGTTGGGAA CCGGAGCTGA ATGAAGCCAT ACCAAACGAC GAGCGTGACA CCACGATGCC 1140

TGCAGCAATG GCAACAACGT TGCGCAAACT ATTAACTGGC GAACTACTTA CTCTAGCTTC 1200

CCGGCAACAA TTAATAGACT GGATGGAGGC GGATAAAGTT GCAGGACCAC TTCTGCGCTC 1260

GGCCCTTCCG GCTGGCTGGT TTATTGCTGA TAAATCTGGA GCCGGTGAGC GTGGGTCTCG 1320

CGGTATCATT GCAGCACTGG GGCCAGATGG TAAGCCCTCC CGTATCGTAG TTATCTACAC 1380

GACGGGGAGT CAGGCAACTA TGGATGAACG AAATAGACAG ATCGCTGAGA TAGGTGCCTC 1440

ACTGATTAAG CATTGGTAAC TGTCAGACCA AGTTTACTCA TATATACTTT AGATTGATTT 1500

AAAACTTCAT TTTTAATTTA AAAGGATCTA GGTGAAGATC CTTTTTGATA ATCTCATGAC 1560

CAAAATCCCT TAACGTGAGT TTTCGTTCCA CTGAGCGTCA GACCCCGTAG AAAAGATCAA 1620

AGGATCTTCT TGAGATCCTT TTTTTCTGCG CGTAATCTGC TGCTTGCAAA CAAAAAAACC 1680

ACCGCTACCA GCGGTGGTTT GTTTGCCGGA TCAAGAGCTA CCAACTCTTT TTCCGAAGGT 1740

AACTGGCTTC AGCAGAGCGC AGATACCAAA TACTGTCCTT CTAGTGTAGC CGTAGTTAGG 1800

CCACCACTTC AAGAACTCTG TAGCACCGCC TACATACCTC GCTCTGCTAA TCCTGTTACC 1860

AGTGGCTGCT GCCAGTGGCG ATAAGTCGTG TCTTACCGGG TTGGACTCAA GACGATAGTT 1920

ACCGGATAAG GCGCAGCGGT CGGGCTGAAC GGGGGGTTCG TGCACACAGC CCAGCTTGGA 1980

GCGAACGACC TACACCGAAC TGAGATACCT ACAGCGTGAG CTATGAGAAA GCGCCACGCT 2040

TCCCGAAGGG AGAAAGGCGG ACAGGTATCC GGTAAGCGGC AGGGTCGGAA CAGGAGAGCG 2100
CACGAGGGAG CTTCCAGGGG GAAACGCCTG GTATCTTTAT AGTCCTGTCG GGTTTCGCCA 2160
CCTCTGACTT GAGCGTCGAT TTTTGTGATG CTCGTCAGGG GGGCGGAGCC TATGGAAAAA 2220
CGCCAGCAAC GCGGCCTTTT TACGGTTCCT GGCCTTTTGC TGGCCTTTTG CTCACATGTT 2280
CTTTCCTGCG TTATCCCCTG ATTCTGTGGA TAACCGTATT ACCGCCTTTG AGTGAGCTGA 2340
TACCGCTCGC CGCAGCCGAA CGACCGAGCG CAGCGAGTCA GTGAGCGAGG AAGCGGAAGA 2400
GCGCCTGATG CGGTATTTTC TCCTTACGCA TCTGTGCGGT ATTTCACACC GCATATATGG 2460
TGCACTCTCA GTACAATCTG CTCTGATGCC GCATAGTTAA GCCAGTATAC ACTCCGCTAT 2520
CGCTACGTGA CTGGGTCATG GCTGCGCCCC GACACCCGCC AACACCCGCT GACGCGCCCT 2580
GACGGGCTTG TCTGCTCCCG GCATCCGCTT ACAGACAAGC TGTGACCGTC TCCGGGAGCT 2640
GCATGTGTCA GAGGTTTTCA CCGTCATCAC CGAAACGCGC GAGGCAGCTG CGGTAAAGCT 2700
CATCAGCGTG GTCGTGAAGC GATTCACAGA TGTCTGCCTG TTCATCCGCG TCCAGCTCGT 2760
TGAGTTTCTC CAGAAGCGTT AATGTCTGGC TTCTGATAAA GCGGGCCATG TTAAGGGCGG 2820
TTTTTTCCTG TTTGGTCACT GATGCCTCCG TGTAAGGGGG ATTTCTGTTC ATGGGGGTAA 2880
TGATACCGAT GAAACGAGAG AGGATGCTCA CGATACGGGT TACTGATGAT GAACATGCCC 2940
GGTTACTGGA ACGTTGTGAG GGTAAACAAC TGGCGGTATG GATGCGGCGG GACCAGAGAA 3000
AAATCACTCA GGGTCAATGC CAGCGCTTCG TTAATACAGA TGTAGGTGTT CCACAGGGTA 3060
GCCAGCAGCA TCCTGCGATG CAGATCCGGA ACATAATGGT GCAGGGCGCT GACTTCCGCG 3120
TTTCCAGACT TTACGAAACA CGGAAACCGA AGACCATTCA TGTTGTTGCT CAGGTCGCAG 3180
ACGTTTTGCA GCAGCAGTCG CTTCACGTTC GCTCGCGTAT CGGTGATTCA TTCTGCTAAC 3240
CAGTAAGGCA ACCCCGCCAG CCTAGCCGGG TCCTCAACGA CAGGAGCACG ATCATGCGCA 3300
CCCGTGGGGC CGCCATGCCG GCGATAATGG CCTGCTTCTC GCCGAAACGT TTGGTGGCGG 3360
GACCAGTGAC GAAGGCTTGA GCGAGGGCGT GCAAGATTCC GAATACCGCA AGCGACAGGC 3420
CGATCATCGT CGCGCTCCAG CGAAAGCGGT CCTCGCCGAA AATGACCCAG AGCGCTGCCG 3480
GCACCTGTCC TACGAGTTGC ATGATAAAGA AGACAGTCAT AAGTGCGGCG ACGATAGTCA 3540
TGCCCCGCGC CCACCGGAAG GAGCTGACTG GGTTGAAGGC TCTCAAGGGC ATCGGTCGAG 3600
ATCCCGGTGC CTAATGAGTG AGCTAACTTA CATTAATTGC GTTGCGCTCA CTGCCCGCTT 3660
TCCAGTCGGG AAACCTGTCG TGCCAGCTGC ATTAATGAAT CGGCCAACGC GCGGGGAGAG 3720
GCGGTTTGCG TATTGGGCGC CAGGGTGGTT TTTCTTTTCA CCAGTGAGAC GGGCAACAGC 3780
TGATTGCCCT TCACCGCCTG GCCCTGAGAG AGTTGCAGCA AGCGGTCCAC GCTGGTTTGC 3840
CCCAGCAGGC GAAAATCCTG TTTGATGGTG GTTAACGGCG GGATATAACA TGAGCTGTCT 3900
TCGGTATCGT CGTATCCCAC TACCGAGATA TCCGCACCAA CGCGCAGCCC GGACTCGGTA 3960
ATGGCGCGCA TTGCGCCCAG CGCCATCTGA TCGTTGGCAA CCAGCATCGC AGTGGGAACG 4020
ATGCCCTCAT TCAGCATTTG CATGGTTTGT TGAAAACCGG ACATGGCACT CCAGTCGCCT 4080
TCCCGTTCCG CTATCGGCTG AATTTGATTG CGAGTGAGAT ATTTATGCCA GCCAGCCAGA 4140
CGCAGACGCG CCGAGACAGA ACTTAATGGG CCCGCTAACA GCGCGATTTG CTGGTGACCC 4200

AATGCGACCA GATGCTCCAC GCCCAGTCGC GTACCGTCTT CATGGGAGAA AATAATACTG 4260

TTGATGGGTG TCTGGTCAGA GACATCAAGA AATAACGCCG GAACATTAGT GCAGGCAGCT 4320

TCCACAGCAA TGGCATCCTG GTCATCCAGC GGATAGTTAA TGATCAGCCC ACTGACGCGT 4380

TGCGCGAGAA GATTGTGCAC CGCCGCTTTA CAGGCTTCGA CGCCGCTTCG TTCTACCATC 4440

GACACCACCA CGCTGGCACC CAGTTGATCG GCGCGAGATT TAATCGCCGC GACAATTTGC 4500

GACGGCGCGT GCAGGGCCAG ACTGGAGGTG GCAACGCCAA TCAGCAACGA CTGTTTGCCC 4560

GCCAGTTGTT GTGCCACGCG GTTGGGAATG TAATTCAGCT CCGCCATCGC CGCTTCCACT 4620

TTTTCCCGCG TTTTCGCAGA AACGTGGCTG GCCTGGTTCA CCACGCGGGA AACGGTCTGA 4680

TAAGAGACAC CGGCATACTC TGCGACATCG TATAACGTTA CTGGTTTCAC ATTCACCACC 4740

CTGAATTGAC TCTCTTCCGG GCGCTATCAT GCCATACCGC GAAAGGTTTT GCGCCATTCG 4800

ATGGTGTCCG GGATCTCGAC GCTCTCCCTT ATGCGACTCC TGCATTAGGA AGCAGCCCAG 4860

TAGTAGGTTG AGGCCGTTGA GCACCGCCGC CGCAAGGAAT GGTGCATGCA AGGAGATGGC 4920

GCCCAACAGT CCCCCGGCCA CGGGGCCTGC CACCATACCC ACGCCGAAAC AAGCGCTCAT 4980

GAGCCCGAAG TGGCGAGCCC GATCTTCCCC ATCGGTGATG TCGGCGATAT AGGCGCCAGC 5040

AACCGCACCT GTGGCGCCGG TGATGCCGGC CACGATGCGT CCGGCGTAGA GGATCGAGAT 5100

T7 promoter **Lac operator**

CTCGATCCCG CGAAATTAAT ACGACTCACT ATAGGGGAAT TGTGAGCGGA TAACAATTCC 5160

C2 domain

CCTCTAGAAA TAATTTTGTT TAACTTTAAG AAGGAGATAT ACATATGCAC GGATGTTCTG 5220

AGCCCCTGGG CCTGAAGAAT AACACAATTC CTGACAGCCA GATGTCAGCC TCCAGCAGCT 5280

ACAATACATG GAACCTGCGT GCTTTTGGCT GGTACCCCCA CTTGGGAAGG CTGGATAATC 5340

AGGGCAATAT CAATGCCTGG ACGGCTCAGA GCAACAGTGC CAAGGAATGG CTGCAGGTTG 5400

ACCTGGGCAC TCAGAGGCAA GTGACAGGAA TCATCACCCA GGGGGCCCGT GACTTTGGCC 5460

ACATCCAGTA TGTGGCGTCC TACAAGGTAG CCCACAGTGA TGATGGTGTG CAGTGGACTG 5520

TATATGAGGA GCAAGGAAGC AGCAAGGTCT TCCAGGGCAA CTTGGACAAC AACTCCCACA 5580

AGAAGAACAT CTTCGAGAAA CCCTTCATGG CTCGCTACGT GCGTGTCCTT CCAGTGTCCT 5640

linker

GGCATAACCG CATCACCCTG CGCCTGGAGC TGCTGGGCTG TAAGCTTGGC ACTGGAGGCT 5700

CTGGAGGCAC TGGAGGCTCT GGAGGCACCG GTAGCACTAG CGGCGGAAGC GGCGGGACAG 5760

SNAP-tag

GTACGCGTGA CAAAGACTGC GAAATGAAGC GCACCACCCT GGATAGCCCT CTGGGCAAGC 5820

TGGAACTGTC TGGGTGCGAA CAGGGCCTGC ACCGTATCAT CTTCCTGGGC AAAGGAACAT 5880

CTGCCGCCGA CGCCGTGGAA GTGCCTGCCC CAGCCGCCGT GCTGGGCGGA CCAGAGCCAC 5940

TGATGCAGGC CACCGCCTGG CTCAACGCCT ACTTTCACCA GCCTGAGGCC ATCGAGGAGT 6000

TCCCTGTGCC AGCCCTGCAC CACCCAGTGT TCCAGCAGGA GAGCTTTACC CGCCAGGTGC 6060

TGTGGAAACT GCTGAAAGTG GTGAAGTTCG GAGAGGTCAT CAGCTACAGC CACCTGGCCG 6120

CCCTGGCCGG CAATCCCGCC GCCACCGCCG CCGTGAAAAC CGCCCTGAGC GGAAATCCCG 6180

TGCCCATTCT GATCCCCTGC CACCGGGTGG TGCAGGGCGA CCTGGACGTG GGGGGCTACG 6240

AGGGCGGGCT CGCCGTGAAA GAGTGGCTGC TGGCCCACGA GGGCCACAGA CTGGGCAAGC 6300

His-tag

CTGGGCTGGG TCTCGAGCAC CACCACCACC ACCACTGAGA TCCGGCTGCT AACAAAGCCC 6360

T7 term

GAAAGGAAGC TGAGTTGGCT GCTGCCACCG CTGAGCAATA ACTAGCATAA CCCCTTGGGG 6420

CCTCTAAACG GGTCTTGAGG GGTTTTTTGC TGAAAGGAGG AACTATATCC GGAT 6474

Sequence Name: pAAV-C2-mKate

Length: 5852

TTGGCCACTC CCTCTCTGCG CGCTCGCTCG CTCACTGAGG CCGGGCGACC AAAGGTCGCC 60

CGACGCCCGG GCTTTGCCCG GGCGGCCTCA GTGAGCGAGC GAGCGCGCAG AGAGGGAGTG 120

GFAP promoter

GCCAACTCCA TCACTAGGGG TTCCTACTAG TAACATATCC TGGTGTGGAG TAGGGGACGC 180

TGCTCTGACA GAGGCTCGGG GGCCTGAGCT GGCTCTGTGA GCTGGGGAGG AGGCAGACAG 240

CCAGGCCTTG TCTGCAAGCA GACCTGGCAG CATTGGGCTG GCCGCCCCCC AGGGCCTCCT 300

CTTCATGCCC AGTGAATGAC TCACCTTGGC ACAGACACAA TGTTCGGGGT GGGCACAGTG 360

CCTGCTTCCC GCCGCACCCC AGCCCCCCTC AAATGCCTTC CGAGAAGCCC ATTGAGCAGG 420

GGGCTTGCAT TGCACCCCAG CCTGACAGCC TGGCATCTTG GGATAAAAGC AGCACAGCCC 480

CCTAGGGGCT GCCCTTGCTG TGTGGCGCCA CCGGCGGTGG AGAACAAGGC TCTATTCAGC 540

CTGTGCCCAG GAAAGGGGAT CAGGGGATGC CCAGGCATGG ACAGTGGGTG GCAGGGGGGG 600

AGAGGAGGGC TGTCTGCTTC CCAGAAGTCC AAGGACACAA ATGGGTGAGG GGAGAGCTCT 660

CCCCATAGCT GGGCTGCGGC CCAACCCCAC CCCCTCAGGC TATGCCAGGG GGTGTTGCCA 720

GGGGCACCCG GGCATCGCCA GTCTAGCCCA CTCCTTCATA AAGCCCTCGT ATCCCAGGAG 780

CGAGCAGAGC CAGAGCAGGT TGGAGAGGAG ACGCATCACC TCTGCTGCTT GCCTAAAACA 840

GGTAAGTCCC ATTAATCTCC CTATCAGTGA TAGAGAAGGT CTGAAGAGTT TACTCCCTAT 900

CAGTGATAGA GATTAATTCC TCTACTAACC TTGTTCATCT TTTCTTTTTT TTTCTACAGG 960

Kozak **Signal peptide**

TCCTGGGTGA TTAACAGCTT AAGGCCGCCA CCATGCAGGT CTCCCGTGTG CTGGCCGCGC 1020

C2 domain

TGTGCGGCAT GCTACTCTGC GCCTCTGGCC TCTTCGCCGC GTCTGGTGAC CATATGCACG 1080

GATGTTCTGA GCCCCTGGGC CTGAAGAATA ACACAATTCC TGACAGCCAG ATGTCAGCCT 1140

CCAGCAGCTA CAAGACATGG AACCTGCGTG CTTTTGGCTG GTACCCCCAC TTGGGAAGGC 1200

TGGATAATCA GGGCAAGATC AATGCCTGGA CGGCTCAGAG CAACAGTGCC AAGGAATGGC 1260

TGCAGGTTGA CCTGGGCACT CAGAGGCAAG TGACAGGAAT CATCACCCAG GGGGCCCGTG 1320

ACTTTGGCCA CATCCAGTAT GTGGCGTCCT ACAAGGTAGC CCACAGTGAT GATGGTGTGC 1380

AGTGGACTGT ATATGAGGAG CAAGGAAGCA GCAAGGTCTT CCAGGGCAAC TTGGACAACA 1440

ACTCCCACAA GAAGAACATC TTCGAGAAAC CCTTCATGGC TCGCTACGTG CGTGTCCTTC 1500

linker

CAGTGTCCTG GCATAACCGC ATCACCCTGC GCCTGGAGCT GCTGGGCTGT AAGCTTGGCA 1560

CTGGAGGCTC TGGAGGCACT GGAGGCTCTG GAGGCACCGG TAGCACTAGC GGCGGAAGCG 1620

mKate

GCGGGACAGG TACGCGTGTG AGCGAGCTGA TTAAGGAGAA CATGCACATG AAGCTGTACA 1680

TGGAGGGCAC CGTGAACAAC CACCACTTCA AGTGCACATC CGAGGGCGAA GGCAAGCCCT 1740

ACGAGGGCAC CCAGACCATG AGAATCAAGG TCGTCGAGGG CGGCCCTCTC CCCTTCGCCT 1800

TCGACATCCT GGCTACCAGC TTCATGTACG GCAGCAAAAC CTTCATCAAC CACCCTCAGG 1860

GCATCCCCGA CTTCTTTAAG CAGTCCTTCC CTGAGGGCTT CACATGGGAG AGAGTCACCA 1920

CATACGAAGA CGGGGGCGTG CTGACCGCTA CCCAGGACAC CAGCCTCCAG GACGGCTGCC 1980

TCATCTACAA CGTCAAGATT AGAGGGGTGA ACTTCCCAGC CAACGGCCCT GTGATGCAGA 2040

AGAAAACACT CGGCTGGGAG GCCTCCACCG AGACGCTGTA CCCCGCTGAC GGCGGCCTGG 2100

AAGGCGCATG TGACATGGCC CTGAAGCTCG TGGGCGGGGG CCACCTGATC TGCAACTTGG 2160

AGACCACATA CAGATCCAAG AAACCCGCTA AGAACCTCAA GATGCCCGGC GTCTACAACG 2220

TGGACAGGAG ACTGGAAAGA ATCAAGGAGG CCGACAATGA GACCTACGTC GAGCAGCACG 2280

AGGTGGCTGT GGCCAGATAC TCTACTGGTG GCGCTGGTGA TGGAGGTAAA TGAGGATCCT 2340

WPRE

AATCAACCTC TGGATTACAA AATTTGTGAA AGATTGACTG GTATTCTTAA CTATGTTGCT 2400

CCTTTTACGC TATGTGGATA CGCTGCTTTA ATGCCTTTGT ATCATGCTAT TGCTTCCCGT 2460

ATGGCTTTCA TTTTCTCCTC CTTGTATAAA TCCTGGTTGC TGTCTCTTTA TGAGGAGTTG 2520

TGGCCCGTTG TCAGGCAACG TGGCGTGGTG TGCACTGTGT TTGCTGACGC AACCCCCACT 2580

GGTTGGGGCA TTGCCACCAC CTGTCAGCTC CTTTCCGGGA CTTTCGCTTT CCCCCTCCCT 2640

ATTGCCACGG CGGAACTCAT CGCCGCCTGC CTTGCCCGCT GCTGGACAGG GGCTCGGCTG 2700

TTGGGCACTG ACAATTCCGT GGTGTTGTCG GGGAAATCAT CGTCCTTTCC TTGGCTGCTC 2760

GCCTGTGTTG CCACCTGGAT TCTGCGCGGG ACGTCCTTCT GCTACGTCCC TTCGGCCCTC 2820

AATCCAGCGG ACCTTCCTTC CCGCGGCCTG CTGCCGGCTC TGCGGCCTCT TCCGCGTCTT 2880

CGCCTTCGCC CTCAGACGAG TCGGATCTCC CTTTGGGCCG CCTCCCCGCC TGAATTCTGC 2940

ATGTTTAAAC ATACATACTT CTTTACATTC CAGATATCTG CATCAAACAC CATTGTCACA 3000

CTCCATCGCG ATCACGCGAG CCGAACGAAC AAACCCAACA ACATGAAACT ACCTAGCGCT 3060

TCTATTCAGC ATGATGTCTT TCGTATACAC AAATTCGGTT CTACAGGGTA ACCTAGGCTG 3120

CATGTTTAAA CATACATACT TCTTTACATT CCAGATATCT GCATCAAACA CCATTGTCAC 3180

ACTCCATCGC GATCACGCGA GCCGAACGAA CAAACCCAAC AACATGAAAC TACCTAGCGC 3240

TTCTATTCAG CATGATGTCT TTCGTATACA CAAATTCGGT TCTACAGGGT AACCTAGGCT 3300

GCATGTTTAA ACATACATAC TTCTTTACAT TCCAGATATC TGCATCAAAC ACCATTGTCA 3360

CACTCCATCG CGATCACGCG AGCCGAACGA ACAAACCCAA CAACATGAAA CTACCTAGCG 3420

CTTCTATTCA GCATGATGTC TTTCGTATAC ACAAATTCGG TTCTACAGGG TAACCTAGGC 3480

SV40 poly(A)

TGCAGGTACC TTCGAGCAGA CATGATAAGA TACATTGATG AGTTTGGACA AACCACAACT 3540

AGAATGCAGT GAAAAAAATG CTTTATTTGT GAAATTTGTG ATGCTATTGC TTTATTTGTA 3600

ACCATTATAA GCTGCAATAA ACAAGTTAAC AACAACAATT GCATTCATTT TATGTTTCAG 3660

GTTCAGGGGG AGATGTGGGA GGTTTTTTAA AGCAAGTAAA ACCTCTACAA ATGTGGTAAA 3720

ATCAAGCTTA GGAACCCCTA GTGATGGAGT TGGCCACTCC CTCTCTGCGC GCTCGCTCGC 3780

TCACTGAGGC CGGGCGACCA AAGGTCGCCC GACGCCCGGG CTTTGCCCGG GCGGCCTCAG 3840

TGAGCGAGCG AGCGCGCAGA GAGGGAGTGG CCAAGCTAGC GGGCGATTAA GGAAAGGGCT 3900

AGATCATTCT TGAAGACGAA AGGGCCTCGT GATACGCCTA TTTTTATAGG TTAATGTCAT 3960

GATAATAATG GTTTCTTAGA CGTCAGGTGG CACTTTTCGG GGAAATGTGC GCGGAACCCC 4020

TATTTGTTTA TTTTTCTAAA TACATTCAAA TATGTATCCG CTCATGAGAC AATAACCCTG 4080

ATAAATGCTT CAATAATATT GAAAAAGGAA GAGTATGAGT ATTCAACATT TCCGTGTCGC 4140

CCTTATTCCC TTTTTTGCGG CATTTTGCCT TCCTGTTTTT GCTCACCCAG AAACGCTGGT 4200

GAAAGTAAAA GATGCTGAAG ATCAGTTGGG TGCACGAGTG GGTTACATCG AACTGGATCT 4260

CAACAGCGGT AAGATCCTTG AGAGTTTTCG CCCCGAAGAA CGTTTTCCAA TGATGAGCAC 4320

TTTTAAAGTT CTGCTATGTG GCGCGGTATT ATCCCGTGTT GACGCCGGGC AAGAGCAACT 4380

CGGTCGCCGC ATACACTATT CTCAGAATGA CTTGGTTGAG TACTCACCAG TCACAGAAAA 4440

GCATCTTACG GATGGCATGA CAGTAAGAGA ATTATGCAGT GCTGCCATAA CCATGAGTGA 4500

TAACACTGCG GCCAACTTAC TTCTGACAAC GATCGGAGGA CCGAAGGAGC TAACCGCTTT 4560

TTTGCACAAC ATGGGGGATC ATGTAACTCG CCTTGATCGT TGGGAACCGG AGCTGAATGA 4620

AGCCATACCA AACGACGAGC GTGACACCAC GATGCCTGTA GCAATGGCAA CAACGTTGCG 4680

CAAACTATTA ACTGGCGAAC TACTTACTCT AGCTTCCCGG CAACAATTAA TAGACTGGAT 4740

GGAGGCGGAT AAAGTTGCAG GACCACTTCT GCGCTCGGCC CTTCCGGCTG GCTGGTTTAT 4800

TGCTGATAAA TCTGGAGCCG GTGAGCGTGG GTCTCGCGGT ATCATTGCAG CACTGGGGCC 4860

AGATGGTAAG CCCTCCCGTA TCGTAGTTAT CTACACGACG GGGAGTCAGG CAACTATGGA 4920

TGAACGAAAT AGACAGATCG CTGAGATAGG TGCCTCACTG ATTAAGCATT GGTAACTGTC 4980

AGACCAAGTT TACTCATATA TACTTTAGAT TGATTTAAAA CTTCATTTTT AATTTAAAAG 5040

GATCTAGGTG AAGATCCTTT TTGATAATCT CATGACCAAA ATCCCTTAAC GTGAGTTTTC 5100

GTTCCACTGA GCGTCAGACC CCGTAGAAAA GATCAAAGGA TCTTCTTGAG ATCCTTTTTT 5160

TCTGCGCGTA ATCTGCTGCT TGCAAACAAA AAAACCACCG CTACCAGCGG TGGTTTGTTT 5220

GCCGGATCAA GAGCTACCAA CTCTTTTTCC GAAGGTAACT GGCTTCAGCA GAGCGCAGAT 5280

ACCAAATACT GTTCTTCTAG TGTAGCCGTA GTTAGGCCAC CACTTCAAGA ACTCTGTAGC 5340

ACCGCCTACA TACCTCGCTC TGCTAATCCT GTTACCAGTG GCTGCTGCCA GTGGCGATAA 5400

GTCGTGTCTT ACCGGGTTGG ACTCAAGACG ATAGTTACCG GATAAGGCGC AGCGGTCGGG 5460

CTGAACGGGG GGTTCGTGCA CACAGCCCAG CTTGGAGCGA ACGACCTACA CCGAACTGAG 5520

ATACCTACAG CGTGAGCTAT GAGAAAGCGC CACGCTTCCC GAAGGGAGAA AGGCGGACAG 5580

GTATCCGGTA AGCGGCAGGG TCGGAACAGG AGAGCGCACG AGGGAGCTTC CAGGGGGAAA 5640

CGCCTGGTAT CTTTATAGTC CTGTCGGGTT TCGCCACCTC TGACTTGAGC GTCGATTTTT 5700

GTGATGCTCG TCAGGGGGGC GGAGCCTATG GAAAAACGCC AGCAACGCGG CCTTTTTACG 5760

GTTCCTGGCC TTTTGCTGGC CTTTTGCTCA CATGTAATAA ACACACACAC ACCAACAACC 5820

GTGGTTGGTT GTTGTGTTGG TTTATTCTCG AG 5852

Sequence Name: pAAV-C2-SNAP

Length: 5699

TTGGCCACTC CCTCTCTGCG CGCTCGCTCG CTCACTGAGG CCGGGCGACC AAAGGTCGCC 60

CGACGCCCGG GCTTTGCCCG GGCGGCCTCA GTGAGCGAGC GAGCGCGCAG AGAGGGAGTG 120

GFAP promoter

GCCAACTCCA TCACTAGGGG TTCCTACTAG TAACATATCC TGGTGTGGAG TAGGGGACGC 180

TGCTCTGACA GAGGCTCGGG GGCCTGAGCT GGCTCTGTGA GCTGGGGAGG AGGCAGACAG 240

CCAGGCCTTG TCTGCAAGCA GACCTGGCAG CATTGGGCTG GCCGCCCCCC AGGGCCTCCT 300

CTTCATGCCC AGTGAATGAC TCACCTTGGC ACAGACACAA TGTTCGGGGT GGGCACAGTG 360

CCTGCTTCCC GCCGCACCCC AGCCCCCCTC AAATGCCTTC CGAGAAGCCC ATTGAGCAGG 420

GGGCTTGCAT TGCACCCCAG CCTGACAGCC TGGCATCTTG GGATAAAAGC AGCACAGCCC 480

CCTAGGGGCT GCCCTTGCTG TGTGGCGCCA CCGGCGGTGG AGAACAAGGC TCTATTCAGC 540

CTGTGCCCAG GAAAGGGGAT CAGGGGATGC CCAGGCATGG ACAGTGGGTG GCAGGGGGGG 600

AGAGGAGGGC TGTCTGCTTC CCAGAAGTCC AAGGACACAA ATGGGTGAGG GGAGAGCTCT 660

CCCCATAGCT GGGCTGCGGC CCAACCCCAC CCCCTCAGGC TATGCCAGGG GGTGTTGCCA 720

GGGGCACCCG GGCATCGCCA GTCTAGCCCA CTCCTTCATA AAGCCCTCGT ATCCCAGGAG 780

CGAGCAGAGC CAGAGCAGGT TGGAGAGGAG ACGCATCACC TCTGCTGCTT GCCTAAAACA 840

GGTAAGTCCC ATTAATCTCC CTATCAGTGA TAGAGAAGGT CTGAAGAGTT TACTCCCTAT 900

CAGTGATAGA GATTAATTCC TCTACTAACC TTGTTCATCT TTTCTTTTTT TTTCTACAGG 960

Kozak Signal peptide

TCCTGGGTGA TTAACAGCTT AAGGCCGCCA CCATGCAGGT CTCCCGTGTG CTGGCCGCGC 1020

C2 domain

TGTGCGGCAT GCTACTCTGC GCCTCTGGCC TCTTCGCCGC GTCTGGTGAC CATATGCACG 1080

GATGTTCTGA GCCCCTGGGC CTGAAGAATA ACACAATTCC TGACAGCCAG ATGTCAGCCT 1140

CCAGCAGCTA CAAGACATGG AACCTGCGTG CTTTTGGCTG GTACCCCCAC TTGGGAAGGC 1200

TGGATAATCA GGGCAAGATC AATGCCTGGA CGGCTCAGAG CAACAGTGCC AAGGAATGGC 1260

TGCAGGTTGA CCTGGGCACT CAGAGGCAAG TGACAGGAAT CATCACCCAG GGGGCCCGTG 1320

ACTTTGGCCA CATCCAGTAT GTGGCGTCCT ACAAGGTAGC CCACAGTGAT GATGGTGTGC 1380

AGTGGACTGT ATATGAGGAG CAAGGAAGCA GCAAGGTCTT CCAGGGCAAC TTGGACAACA 1440

ACTCCCACAA GAAGAACATC TTCGAGAAAC CCTTCATGGC TCGCTACGTG CGTGTCCTTC 1500

linker

CAGTGTCCTG GCATAACCGC ATCACCCTGC GCCTGGAGCT GCTGGGCTGT AAGCTTGGCA 1560

CTGGAGGCTC TGGAGGCACT GGAGGCTCTG GAGGCACCGG TAGCACTAGC GGCGGAAGCG 1620

SNAP-tag

GCGGGACAGG TACGCGTGAC AAAGACTGCG AAATGAAGCG CACCACCCTG GATAGCCCTC 1680

TGGGCAAGCT GGAACTGTCT GGGTGCGAAC AGGGCCTGCA CCGTATCATC TTCCTGGGCA 1740

AAGGAACATC TGCCGCCGAC GCCGTGGAAG TGCCTGCCCC AGCCGCCGTG CTGGGCGGAC 1800

CAGAGCCACT GATGCAGGCC ACCGCCTGGC TCAACGCCTA CTTTCACCAG CCTGAGGCCA 1860

TCGAGGAGTT CCCTGTGCCA GCCCTGCACC ACCCAGTGTT CCAGCAGGAG AGCTTTACCC 1920

GCCAGGTGCT GTGGAAACTG CTGAAAGTGG TGAAGTTCGG AGAGGTCATC AGCTACAGCC 1980

ACCTGGCCGC CCTGGCCGGC AATCCCGCCG CCACCGCCGC CGTGAAAACC GCCCTGAGCG 2040

GAAATCCCGT GCCCATTCTG ATCCCCTGCC ACCGGGTGGT GCAGGGCGAC CTGGACGTGG 2100

GGGGCTACGA GGGCGGGCTC GCCGTGAAAG AGTGGCTGCT GGCCCACGAG GGCCACAGAC 2160

WPRE

TGGGCAAGCC TGGGCTGGGT GGATCCTAAT CAACCTCTGG ATTACAAAAT TTGTGAAAGA 2220

TTGACTGGTA TTCTTAACTA TGTTGCTCCT TTTACGCTAT GTGGATACGC TGCTTTAATG 2280

CCTTTGTATC ATGCTATTGC TTCCCGTATG GCTTTCATTT TCTCCTCCTT GTATAAATCC 2340

TGGTTGCTGT CTCTTTATGA GGAGTTGTGG CCCGTTGTCA GGCAACGTGG C GTGGTGTGC 2400

ACTGTGTTTG CTGACGCAAC CCCCACTGGT TGGGGCATTG CCACCACCTG TCAGCTCCTT 2460

TCCGGGACTT TCGCTTTCCC CCTCCCTATT GCCACGGCGG AACTCATCGC CGCCTGCCTT 2520

GCCCGCTGCT GGACAGGGGC TCGGCTGTTG GGCACTGACA ATTCCGTGGT GTTGTCGGGG 2580

AAATCATCGT CCTTTCCTTG GCTGCTCGCC TGTGTTGCCA CCTGGATTCT GCGCGGGACG 2640

TCCTTCTGCT ACGTCCCTTC GGCCCTCAAT CCAGCGGACC TTCCTTCCCG CGGCCTGCTG 2700

CCGGCTCTGC GGCCTCTTCC GCGTCTTCGC CTTCGCCCTC AGACGAGTCG GATCTCCCTT 2760

TGGGCCGCCT CCCCGCCTGA ATTCTGCATG TTTAAACATA CATACTTCTT TACATTCCAG 2820

ATATCTGCAT CAAACACCAT TGTCACACTC CATCGCGATC ACGCGAGCCG AACGAACAAA 2880

CCCAACAACA TGAAACTACC TAGCGCTTCT ATTCAGCATG ATGTCTTTCG TATACACAAA 2940

TTCGGTTCTA CAGGGTAACC TAGGCTGCAT GTTTAAACAT ACATACTTCT TTACATTCCA 3000

GATATCTGCA TCAAACACCA TTGTCACACT CCATCGCGAT CACGCGAGCC GAACGAACAA 3060

ACCCAACAAC ATGAAACTAC CTAGCGCTTC TATTCAGCAT GATGTCTTTC GTATACACAA 3120

ATTCGGTTCT ACAGGGTAAC CTAGGCTGCA TGTTTAAACA TACATACTTC TTTACATTCC 3180

AGATATCTGC ATCAAACACC ATTGTCACAC TCCATCGCGA TCACGCGAGC CGAACGAACA 3240

AACCCAACAA CATGAAACTA CCTAGCGCTT CTATTCAGCA TGATGTCTTT CGTATACACA 3300

SV40 poly(A)

AATTCGGTTC TACAGGGTAA CCTAGGCTGC AGGTACCTTC GAGCAGACAT GATAAGATAC 3360

ATTGATGAGT TTGGACAAAC CACAACTAGA ATGCAGTGAA AAAAATGCTT TATTTGTGAA 3420

ATTTGTGATG CTATTGCTTT ATTTGTAACC ATTATAAGCT GCAATAAACA AGTTAACAAC 3480

AACAATTGCA TTCATTTTAT GTTTCAGGTT CAGGGGGAGA TGTGGGAGGT TTTTTAAAGC 3540

AAGTAAAACC TCTACAAATG TGGTAAAATC AAGCTTAGGA ACCCCTAGTG ATGGAGTTGG 3600

CCACTCCCTC TCTGCGCGCT CGCTCGCTCA CTGAGGCCGG GCGACCAAAG GTCGCCCGAC 3660

GCCCGGGCTT TGCCCGGGCG GCCTCAGTGA GCGAGCGAGC GCGCAGAGAG GGAGTGGCCA 3720

AGCTAGCGGG CGATTAAGGA AAGGGCTAGA TCATTCTTGA AGACGAAAGG GCCTCGTGAT 3780

ACGCCTATTT TTATAGGTTA ATGTCATGAT AATAATGGTT TCTTAGACGT CAGGTGGCAC 3840
TTTTCGGGGA AATGTGCGCG GAACCCCTAT TTGTTTATTT TTCTAAATAC ATTCAAATAT 3900
GTATCCGCTC ATGAGACAAT AACCCTGATA AATGCTTCAA TAATATTGAA AAAGGAAGAG 3960
TATGAGTATT CAACATTTCC GTGTCGCCCT TATTCCCTTT TTTGCGGCAT TTTGCCTTCC 4020
TGTTTTTGCT CACCCAGAAA CGCTGGTGAA AGTAAAAGAT GCTGAAGATC AGTTGGGTGC 4080
ACGAGTGGGT TACATCGAAC TGGATCTCAA CAGCGGTAAG ATCCTTGAGA GTTTTCGCCC 4140
CGAAGAACGT TTTCCAATGA TGAGCACTTT TAAAGTTCTG CTATGTGGCG CGGTATTATC 4200
CCGTGTTGAC GCCGGGCAAG AGCAACTCGG TCGCCGCATA CACTATTCTC AGAATGACTT 4260
GGTTGAGTAC TCACCAGTCA CAGAAAAGCA TCTTACGGAT GGCATGACAG TAAGAGAATT 4320
ATGCAGTGCT GCCATAACCA TGAGTGATAA CACTGCGGCC AACTTACTTC TGACAACGAT 4380
CGGAGGACCG AAGGAGCTAA CCGCTTTTTT GCACAACATG GGGGATCATG TAACTCGCCT 4440
TGATCGTTGG GAACCGGAGC TGAATGAAGC CATACCAAAC GACGAGCGTG ACACCACGAT 4500
GCCTGTAGCA ATGGCAACAA CGTTGCGCAA ACTATTAACT GGCGAACTAC TTACTCTAGC 4560
TTCCCGGCAA CAATTAATAG ACTGGATGGA GGCGGATAAA GTTGCAGGAC CACTTCTGCG 4620
CTCGGCCCTT CCGGCTGGCT GGTTTATTGC TGATAAATCT GGAGCCGGTG AGCGTGGGTC 4680
TCGCGGTATC ATTGCAGCAC TGGGGCCAGA TGGTAAGCCC TCCCGTATCG TAGTTATCTA 4740
CACGACGGGG AGTCAGGCAA CTATGGATGA ACGAAATAGA CAGATCGCTG AGATAGGTGC 4800
CTCACTGATT AAGCATTGGT AACTGTCAGA CCAAGTTTAC TCATATATAC TTTAGATTGA 4860
TTTAAAACTT CATTTTTAAT TTAAAAGGAT CTAGGTGAAG ATCCTTTTTG ATAATCTCAT 4920
GACCAAAATC CCTTAACGTG AGTTTTCGTT CCACTGAGCG TCAGACCCCG TAGAAAAGAT 4980
CAAAGGATCT TCTTGAGATC CTTTTTTTCT GCGCGTAATC TGCTGCTTGC AAACAAAAAA 5040
ACCACCGCTA CCAGCGGTGG TTTGTTTGCC GGATCAAGAG CTACCAACTC TTTTTCCGAA 5100
GGTAACTGGC TTCAGCAGAG CGCAGATACC AAATACTGTT CTTCTAGTGT AGCCGTAGTT 5160
AGGCCACCAC TTCAAGAACT CTGTAGCACC GCCTACATAC CTCGCTCTGC TAATCCTGTT 5220
ACCAGTGGCT GCTGCCAGTG GCGATAAGTC GTGTCTTACC GGGTTGGACT CAAGACGATA 5280
GTTACCGGAT AAGGCGCAGC GGTCGGGCTG AACGGGGGGT TCGTGCACAC AGCCCAGCTT 5340
GGAGCGAACG ACCTACACCG AACTGAGATA CCTACAGCGT GAGCTATGAG AAAGCGCCAC 5400
GCTTCCCGAA GGGAGAAAGG CGGACAGGTA TCCGGTAAGC GGCAGGGTCG GAACAGGAGA 5460
GCGCACGAGG GAGCTTCCAG GGGGAAACGC CTGGTATCTT TATAGTCCTG TCGGGTTTCG 5520
CCACCTCTGA CTTGAGCGTC GATTTTTGTG ATGCTCGTCA GGGGGGCGGA GCCTATGGAA 5580
AAACGCCAGC AACGCGGCCT TTTTACGGTT CCTGGCCTTT TGCTGGCCTT TTGCTCACAT 5640
GTAATAAACA CACACACACC AACAACCGTG GTTGGTTGTT GTGTTGGTTT ATTCTCGAG 5699

Sequence Name: pAAV-C2m2-mKate

Length: 5852

TTGGCCACTC CCTCTCTGCG CGCTCGCTCG CTCACTGAGG CCGGGCGACC AAAGGTCGCC 60

CGACGCCCGG GCTTTGCCCG GGCGGCCTCA GTGAGCGAGC GAGCGCGCAG AGAGGGAGTG 120

GFAP promoter

GCCAACTCCA TCACTAGGGG TTCCTACTAG TAACATATCC TGGTGTGGAG TAGGGGACGC 180

TGCTCTGACA GAGGCTCGGG GGCCTGAGCT GGCTCTGTGA GCTGGGGAGG AGGCAGACAG 240

CCAGGCCTTG TCTGCAAGCA GACCTGGCAG CATTGGGCTG GCCGCCCCCC AGGGCCTCCT 300

CTTCATGCCC AGTGAATGAC TCACCTTGGC ACAGACACAA TGTTCGGGGT GGGCACAGTG 360

CCTGCTTCCC GCCGCACCCC AGCCCCCCTC AAATGCCTTC CGAGAAGCCC ATTGAGCAGG 420

GGGCTTGCAT TGCACCCCAG CCTGACAGCC TGGCATCTTG GGATAAAAGC AGCACAGCCC 480

CCTAGGGGCT GCCCTTGCTG TGTGGCGCCA CCGGCGGTGG AGAACAAGGC TCTATTCAGC 540

CTGTGCCCAG GAAAGGGGAT CAGGGGATGC CCAGGCATGG ACAGTGGGTG GCAGGGGGGG 600

AGAGGAGGGC TGTCTGCTTC CCAGAAGTCC AAGGACACAA ATGGGTGAGG GGAGAGCTCT 660

CCCCATAGCT GGGCTGCGGC CCAACCCCAC CCCCTCAGGC TATGCCAGGG GGTGTTGCCA 720

GGGGCACCCG GGCATCGCCA GTCTAGCCCA CTCCTTCATA AAGCCCTCGT ATCCCAGGAG 780

CGAGCAGAGC CAGAGCAGGT TGGAGAGGAG ACGCATCACC TCTGCTGCTT GCCTAAAACA 840

GGTAAGTCCC ATTAATCTCC CTATCAGTGA TAGAGAAGGT CTGAAGAGTT TACTCCCTAT 900

CAGTGATAGA GATTAATTCC TCTACTAACC TTGTTCATCT TTTCTTTTTT TTTCTACAGG 960

Kozak **Signal peptide**

TCCTGGGTGA TTAACAGCTT AAGGCCGCCA CCATGCAGGT CTCCCGTGTG CTGGCCGCGC 1020

C2 domain

TGTGCGGCAT GCTACTCTGC GCCTCTGGCC TCTTCGCCGC GTCTGGTGAC CATATGCACG 1080

GATGTTCTGA GCCCCTGGGC CTGAAGAATA ACACAATTCC TGACAGCCAG ATGTCAGCCT 1140

CCAGCAGCTA CAATACATGG AACCTGCGTG CTTTTGGCTG GTACCCCCAC TTGGGAAGGC 1200

TGGATAATCA GGGCAATATC AATGCCTGGA CGGCTCAGAG CAACAGTGCC AAGGAATGGC 1260

TGCAGGTTGA CCTGGGCACT CAGAGGCAAG TGACAGGAAT CATCACCCAG GGGGCCCGTG 1320

ACTTTGGCCA CATCCAGTAT GTGGCGTCCT ACAAGGTAGC CCACAGTGAT GATGGTGTGC 1380

AGTGGACTGT ATATGAGGAG CAAGGAAGCA GCAAGGTCTT CCAGGGCAAC TTGGACAACA 1440

ACTCCCACAA GAAGAACATC TTCGAGAAAC CCTTCATGGC TCGCTACGTG CGTGTCCTTC 1500

linker

CAGTGTCCTG GCATAACCGC ATCACCCTGC GCCTGGAGCT GCTGGGCTGT AAGCTTGGCA 1560

CTGGAGGCTC TGGAGGCACT GGAGGCTCTG GAGGCACCGG TAGCACTAGC GGCGGAAGCG 1620

mKate

GCGGGACAGG TACGCGTGTG AGCGAGCTGA TTAAGGAGAA CATGCACATG AAGCTGTACA 1680

TGGAGGGCAC CGTGAACAAC CACCACTTCA AGTGCACATC CGAGGGCGAA GGCAAGCCCT 1740

ACGAGGGCAC CCAGACCATG AGAATCAAGG TCGTCGAGGG CGGCCCTCTC CCCTTCGCCT 1800

TCGACATCCT GGCTACCAGC TTCATGTACG GCAGCAAAAC CTTCATCAAC CACCCTCAGG 1860

GCATCCCCGA CTTCTTTAAG CAGTCCTTCC CTGAGGGCTT CACATGGGAG AGAGTCACCA 1920

CATACGAAGA CGGGGGCGTG CTGACCGCTA CCCAGGACAC CAGCCTCCAG GACGGCTGCC 1980

TCATCTACAA CGTCAAGATT AGAGGGGTGA ACTTCCCAGC CAACGGCCCT GTGATGCAGA 2040

AGAAAACACT CGGCTGGGAG GCCTCCACCG AGACGCTGTA CCCCGCTGAC GGCGGCCTGG 2100

AAGGCGCATG TGACATGGCC CTGAAGCTCG TGGGCGGGGG CCACCTGATC TGCAACTTGG 2160

AGACCACATA CAGATCCAAG AAACCCGCTA AGAACCTCAA GATGCCCGGC GTCTACAACG 2220

TGGACAGGAG ACTGGAAAGA ATCAAGGAGG CCGACAATGA GACCTACGTC GAGCAGCACG 2280

AGGTGGCTGT GGCCAGATAC TCTACTGGTG GCGCTGGTGA TGGAGGTAAA TGAGGATCCT 2340

WPRE

AATCAACCTC TGGATTACAA AATTTGTGAA AGATTGACTG GTATTCTTAA CTATGTTGCT 2400

CCTTTTACGC TATGTGGATA CGCTGCTTTA ATGCCTTTGT ATCATGCTAT TGCTTCCCGT 2460

ATGGCTTTCA TTTTCTCCTC CTTGTATAAA TCCTGGTTGC TGTCTCTTTA TGAGGAGTTG 2520

TGGCCCGTTG TCAGGCAACG TGGCGTGGTG TGCACTGTGT TTGCTGACGC AACCCCCACT 2580

GGTTGGGGCA TTGCCACCAC CTGTCAGCTC CTTTCCGGGA CTTTCGCTTT CCCCCTCCCT 2640

ATTGCCACGG CGGAACTCAT CGCCGCCTGC CTTGCCCGCT GCTGGACAGG GGCTCGGCTG 2700

TTGGGCACTG ACAATTCCGT GGTGTTGTCG GGGAAATCAT CGTCCTTTCC TTGGCTGCTC 2760

GCCTGTGTTG CCACCTGGAT TCTGCGCGGG ACGTCCTTCT GCTACGTCCC TTCGGCCCTC 2820

AATCCAGCGG ACCTTCCTTC CCGCGGCCTG CTGCCGGCTC TGCGGCCTCT TCCGCGTCTT 2880

CGCCTTCGCC CTCAGACGAG TCGGATCTCC CTTTGGGCCG CCTCCCCGCC TGAATTCTGC 2940

ATGTTTAAAC ATACATACTT CTTTACATTC CAGATATCTG CATCAAACAC CATTGTCACA 3000

CTCCATCGCG ATCACGCGAG CCGAACGAAC AAACCCAACA ACATGAAACT ACCTAGCGCT 3060

TCTATTCAGC ATGATGTCTT TCGTATACAC AAATTCGGTT CTACAGGGTA ACCTAGGCTG 3120

CATGTTTAAA CATACATACT TCTTTACATT CCAGATATCT GCATCAAACA CCATTGTCAC 3180

ACTCCATCGC GATCACGCGA GCCGAACGAA CAAACCCAAC AACATGAAAC TACCTAGCGC 3240

TTCTATTCAG CATGATGTCT TTCGTATACA CAAATTCGGT TCTACAGGGT AACCTAGGCT 3300

GCATGTTTAA ACATACATAC TTCTTTACAT TCCAGATATC TGCATCAAAC ACCATTGTCA 3360

CACTCCATCG CGATCACGCG AGCCGAACGA ACAAACCCAA CAACATGAAA CTACCTAGCG 3420

CTTCTATTCA GCATGATGTC TTTCGTATAC ACAAATTCGG TTCTACAGGG TAACCTAGGC 3480

SV40poly(A)

TGCAGGTACC TTCGAGCAGA CATGATAAGA TACATTGATG AGTTTGGACA AACCACAACT 3540

AGAATGCAGT GAAAAAAATG CTTTATTTGT GAAATTTGTG ATGCTATTGC TTTATTTGTA 3600

ACCATTATAA GCTGCAATAA ACAAGTTAAC AACAACAATT GCATTCATTT TATGTTTCAG 3660

GTTCAGGGGG AGATGTGGGA GGTTTTTTAA AGCAAGTAAA ACCTCTACAA ATGTGGTAAA 3720

ATCAAGCTTA GGAACCCCTA GTGATGGAGT TGGCCACTCC CTCTCTGCGC GCTCGCTCGC 3780

TCACTGAGGC CGGGCGACCA AAGGTCGCCC GACGCCCGGG CTTTGCCCGG GCGGCCTCAG 3840
TGAGCGAGCG AGCGCGCAGA GAGGGAGTGG CCAAGCTAGC GGGCGATTAA GGAAAGGGCT 3900
AGATCATTCT TGAAGACGAA AGGGCCTCGT GATACGCCTA TTTTTATAGG TTAATGTCAT 3960
GATAATAATG GTTTCTTAGA CGTCAGGTGG CACTTTTCGG GGAAATGTGC GCGGAACCCC 4020
TATTTGTTTA TTTTTCTAAA TACATTCAAA TATGTATCCG CTCATGAGAC AATAACCCTG 4080
ATAAATGCTT CAATAATATT GAAAAAGGAA GAGTATGAGT ATTCAACATT TCCGTGTCGC 4140
CCTTATTCCC TTTTTTGCGG CATTTTGCCT TCCTGTTTTT GCTCACCCAG AAACGCTGGT 4200
GAAAGTAAAA GATGCTGAAG ATCAGTTGGG TGCACGAGTG GGTTACATCG AACTGGATCT 4260
CAACAGCGGT AAGATCCTTG AGAGTTTTCG CCCCGAAGAA CGTTTTCCAA TGATGAGCAC 4320
TTTTAAAGTT CTGCTATGTG GCGCGGTATT ATCCCGTGTT GACGCCGGGC AAGAGCAACT 4380
CGGTCGCCGC ATACACTATT CTCAGAATGA CTTGGTTGAG TACTCACCAG TCACAGAAAA 4440
GCATCTTACG GATGGCATGA CAGTAAGAGA ATTATGCAGT GCTGCCATAA CCATGAGTGA 4500
TAACACTGCG GCCAACTTAC TTCTGACAAC GATCGGAGGA CCGAAGGAGC TAACCGCTTT 4560
TTTGCACAAC ATGGGGGATC ATGTAACTCG CCTTGATCGT TGGGAACCGG AGCTGAATGA 4620
AGCCATACCA AACGACGAGC GTGACACCAC GATGCCTGTA GCAATGGCAA CAACGTTGCG 4680
CAAACTATTA ACTGGCGAAC TACTTACTCT AGCTTCCCGG CAACAATTAA TAGACTGGAT 4740
GGAGGCGGAT AAAGTTGCAG GACCACTTCT GCGCTCGGCC CTTCCGGCTG GCTGGTTTAT 4800
TGCTGATAAA TCTGGAGCCG GTGAGCGTGG GTCTCGCGGT ATCATTGCAG CACTGGGGCC 4860
AGATGGTAAG CCCTCCCGTA TCGTAGTTAT CTACACGACG GGGAGTCAGG CAACTATGGA 4920
TGAACGAAAT AGACAGATCG CTGAGATAGG TGCCTCACTG ATTAAGCATT GGTAACTGTC 4980
AGACCAAGTT TACTCATATA TACTTTAGAT TGATTTAAAA CTTCATTTTT AATTTAAAAG 5040
GATCTAGGTG AAGATCCTTT TTGATAATCT CATGACCAAA ATCCCTTAAC GTGAGTTTTC 5100
GTTCCACTGA GCGTCAGACC CCGTAGAAAA GATCAAAGGA TCTTCTTGAG ATCCTTTTTT 5160
TCTGCGCGTA ATCTGCTGCT TGCAAACAAA AAAACCACCG CTACCAGCGG TGGTTTGTTT 5220
GCCGGATCAA GAGCTACCAA CTCTTTTTCC GAAGGTAACT GGCTTCAGCA GAGCGCAGAT 5280
ACCAAATACT GTTCTTCTAG TGTAGCCGTA GTTAGGCCAC CACTTCAAGA ACTCTGTAGC 5340
ACCGCCTACA TACCTCGCTC TGCTAATCCT GTTACCAGTG GCTGCTGCCA GTGGCGATAA 5400
GTCGTGTCTT ACCGGGTTGG ACTCAAGACG ATAGTTACCG GATAAGGCGC AGCGGTCGGG 5460
CTGAACGGGG GGTTCGTGCA CACAGCCCAG CTTGGAGCGA ACGACCTACA CCGAACTGAG 5520
ATACCTACAG CGTGAGCTAT GAGAAAGCGC CACGCTTCCC GAAGGGAGAA AGGCGGACAG 5580
GTATCCGGTA AGCGGCAGGG TCGGAACAGG AGAGCGCACG AGGGAGCTTC CAGGGGGAAA 5640
CGCCTGGTAT CTTTATAGTC CTGTCGGGTT TCGCCACCTC TGACTTGAGC GTCGATTTTT 5700
GTGATGCTCG TCAGGGGGGC GGAGCCTATG GAAAAACGCC AGCAACGCGG CCTTTTTACG 5760
GTTCCTGGCC TTTTGCTGGC CTTTTGCTCA CATGTAATAA ACACACACAC ACCAACAACC 5820
GTGGTTGGTT GTTGTGTTGG TTTATTCTCG AG 5852

Sequence Name: pAAV-C2m2-SNAP

Length: 5699

TTGGCCACTC CCTCTCTGCG CGCTCGCTCG CTCACTGAGG CCGGGCGACC AAAGGTCGCC 60

CGACGCCCGG GCTTTGCCCG GGCGGCCTCA GTGAGCGAGC GAGCGCGCAG AGAGGGAGTG 120

GFAP promoter

GCCAACTCCA TCACTAGGGG TTCCTACTAG TAACATATCC TGGTGTGGAG TAGGGGACGC 180

TGCTCTGACA GAGGCTCGGG GGCCTGAGCT GGCTCTGTGA GCTGGGGAGG AGGCAGACAG 240

CCAGGCCTTG TCTGCAAGCA GACCTGGCAG CATTGGGCTG GCCGCCCCCC AGGGCCTCCT 300

CTTCATGCCC AGTGAATGAC TCACCTTGGC ACAGACACAA TGTTCGGGGT GGGCACAGTG 360

CCTGCTTCCC GCCGCACCCC AGCCCCCCTC AAATGCCTTC CGAGAAGCCC ATTGAGCAGG 420

GGGCTTGCAT TGCACCCCAG CCTGACAGCC TGGCATCTTG GGATAAAAGC AGCACAGCCC 480

CCTAGGGGCT GCCCTTGCTG TGTGGCGCCA CCGGCGGTGG AGAACAAGGC TCTATTCAGC 540

CTGTGCCCAG GAAAGGGGAT CAGGGGATGC CCAGGCATGG ACAGTGGGTG GCAGGGGGGG 600

AGAGGAGGGC TGTCTGCTTC CCAGAAGTCC AAGGACACAA ATGGGTGAGG GGAGAGCTCT 660

CCCCATAGCT GGGCTGCGGC CCAACCCCAC CCCCTCAGGC TATGCCAGGG GGTGTTGCCA 720

GGGGCACCCG GGCATCGCCA GTCTAGCCCA CTCCTTCATA AAGCCCTCGT ATCCCAGGAG 780

CGAGCAGAGC CAGAGCAGGT TGGAGAGGAG ACGCATCACC TCTGCTGCTT GCCTAAAACA 840

GGTAAGTCCC ATTAATCTCC CTATCAGTGA TAGAGAAGGT CTGAAGAGTT TACTCCCTAT 900

CAGTGATAGA GATTAATTCC TCTACTAACC TTGTTCATCT TTTCTTTTTT TTTCTACAGG 960

Kozak **Signal peptide**

TCCTGGGTGA TTAACAGCTT AAGGCCGCCA CCATGCAGGT CTCCCGTGTG CTGGCCGCGC 1020

C2 domain

TGTGCGGCAT GCTACTCTGC GCCTCTGGCC TCTTCGCCGC GTCTGGTGAC CATATGCACG 1080

GATGTTCTGA GCCCCTGGGC CTGAAGAATA ACACAATTCC TGACAGCCAG ATGTCAGCCT 1140

CCAGCAGCTA CAATACATGG AACCTGCGTG CTTTTGGCTG GTACCCCCAC TTGGGAAGGC 1200

TGGATAATCA GGGCAATATC AATGCCTGGA CGGCTCAGAG CAACAGTGCC AAGGAATGGC 1260

TGCAGGTTGA CCTGGGCACT CAGAGGCAAG TGACAGGAAT CATCACCCAG GGGGCCCGTG 1320

ACTTTGGCCA CATCCAGTAT GTGGCGTCCT ACAAGGTAGC CCACAGTGAT GATGGTGTGC 1380

AGTGGACTGT ATATGAGGAG CAAGGAAGCA GCAAGGTCTT CCAGGGCAAC TTGGACAACA 1440

ACTCCCACAA GAAGAACATC TTCGAGAAAC CCTTCATGGC TCGCTACGTG CGTGTCCTTC 1500

linker

CAGTGTCCTG GCATAACCGC ATCACCCTGC GCCTGGAGCT GCTGGGCTGT AAGCTTGGCA 1560

CTGGAGGCTC TGGAGGCACT GGAGGCTCTG GAGGCACCGG TAGCACTAGC GGCGGAAGCG 1620

SNAP-tag

GCGGGACAGG TACGCGTGAC AAAGACTGCG AAATGAAGCG CACCACCCTG GATAGCCCTC 1680

TGGGCAAGCT GGAACTGTCT GGGTGCGAAC AGGGCCTGCA CCGTATCATC TTCCTGGGCA 1740

AAGGAACATC TGCCGCCGAC GCCGTGGAAG TGCCTGCCCC AGCCGCCGTG CTGGGCGGAC 1800

CAGAGCCACT GATGCAGGCC ACCGCCTGGC TCAACGCCTA CTTTCACCAG CCTGAGGCCA 1860

TCGAGGAGTT CCCTGTGCCA GCCCTGCACC ACCCAGTGTT CCAGCAGGAG AGCTTTACCC 1920

GCCAGGTGCT GTGGAAACTG CTGAAAGTGG TGAAGTTCGG AGAGGTCATC AGCTACAGCC 1980

ACCTGGCCGC CCTGGCCGGC AATCCCGCCG CCACCGCCGC CGTGAAAACC GCCCTGAGCG 2040

GAAATCCCGT GCCCATTCTG ATCCCCTGCC ACCGGGTGGT GCAGGGCGAC CTGGACGTGG 2100

GGGGCTACGA GGGCGGGCTC GCCGTGAAAG AGTGGCTGCT GGCCCACGAG GGCCACAGAC 2160

WPRE

TGGGCAAGCC TGGGCTGGGT GGATCCTAAT CAACCTCTGG ATTACAAAAT TTGTGAAAGA 2220

TTGACTGGTA TTCTTAACTA TGTTGCTCCT TTTACGCTAT GTGGATACGC TGCTTTAATG 2280

CCTTTGTATC ATGCTATTGC TTCCCGTATG GCTTTCATTT TCTCCTCCTT GTATAAATCC 2340

TGGTTGCTGT CTCTTTATGA GGAGTTGTGG CCCGTTGTCA GGCAACGTGG C GTGGTGTGC 2400

ACTGTGTTTG CTGACGCAAC CCCCACTGGT TGGGGCATTG CCACCACCTG TCAGCTCCTT 2460

TCCGGGACTT TCGCTTTCCC CCTCCCTATT GCCACGGCGG AACTCATCGC CGCCTGCCTT 2520

GCCCGCTGCT GGACAGGGGC TCGGCTGTTG GGCACTGACA ATTCCGTGGT GTTGTCGGGG 2580

AAATCATCGT CCTTTCCTTG GCTGCTCGCC TGTGTTGCCA CCTGGATTCT GCGCGGGACG 2640

TCCTTCTGCT ACGTCCCTTC GGCCCTCAAT CCAGCGGACC TTCCTTCCCG CGGCCTGCTG 2700

CCGGCTCTGC GGCCTCTTCC GCGTCTTCGC CTTCGCCCTC AGACGAGTCG GATCTCCCTT 2760

TGGGCCGCCT CCCCGCCTGA ATTCTGCATG TTTAAACATA CATACTTCTT TACATTCCAG 2820

ATATCTGCAT CAAACACCAT TGTCACACTC CATCGCGATC ACGCGAGCCG AACGAACAAA 2880

CCCAACAACA TGAAACTACC TAGCGCTTCT ATTCAGCATG ATGTCTTTCG TATACACAAA 2940

TTCGGTTCTA CAGGGTAACC TAGGCTGCAT GTTTAAACAT ACATACTTCT TTACATTCCA 3000

GATATCTGCA TCAAACACCA TTGTCACACT CCATCGCGAT CACGCGAGCC GAACGAACAA 3060

ACCCAACAAC ATGAAACTAC CTAGCGCTTC TATTCAGCAT GATGTCTTTC GTATACACAA 3120

ATTCGGTTCT ACAGGGTAAC CTAGGCTGCA TGTTTAAACA TACATACTTC TTTACATTCC 3180

AGATATCTGC ATCAAACACC ATTGTCACAC TCCATCGCGA TCACGCGAGC CGAACGAACA 3240

AACCCAACAA CATGAAACTA CCTAGCGCTT CTATTCAGCA TGATGTCTTT CGTATACACA 3300

SV40 poly(A)

AATTCGGTTC TACAGGGTAA CCTAGGCTGC AGGTACCTTC GAGCAGACAT GATAAGATAC 3360

ATTGATGAGT TTGGACAAAC CACAACTAGA ATGCAGTGAA AAAAATGCTT TATTTGTGAA 3420

ATTTGTGATG CTATTGCTTT ATTTGTAACC ATTATAAGCT GCAATAAACA AGTTAACAAC 3480

AACAATTGCA TTCATTTTAT GTTTCAGGTT CAGGGGGAGA TGTGGGAGGT TTTTTAAAGC 3540

AAGTAAAACC TCTACAAATG TGGTAAAATC AAGCTTAGGA ACCCCTAGTG ATGGAGTTGG 3600

CCACTCCCTC TCTGCGCGCT CGCTCGCTCA CTGAGGCCGG GCGACCAAAG GTCGCCCGAC 3660

GCCCGGGCTT TGCCCGGGCG GCCTCAGTGA GCGAGCGAGC GCGCAGAGAG GGAGTGGCCA 3720

AGCTAGCGGG CGATTAAGGA AAGGGCTAGA TCATTCTTGA AGACGAAAGG GCCTCGTGAT 3780

ACGCCTATTT TTATAGGTTA ATGTCATGAT AATAATGGTT TCTTAGACGT CAGGTGGCAC 3840
TTTTCGGGGA AATGTGCGCG GAACCCCTAT TTGTTTATTT TTCTAAATAC ATTCAAATAT 3900
GTATCCGCTC ATGAGACAAT AACCCTGATA AATGCTTCAA TAATATTGAA AAAGGAAGAG 3960
TATGAGTATT CAACATTTCC GTGTCGCCCT TATTCCCTTT TTTGCGGCAT TTTGCCTTCC 4020
TGTTTTTGCT CACCCAGAAA CGCTGGTGAA AGTAAAAGAT GCTGAAGATC AGTTGGGTGC 4080
ACGAGTGGGT TACATCGAAC TGGATCTCAA CAGCGGTAAG ATCCTTGAGA GTTTTCGCCC 4140
CGAAGAACGT TTTCCAATGA TGAGCACTTT TAAAGTTCTG CTATGTGGCG CGGTATTATC 4200
CCGTGTTGAC GCCGGGCAAG AGCAACTCGG TCGCCGCATA CACTATTCTC AGAATGACTT 4260
GGTTGAGTAC TCACCAGTCA CAGAAAAGCA TCTTACGGAT GGCATGACAG TAAGAGAATT 4320
ATGCAGTGCT GCCATAACCA TGAGTGATAA CACTGCGGCC AACTTACTTC TGACAACGAT 4380
CGGAGGACCG AAGGAGCTAA CCGCTTTTTT GCACAACATG GGGGATCATG TAACTCGCCT 4440
TGATCGTTGG GAACCGGAGC TGAATGAAGC CATACCAAAC GACGAGCGTG ACACCACGAT 4500
GCCTGTAGCA ATGGCAACAA CGTTGCGCAA ACTATTAACT GGCGAACTAC TTACTCTAGC 4560
TTCCCGGCAA CAATTAATAG ACTGGATGGA GGCGGATAAA GTTGCAGGAC CACTTCTGCG 4620
CTCGGCCCTT CCGGCTGGCT GGTTTATTGC TGATAAATCT GGAGCCGGTG AGCGTGGGTC 4680
TCGCGGTATC ATTGCAGCAC TGGGGCCAGA TGGTAAGCCC TCCCGTATCG TAGTTATCTA 4740
CACGACGGGG AGTCAGGCAA CTATGGATGA ACGAAATAGA CAGATCGCTG AGATAGGTGC 4800
CTCACTGATT AAGCATTGGT AACTGTCAGA CCAAGTTTAC TCATATATAC TTTAGATTGA 4860
TTTAAAACTT CATTTTTAAT TTAAAAGGAT CTAGGTGAAG ATCCTTTTTG ATAATCTCAT 4920
GACCAAAATC CCTTAACGTG AGTTTTCGTT CCACTGAGCG TCAGACCCCG TAGAAAAGAT 4980
CAAAGGATCT TCTTGAGATC CTTTTTTTCT GCGCGTAATC TGCTGCTTGC AAACAAAAAA 5040
ACCACCGCTA CCAGCGGTGG TTTGTTTGCC GGATCAAGAG CTACCAACTC TTTTTCCGAA 5100
GGTAACTGGC TTCAGCAGAG CGCAGATACC AAATACTGTT CTTCTAGTGT AGCCGTAGTT 5160
AGGCCACCAC TTCAAGAACT CTGTAGCACC GCCTACATAC CTCGCTCTGC TAATCCTGTT 5220
ACCAGTGGCT GCTGCCAGTG GCGATAAGTC GTGTCTTACC GGGTTGGACT CAAGACGATA 5280
GTTACCGGAT AAGGCGCAGC GGTCGGGCTG AACGGGGGGT TCGTGCACAC AGCCCAGCTT 5340
GGAGCGAACG ACCTACACCG AACTGAGATA CCTACAGCGT GAGCTATGAG AAAGCGCCAC 5400
GCTTCCCGAA GGGAGAAAGG CGGACAGGTA TCCGGTAAGC GGCAGGGTCG GAACAGGAGA 5460
GCGCACGAGG GAGCTTCCAG GGGGAAACGC CTGGTATCTT TATAGTCCTG TCGGGTTTCG 5520
CCACCTCTGA CTTGAGCGTC GATTTTTGTG ATGCTCGTCA GGGGGGCGGA GCCTATGGAA 5580
AAACGCCAGC AACGCGGCCT TTTTACGGTT CCTGGCCTTT TGCTGGCCTT TTGCTCACAT 5640
GTAATAAACA CACACACACC AACAACCGTG GTTGGTTGTT GTGTTGGTTT ATTCTCGAG 5699
