# Supplementary material for: Genetically encoded phosphatidylserine biosensor for in vitro, ex vivo and in vivo labelling
Source: Cell Mol Biol Lett. 2023 Jul 27;28:59. doi: 10.1186/s11658-023-00472-7 (PMC10373266; doi:10.1186/s11658-023-00472-7)
Supplement: Supplementary file 3 — Additional file 3. CellProfiler pipeline. [file 11658_2023_472_MOESM3_ESM.pdf]

Additional File 3 for

Genetically encoded phosphatidylserine biosensor for in vitro, ex vivo and in vivo labelling

Eimina Dirvelyte[1], Daina Bujanauskiene[1,2], Evelina Jankaityte[1,3], Neringa Daugelaviciene[1], Ugne Kisieliute[2], Igor Nagula[1], Rima Budvytyte[1,3], Urte Neniskyte[1,2]*

[1]VU LSC-EMBL Partnership for Genome Editing Technologies, Life Sciences Center, Vilnius University, Vilnius, Lithuania

[2]Institute of Bioscience, Life Sciences Center, Vilnius University, Vilnius, Lithuania

[3]Institute of Biochemistry, Life Sciences Center, Vilnius University, Vilnius, Lithuania

*Corresponding author: urte.neniskyte@gmc.vu.lt

This file includes:

CellProfiler pipeline

CellProfiler pipeline

Version:5

DateRevision:421

GitHash:

ModuleCount:9

HasImagePlaneDetails:False

Images:[module_num:1|svn_version:'Unknown'|variable_revision_number:2|show_window:False|notes:['To begin creating your project, use the Images module to compile a list of files and/or folders that you want to analyze. You can also specify a set of rules to include only the desired files in your selected folders.']|batch_state:array([], dtype=uint8)|enabled:True|wants_pause:False]

:

Filter images?:Images only

Select the rule criteria:and (extension does isimage) (directory doesnot containregexp "[\\\\/]\\.")

Metadata:[module_num:2|svn_version:'Unknown'|variable_revision_number:6|show_window:False|notes:['The Metadata module optionally allows you to extract information describing your images (i.e, metadata) which will be stored along with your measurements. This information can be contained in the file name and/or location, or in an external file.']|batch_state:array([], dtype=uint8)|enabled:True|wants_pause:False]

Extract metadata?:Yes

Metadata data type:Text

Metadata types:{}

Extraction method count:1

Metadata extraction method:Extract from file/folder names

Metadata source:File name

Regular expression to extract from file name:^(?P<Experiment>.*)_(?P<Protein>.{1,8})-(?P<Condition>.{1,4})--(?P<Well>.{1,2})-(?P<ROI>.{1,2})

Regular expression to extract from folder name:(?P<Date>[0-9]{4}_[0-9]{2}_[0-9]{2})$

Extract metadata from:All images

Select the filtering criteria:and (file does contain "")

Metadata file location:Elsewhere...|

Match file and image metadata:[]

Use case insensitive matching?:No

Metadata file name:None

Does cached metadata exist?:No

NamesAndTypes:[module_num:3|svn_version:'Unknown'|variable_revision_number:8|show_window:False|notes:['The NamesAndTypes module allows you to assign a meaningful name to each image by which other modules will refer to it.']|batch_state:array([], dtype=uint8)|enabled:True|wants_pause:False]

Assign a name to:All images

Select the image type:Color image

Name to assign these images:Image

Match metadata:[]

Image set matching method:Order

Set intensity range from:Image metadata

Assignments count:1

Single images count:0

Maximum intensity:255.0

Process as 3D?:No

Relative pixel spacing in X:1.0

Relative pixel spacing in Y:1.0

Relative pixel spacing in Z:1.0

Select the rule criteria:and (file does contain "")

Name to assign these images:DNA

Name to assign these objects:Cell

Select the image type:Grayscale image

Set intensity range from:Image metadata

Maximum intensity:255.0

Groups:[module_num:4|svn_version:'Unknown'|variable_revision_number:2|show_window:False|notes:['The Groups module optionally allows you to split your list of images into image subsets (groups) which will be processed independently of each other. Examples of groupings include screening batches, microtiter plates, time-lapse movies, etc.']|batch_state:array([], dtype=uint8)|enabled:True|wants_pause:False]

Do you want to group your images?:No

grouping metadata count:1

Metadata category:None

ColorToGray:[module_num:5|svn_version:'Unknown'|variable_revision_number:4|show_window:True|notes:[]|batch_state:array([], dtype=uint8)|enabled:True|wants_pause:False]

Select the input image:Image

Conversion method:Split

Image type:Channels

Name the output image:OrigGray

Relative weight of the red channel:1.0

Relative weight of the green channel:1.0

Relative weight of the blue channel:1.0

Convert red to gray?:Yes

Name the output image:OrigRed

Convert green to gray?:Yes

Name the output image:OrigGreen

Convert blue to gray?:Yes

Name the output image:OrigBlue

Convert hue to gray?:Yes

Name the output image:OrigHue

Convert saturation to gray?:Yes

Name the output image:OrigSaturation

Convert value to gray?:Yes

Name the output image:OrigValue

Channel count:2

Channel number:1

Relative weight of the channel:1.0

Image name:DAPI

Channel number:2

Relative weight of the channel:1.0

Image name:589

IdentifyPrimaryObjects:[module_num:6|svn_version:'Unknown'|variable_revision_number:15|show_window:True|notes:[]|batch_state:array([], dtype=uint8)|enabled:True|wants_pause:False]

Select the input image:DAPI

Name the primary objects to be identified:Nuclei

Typical diameter of objects, in pixel units (Min,Max):10,100

Discard objects outside the diameter range?:Yes

Discard objects touching the border of the image?:Yes

Method to distinguish clumped objects:Shape

Method to draw dividing lines between clumped objects:Shape

Size of smoothing filter:50

Suppress local maxima that are closer than this minimum allowed distance:7.0

Speed up by using lower-resolution image to find local maxima?:Yes

Fill holes in identified objects?:After declumping only

Automatically calculate size of smoothing filter for declumping?:Yes

Automatically calculate minimum allowed distance between local maxima?:Yes

Handling of objects if excessive number of objects identified:Continue

Maximum number of objects:500

Use advanced settings?:Yes

Threshold setting version:12

Threshold strategy:Global

Thresholding method:Minimum Cross-Entropy

Threshold smoothing scale:2.7

Threshold correction factor:1.0

Lower and upper bounds on threshold:0.0,1.0

Manual threshold:0.0

Select the measurement to threshold with:None

Two-class or three-class thresholding?:Two classes

Log transform before thresholding?:No

Assign pixels in the middle intensity class to the foreground or the background?:Foreground

Size of adaptive window:50

Lower outlier fraction:0.05

Upper outlier fraction:0.05

Averaging method:Mode

Variance method:Standard deviation

# of deviations:2.0

Thresholding method:Minimum Cross-Entropy

MeasureImageIntensity:[module_num:7|svn_version:'Unknown'|variable_revision_number:4|show_window:True|notes:[]|batch_state:array([], dtype=uint8)|enabled:True|wants_pause:False]

Select images to measure:589

Measure the intensity only from areas enclosed by objects?:No

Select input object sets:

Calculate custom percentiles:No

Specify percentiles to measure:10,90

CalculateMath:[module_num:8|svn_version:'Unknown'|variable_revision_number:3|show_window:True|notes:[]|batch_state:array([], dtype=uint8)|enabled:True|wants_pause:False]

Name the output measurement:IntensityPerCell

Operation:Divide

Select the numerator measurement type:Image

Select the numerator objects:None

Select the numerator measurement:FileName_Image

Multiply the above operand by:1.0

Raise the power of above operand by:1.0

Select the denominator measurement type:Image

Select the denominator objects:Nuclei

Select the denominator measurement:Count_Nuclei

Multiply the above operand by:1.0

Raise the power of above operand by:1.0

Take log10 of result?:No

Multiply the result by:1.0

Raise the power of result by:1.0

Add to the result:0.0

How should the output value be rounded?:Not rounded

Enter how many decimal places the value should be rounded to:0

Constrain the result to a lower bound?:No

Enter the lower bound:0.0

Constrain the result to an upper bound?:No

Enter the upper bound:1.0

ExportToSpreadsheet:[module_num:9|svn_version:'Unknown'|variable_revision_number:13|show_window:True|notes:[]|batch_state:array([], dtype=uint8)|enabled:True|wants_pause:False]

Select the column delimiter:Comma (",")

Add image metadata columns to your object data file?:Yes

Add image file and folder names to your object data file?:No

Select the measurements to export:Yes

Calculate the per-image mean values for object measurements?:Yes

Calculate the per-image median values for object measurements?:No

Calculate the per-image standard deviation values for object measurements?:No

Output file location:Default Input Folder sub-folder|Desktop\\cellprofiler\\output

Create a GenePattern GCT file?:No

Select source of sample row name:Metadata

Select the image to use as the identifier:None

Select the metadata to use as the identifier:None

Export all measurement types?:Yes

Press button to select measurements:Image|Metadata_Condition,Image|Metadata_Experiment,Image|Metadata_Series,Image|Metadata_FileLocation,Image|Metadata_ROI,Image|Metadata_Protein,Image|Metadata_Well,Image|Metadata_Frame,Image|Intensity_TotalIntensity_589,Image|Intensity_MeanIntensity_589,Image|Count_Nuclei,Image|Math_IntensityPerCell,Nuclei|Number_Object_Number

Representation of Nan/Inf:NaN

Add a prefix to file names?:Yes

Filename prefix:Test1_

Overwrite existing files without warning?:No

Data to export:Do not use

Combine these object measurements with those of the previous object?:No

File name:DATA.csv

Use the object name for the file name?:Yes
